# One-Pot Synthesis of 1-Aryl-3-trifluoromethylpyrazoles Using Nitrile Imines and Mercaptoacetaldehyde As a Surrogate of Acetylene

**DOI:** 10.1021/acs.orglett.3c01437

**Published:** 2023-06-13

**Authors:** Kamil Świątek, Greta Utecht-Jarzyńska, Marcin Palusiak, Jun-An Ma, Marcin Jasiński

**Affiliations:** † Department of Organic and Applied Chemistry, Faculty of Chemistry, University of Lodz, Tamka 12, 91403 Łódź, Poland; ‡ Department of Physical Chemistry, Faculty of Chemistry, University of Lodz, Pomorska 163/165, 90236 Łódź, Poland; § Department of Chemistry, Tianjin Key Laboratory of Molecular Optoelectronic Sciences, Frontiers Science Center for Synthetic Biology (Ministry of Education), Tianjin University, Tianjin 300072, P. R. of China

## Abstract

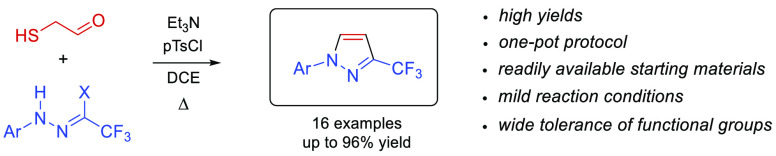

A synthetically useful approach for one-pot preparation of 1-aryl-3-trifluoromethylpyrazoles using in situ generated nitrile imines and mercaptoacetaldehyde applied as 1 equiv of acetylene is presented. This protocol comprises (3 + 3)-annulation of the mentioned reagents to form 5,6-dihydro-5-hydroxy-4*H*-1,3,4-thiadiazine, followed by cascade dehydration/ring contraction reactions with *p*-TsCl. In addition, representative nonfluorinated analogues functionalized with Ph, Ac, and CO_2_Et groups at the C(3)-position of the pyrazole ring were also prepared by the devised method.

1-Aryl-3-trifluoromethylpyrazole has been identified as a privileged structural motif for a number of bioactive compounds applied as either pharmaceutics or crop protection materials.^1^ For example, celecoxib is a well-known nonsteroidal anti-inflammatory agent (COX-2 inhibitor),^2^ whereas SC-560 exhibits anticancer activity (Figure 1).^3^ More recently introduced to the market, berotralstat acts as an effective plasma kallikrein inhibitor, and it is used in long-term prophylaxis to prevent hereditary angioedema (HAE) attacks.^4^ Furthermore, many 1-aryl-3-trifluoromethylpyrazoles also exhibit promising antibacterial^5^ and anticancer^6^ activity or serve as a key building block for the preparation of more advanced systems of interest in plant biology^7^ and material sciences.^8^ For these reasons, there is increasing interest in the development of new synthetic protocols to access 3-trifluoromethylpyrazoles of various substitution patterns, starting with readily available, cheap, and easy to handle building blocks.

**Figure 1:**
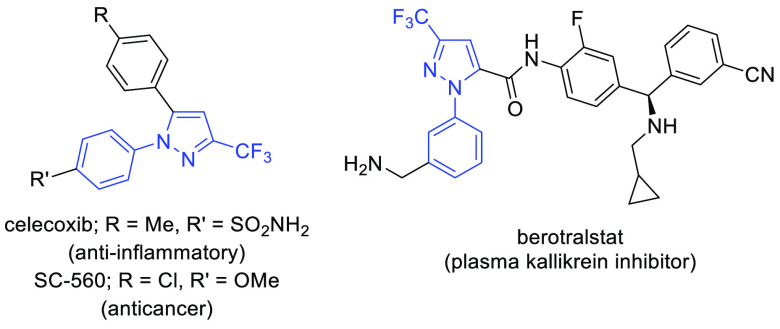
Structures of selected therapeutics based on a 1-aryl-3-trifluoromethylpyrazole core.

Among the methods available to date, classical condensation of trifluoromethylated 1,3-dicarbonyls or their equivalents with hydrazines is the most often applied strategy to access 3-trifluoromethylpyrazoles.^1,9^ Also, (3 + 2)-cycloadditions of fluorinated 1,3-dipoles such as 2,2,2-trifluorodiazoethane (CF_3_CHN_2_) with appropriate dipolarophiles have been shown to be a powerful approach.^10^ Importantly, rapid progress in the chemistry of trifluoroacetonitrile imines **1** (Scheme 1), recognized as readily available building blocks for the preparation of 3-CF_3_-pyrazoles, has been observed in recent years.^11,12^ Notably, application of this 1,3-dipole, readily accessible in situ via base-mediated dehydrohalogenation of the respective hydrazonoyl halides **2**, leads to N-functionalized heterocycles and typically offers excellent control on regio- and chemoselectivity of the cycloaddition step. For example, either electron-rich (vinyl ethers, enamines, and benzynes)^11^ or electron-deficient dipolarophiles (e.g., cyanoalkenes, nitroolefins, and enones)^12^ have been demonstrated as suitable reaction partners to access polyfunctionalized products.

**Scheme 1:**
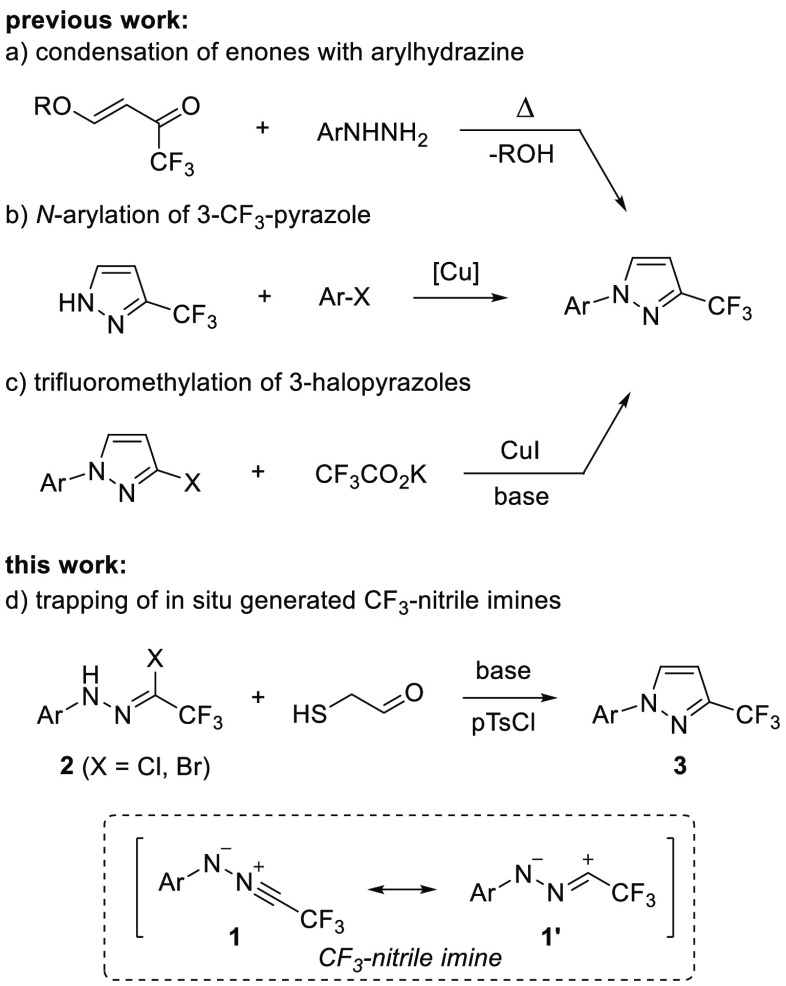
General Strategies for the Synthesis of 1-Aryl-3-trifluoromethylpyrazoles and the Method Reported Herein

The traditional methods toward simple 1-aryl-3-trifluoromethylpyrazoles **3** are limited to (a) cyclocondensations of appropriate trifluoromethylated carbonyl compounds^13^ with hydrazines and (b) Cu-catalyzed N-arylations of 3-trifluoromethyl-1*H*-pyrazole (Scheme 1).^14^ In addition, trifluoromethylation of 3-iodo-1-phenyl-1*H*-pyrazole under flow conditions is possible as reported by Buchwald (c).^15^ Nevertheless, the mentioned methods either suffer from regioselectivity issues or require harsh conditions and special equipment. Thus, nitrile imines **1** are revealed as ideal candidates for the preparation of such heterocyclic systems, e.g., through (3 + 2)-cycloaddition with acetylene^16^ or its surrogates.^17^

Here we report the one-pot synthesis of 1-aryl-3-trifluoromethylpyrazoles by using 2,5-dihydroxy-1,4-dithiane-2,5-diol as a convenient and safe material for the surrogate of acetylene. The method comprises (3 + 3)-condensation of in situ generated nitrile imine with mercaptoacetaldehyde, followed by *p*-TsCl-mediated dehydration and spontaneous or thermally induced Eschenmoser sulfide contraction^18^ of the first formed 4*H*-1,3,4-thiadiazine derivative (Scheme 2). In addition, a short series of nonfluorinated nitrile imines bearing an aryl (Ph), acyl (Ac), or ester (CO_2_Et) group at the C termini were checked to give the expected functionalized pyrazoles. Furthermore, some trifluoromethylated products of type **3** were applied for the preparation of three known drugs of selective COX-1 (SC-560) and COX-2 (Celecoxib and Mavacoxib) inhibitory activity.

**Scheme 2:**
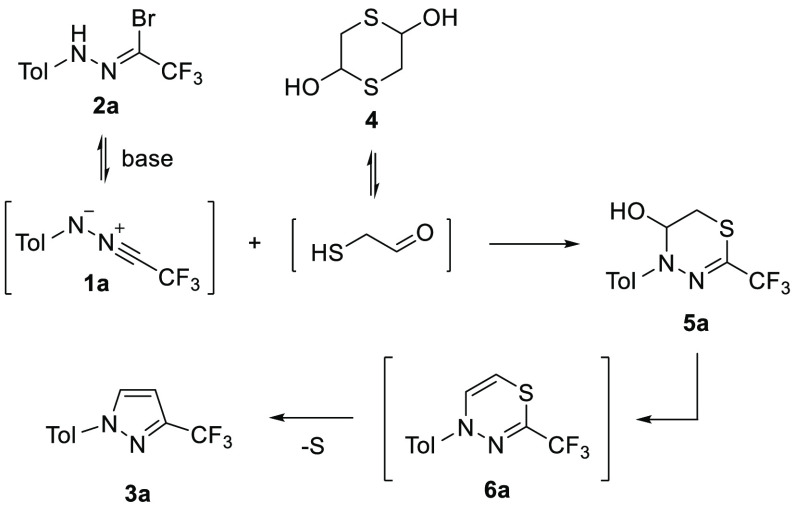
Synthesis of 1-Tolyl-3-trifluoromethylpyrazole (**3a**)

For the first experiments, commercially available 2,5-dihydroxy-1,4-dithiane-2,5-diol (**4**) as a precursor of mercaptoacetaldehyde^19^ and *N*-tolyl bromide **2a** as a precursor of nitrile imine **1a** were selected. Based on our previous experience in reactions of nitrile imines **1** with S-containing reaction partners,^20^ the reaction of **2a** and **4** (1:1) was carried out in dry dichloromethane, at room temperature, using Et_3_N as a base for in situ generation of nitrile imine, and the expected 5,6-dihydro-4*H*-1,3,4-thiadiazin-5-ol **5a** was formed solely under the applied conditions (Scheme 2).^20c^ In order to get more insight into the planned subsequent dehydration step, the first formed (3 + 3)-annulation product **5a** was isolated (95%) and briefly examined in a series of experiments (Table 1). Attempted activation of a hemiaminal group in **5a** with excess acetic acid (10 equiv) was in vain, and after standard aqueous workup the unchanged starting material was recovered (entry 1). In contrast, treatment of **5a** with aq. HCl (10 equiv, 18% aq) led to a mixture of starting material contaminated with small amounts of its 5-chloro analogue (in a ca. 4:1 ratio, respectively; entry 2). Similar results were noticed for the reaction carried out in a methanolic solution saturated with dry gaseous HCl (entry 3). Thus, substitution at C(5) was favored over the desired elimination pathway under the applied conditions. Also, treatment of **5a** with PPh_3_ (3.0 equiv) showed no changes in the mixture, even after 16 h of refluxing in THF (entry 4). Finally, activation of the OH group in **5a** with oxalyl chloride (1.5 equiv), in the presence of excess Et_3_N (3.0 equiv), led to a complex mixture of unidentified products, but the desired pyrazole **3a** was found as a minor component in 26% yield (entry 5). Gratifyingly, replacement of (COCl)_2_ with *p*-TsCl suppressed the formation of side products and provided the expected pyrazole **3a** (98%), which formed as the exclusive material (entry 6). Noteworthy, in the ^1^H NMR spectrum of the crude reaction mixture, no signals attributed to 1,3,4-thiadiazine intermediate **6a** could be detected, indicating that the anticipated ring contraction in **6a** proceeds smoothly at room temperature.

**Table 1:** Synthesis of Pyrazole **3a**: Optimization Study

entry	substrate(s)	additives	solvent	**3a** (%)
1	**5a**^a^	AcOH	CH_2_Cl_2_	-
2	**5a**^a^	HCl_(aq)_	CH_2_Cl_2_	-b
3	**5a**^a^	HCl_(dry)_	MeOH	-b
4	**5a**^a^^,^^c^	PPh_3_	THF	-
5	**5a**^a^	(COCl)_2_	CH_2_Cl_2_	26
6	**5a**^a^	*p*-TsCl	CH_2_Cl_2_	98
7	**2a** + **4**^d^	*p*-TsCl	CH_2_Cl_2_	67
8	**2a** + **4**^d^	*p*-TsCl^e^	CH_2_Cl_2_	94 (91)^f^
9	**2a** + **4**^d^	*p*-TsCl^e^	DCE	95
10	**2a** + **4**^d^	*p*-TsCl^e^	THF	88
11	**2a** + **4**^d^	*p*-TsCl^e^	toluene	81
12	**2a** + **4**^d^^,^^g^	*p*-TsCl^e^	CH_2_Cl_2_	95 (93)^f^

a Reaction conditions: a solution of **5a** (0.20 mmol) and additive (1.5, 3.0, or 10.0 equiv., see the text) in corresponding solvent (3 mL) were reacted overnight at room temperature; yields are estimated based on ^1^H NMR spectra of crude mixtures.

b An ca. 4:1 mixture of starting **5a** and its 5-chloro analogue was formed.

c Reflux.

d To a solution of **2a** (1.0 mmol) and **4** (0.55 mmol) in solvent (12 mL) was added Et_3_N (10.0 mmol). After 2 h, a solution of *p*-TsCl (1.5 mmol) in the same solvent (4 mL) was added dropwise, and the resulting mixture was stirred overnight; yields are estimated based on ^1^H NMR spectra of crude mixtures.

e 2.5 equiv of *p*-TsCl was used.

f Isolated yield.

g 7.1 mmol scale (starting with 2.0 g of bromide **2a**).

Next, the reaction was carried out in a one-pot manner starting with precursor **2a** and a slight excess of dimer **4**. After the bromide was fully consumed, excess *p*-TsCl (1.5 equiv) was added to the reaction mixture, and after 2 h the expected 1-tolyl-3-trifluoromethylpyrazole (**3a**, 67%) was detected in a crude mixture, along with unconsumed **5a** (entry 7). Further increase of the amount of *p*-TsCl (2.5 equiv) assured almost complete conversion, leading to **3a**, which was isolated in excellent 91% yield (entry 8), whereas the change of the reaction medium to nonhalogenated solvents such as THF or toluene slightly decreased conversion (entries 9–11). Finally, the reaction was repeated on a larger scale; starting with 2.0 g of bromide **2a** the target pyrazole **3a** (93%) was obtained in comparable yield (entry 12).

With the optimized conditions in hand, a series of trifluoromethylated hydrazonoyl bromides **2a**–**2p** were involved in the study. In all the cases the formation of the expected 4,5-unsubstituted pyrazoles was observed in high yields. Thus, alkyl (Me, *i*-Pr), alkoxy (OMe, OBn), and fluoroalkyl (CF_3_) substituents; halogens (F, Cl, Br); as well as functional groups such as cyano, nitro, sulfonamide, and ester could efficiently be introduced on the phenyl ring (Scheme 3). Notably, in the case of **2o** bearing the NO_2_ group, the respective 1,3,4-thiadiazine **6o** was found in a crude reaction mixture under the applied conditions. A small sample of this intermediate was isolated by preparative TLC and fully characterized by spectroscopic methods (Figure 2); however, its gradual desulfurization both during the NMR measurements (in CDCl_3_) and upon standing at room temperature was observed. Thus, the reactions of mercaptoacetaldehyde with strongly electron-deficient nitrile imines **1i** and **1k**–**1p** were carried out in DCE solutions, and the resulting mixtures were additionally heated under reflux to accelerate the final ring-contracting step.

**Scheme 3:**
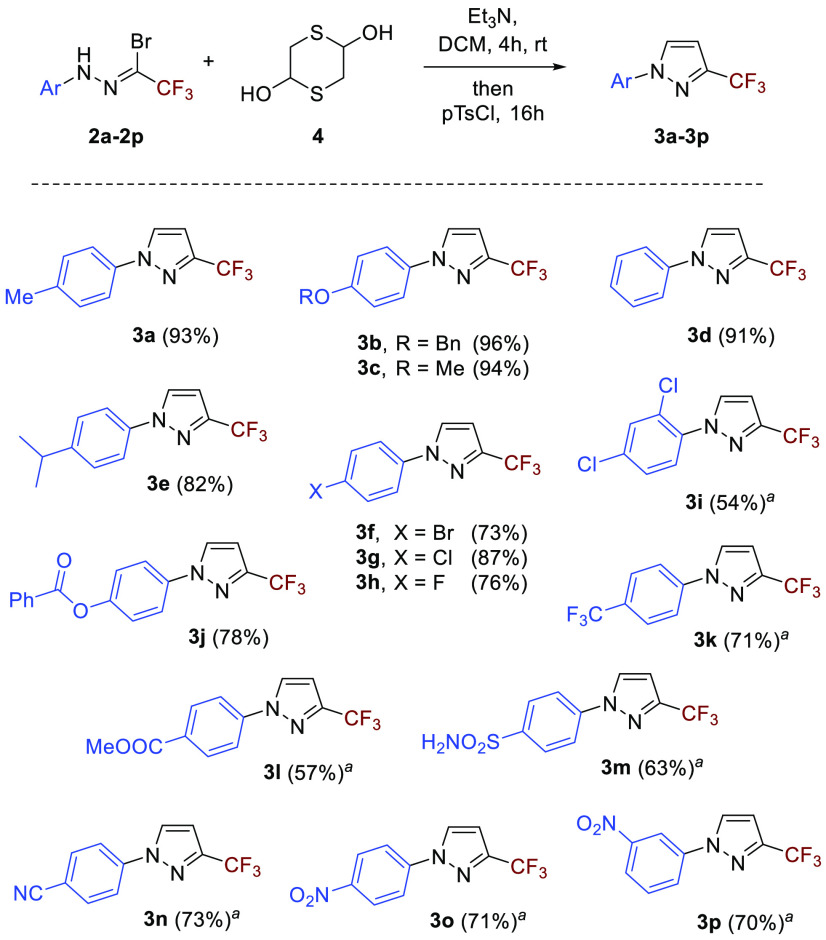
One-Pot Synthesis of 3-Trifluoromethylated 1-Arylpyrazoles **3a**–**3p**: Scope of Nitrile Imines DCE was used instead of DCM, and the resulting mixture was additionally refluxed for 2 h.

**Figure 2:**
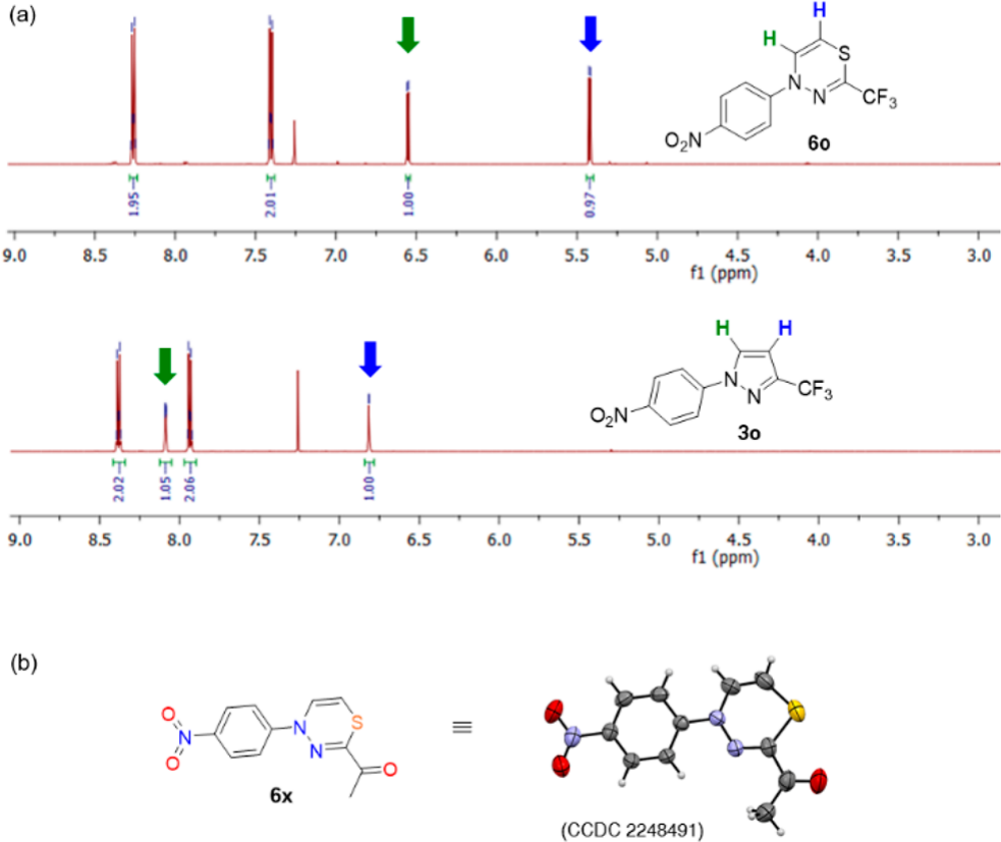
(a) Diagnostic chemical shifts in ^1^H NMR (600 MHz, CDCl_3_) of the vinylene unit in **6o** and **3o** and (b) X-ray structure of **6x**.

To briefly check the generality of the devised protocol, a series of selected nonfluorinated nitrile imines bearing Ph, CO_2_Et, or Ac groups at the C-termini of the 1,3-dipole were also examined in reaction with mercaptoacetaldehyde to provide the expected 1-arylpyrazoles **3q**–**3y** (Scheme 4). Introduction of both an electron-withdrawing acetyl group and the electron-deficient Ar substituent in chloride **2** (X = Cl, CO_2_Et, NO_2_) provided remarkably stable intermediate 1,3,4-thiadiazines **6**, which in comparison to CF_3_ analogues required harsher conditions (e.g., heating the crude mixture at 100 °C, either in DMSO or neat) to accomplish the final desulfurization. The structure of representative derivative **6x**, adopting well-defined boat-like conformation at the 4*H*-1,3,4-thiadiazine ring in the solid state (for details, see Supporting Information), was unambiguously confirmed by X-ray analysis (Figure 2). The possible mechanism of the studied ring contraction also deserves a comment. Based on the observed trends in stability of **6** and taking into account the noncatalyzed nature of the desulfuration step (proceeds upon standing), we assume that 1,3,4-thiadiazine **6** could undergo spontaneous 6π-electrocyclization, leading to bicyclic thiirane intermediate **A** (Scheme 5). Subsequent extrusion of the sulfur atom afforded aromatized pyrazole **3** as the final product.

**Scheme 4:**
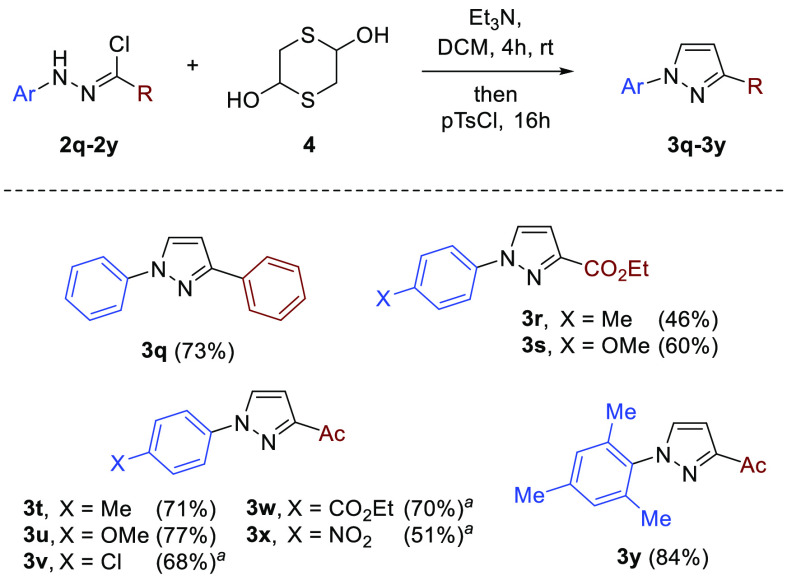
Synthesis of Nonfluorinated 1-Arylpyrazoles **3q**–**3y** Crude mixture was heated at 100 °C, in DMSO.

**Scheme 5:**
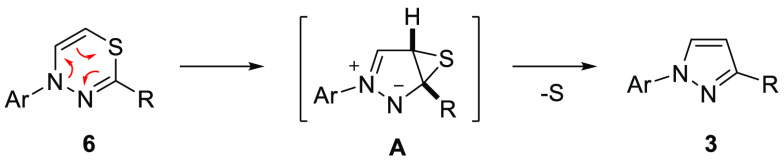
Proposed Mechanism

Finally, to utilize the devised method for the preparation of more complex pyrazole derivatives, compound **3c** was applied in the synthesis of SC-560. Selective deprotonation at C(5) of **3c** with *n*-BuLi, followed by trapping of the first formed anion with elemental iodine, afforded the respective iodide **7c** (91%). Subsequent Pd-catalyzed cross-coupling of the latter with 4-chlorophenylboronic acid provided SC-560 (**8**) in a high overall yield of 78% (Scheme 6). Similarly, sulfonamide-functionalized 3-trifluoromethylpyrazole **3m** was converted into the corresponding 5-iodo derivative **7m**; next, by using appropriate arylboronic acids, two further biologically active pyrazoles, namely, celecoxib (**9**) and mavacoxib (**10**), were obtained through Suzuki–Myiaura cross-coupling in 51% and 56% yield (for two steps), respectively.

**Scheme 6:**
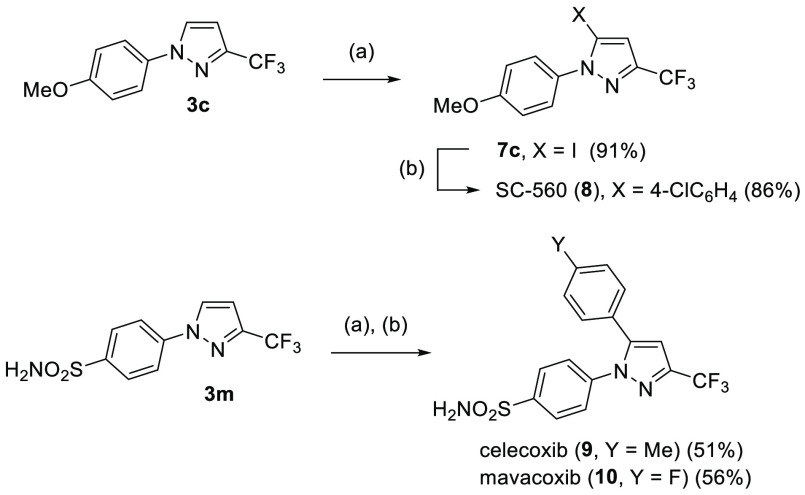
Synthesis of Celecoxib, Mavacoxib, and SC-560: (a) *n*-BuLi, TMEDA, and THF at −78 °C and Then I_2_ at rt for 30 min and (b) Arylboronic Acid, Pd(PPh_3_)_4_, K_2_CO_3_, and THF/H_2_O, at 80 °C, for 2 Days

In summary, a straightforward one-pot protocol for the preparation of 3-trifluoromethyl-1-arylpyrazoles using in situ generated nitrile imines and mercaptoacetaldehyde was developed. The presented method features readily available, safe, and easy to handle starting materials, mild reaction conditions, scalability, and wide tolerance of functional groups. It can also be applied for the synthesis of nonfluorinated analogues including derivatives bearing, at C(3) of the pyrazole ring, useful functional groups such as Ac and CO_2_Et. Hence, the current protocol can be recommended for the preparation of fluorinated and nonfluorinated 4,5-unsubstituted 1-arylpyrazoles, which not only are important structural scaffolds for numerous materials of practical significance but also can be considered as useful building blocks for postcyclizative functionalizations toward more complex pyrazole derivatives.

## Data Availability

The data underlying this study are available in the published article and its Supporting Information.

## Supporting Information Available

Experimental procedures, characterization data, and NMR spectra of all compounds (PDF)
FAIR data, including the primary NMR FID files, for compounds **2m**, **2v**–**2y**, **3a**–**3y**, **6o**, **6x**, **7c**, **7m**, and **8**–**11** (ZIP)
