# Supplementary material for: One-Pot Synthesis of 1-Aryl-3-trifluoromethylpyrazoles Using Nitrile Imines and Mercaptoacetaldehyde As a Surrogate of Acetylene
Source: Org Lett. 2023 Jun 13;25(24):4462–7. doi: 10.1021/acs.orglett.3c01437 (PMC10294255; doi:10.1021/acs.orglett.3c01437)
Supplement: Supplementary file 1 — ol3c01437_si_001.pdf [file ol3c01437_si_001.pdf]

Supporting Information

for

One-pot synthesis of 1-aryl-3-trifluoromethylpyrazoles using nitrile imines and mercaptoacetaldehyde as a surrogate of acetylene

Kamil Świątek,[a,b] Greta Utecht-Jarzyńska,[a] Marcin Palusiak,[c] Jun-An Ma,[d,*] Marcin Jasiński[a,*]

[a] *Department of Organic and Applied Chemistry, Faculty of Chemistry, University of Lodz, Tamka 12, 91403 Łódź, Poland*
[b] *The University of Lodz Doctoral School of Exact and Natural Sciences, Banacha 12/16, 90237 Łódź, Poland*
[c] *Department of Physical Chemistry, Faculty of Chemistry, University of Lodz, Pomorska 163/165, 90236 Łódź, Poland*
[d] *Department of Chemistry, Tianjin Key Laboratory of Molecular Optoelectronic Sciences, Frontiers Science Center for Synthetic Biology (Ministry of Education), Tianjin University, Tianjin 300072, P. R. of China.*

* Corresponding authors:

Jun-An Ma – *Department of Chemistry, Tianjin University, Tianjin 300072, P. R. of China;* https://orcid.org/0000-0002-3902-6799; Tel: 0086-22-27407286; Fax: 0086-22-27403475; E-mail: majun_an68@tju.edu.cn

Marcin Jasiński – *Faculty of Chemistry, University of Lodz, 91403 Łódź, Poland;* https://orcid.org/0000-0002-8789-9690; Tel: 48-42-6355766; Email: marcin.jasinski@chemia.uni.lodz.pl

Content

1. General information S2

2. Synthetic procedures and characterization data S3

3. Copies of NMR spectra S16

4. Crystallographic analysis S56

5. References S60

1. General information

Experimental procedures: All commercially available starting materials were used as received. If not stated otherwise, reactions were carried out under inert atmosphere (argon), in flame-dried flasks; subsequent manipulations were conducted in air. DCM was dried over CaH_2 and freshly distilled before use; THF was dried over sodium/benzophenone and freshly distilled before use; other anhydrous solvents (DCE, toluene) were purchased and used as received. Products were purified by filtration through short silica gel plug (flash CC) or by standard column chromatography (CC) on silica gel (230–400 mesh) by using freshly distilled solvents as eluents or by recrystallization from appropriate solvents. NMR spectra were taken on a Bruker AVIII instrument (^{1}H at 600 MHz, ^{13}C at 151 MHz, and ^{19}F at 565 MHz); chemical shifts are reported relative to solvent residual peaks [for $CDCl_3$: ^{1}H NMR: δ = 7.26, ^{13}C NMR: δ = 77.16; for DMSO-d_6: ^{1}H NMR: δ = 2.50, ^{13}C NMR: δ = 39.52][1] or to $CFCl_3$ (^{19}F NMR: δ = 0.00) used as external standard. Structural assignments were made with additional information from gCOSY, gHSQC, and gHMBC experiments. The IR spectra were taken with an Agilent Cary 630 FTIR spectrometer, in neat. ESI-MS were performed with a Varian 500-MS LC Ion Trap; high resolution MS (ESI-TOF) measurements were performed with a Synapt G2-Si mass spectrometer (Waters). Combustion analyses were obtained with a Vario EL III (Elementar Analysensysteme GmbH) instrument. Melting points were determined in capillaries with a MEL-TEMP apparatus (Laboratory Devices), and are uncorrected.

Starting materials: The starting trifluoromethylated nitrile imine precursors, i.e. hydrazonoyl bromides **2a-2p**, were prepared according to general literature protocol, through NBS-mediated bromination of the corresponding fluoral arylhydrazones, in dry DMF at room temperature.[2] The required hydrazones were obtained by heating methanolic solution of aqueous fluoral hydrate (~75%, in H_2O) with hydrazines, in a closed ampoule at 75 °C, in the presence of molecular sieves 4Å, as reported.[3] Non-fluorinated hydrazonoyl chlorides **2q**[4] and **2r**-**2y**[5] were prepared in analogy to the general literature protocols, by chlorination of benzoyl phenylhydrazine with Ph_3P-CCl_4 or by coupling of α-chloromethinyl compounds with arene-diazonium salts under basic conditions, respectively.

2. Synthetic procedures and characterization data

General one-pot procedure for synthesis of pyrazoles 3a-3y: To a solution of the respective hydrazonoyl halide **2** (1.0 mmol) and 2,5-dihydroxy-1,4-dithiane-2,5-diol (**4**, 0.55 mmol, 84 mg) in dry solvent (10 mL; DCM for **2a**-**2h**,**2q**-**2u**, and **2v**-**2y**; DCE for **2i**-**2p**) was added dropwise excess triethylamine (10 mmol, 1.4 mL) and the resulting mixture was stirred at room temperature until the nitrile imine precursor **2** was fully consumed (TLC monitoring, typically 2-4 h). Then, a solution of *p*-toluenesulfonic chloride (2.5 mmol, 477 mg) in appropriate solvent (5 mL; DCM or DCE, respectively) was added dropwise and stirring was continued for 16 h. Water (15 mL) was added, the mixture was extracted with DCM (3 × 10 mL), the combined organic layers were dried over anhydrous Na_2SO_4, filtered, and the solvents were roved under reduced pressure. The crude product **3** was purified by column chromatography (CC) using silica gel as the stationary phase and hexanes/DCM or hexanes/EtOAc mixtures as an eluent.
In the case of **2i**-**2p**, the resulting mixture was additionally refluxed (oil bath) for 2 h prior aqueous workup. In the case of **2v**-**2x**, the solvent was removed in vacuo, the resulting was dissolved in DMSO and heated at 100 °C (oil bath) for 2 h, followed by standard aqueous workup.

1-(4-Tolyl)-3-trifluoromethyl-1*H*-pyrazole (**3a**):

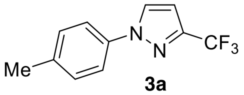

3a

CC (SiO_2, hexanes/DCM 1:1); colorless solid, 210 mg (93%); mp 52-53 °C. ^{1}H NMR (600 MHz, $CDCl_3$) δ 7.90 (m$_c$, 1H, 5-H), 7.59-7.56 (m, 2H), 7.29-7.26 (m, 2H), 6.70 (d$_{br}$, $J \approx 2.5$ Hz, 1H, 4-H), 2.40 (s, 3H). ^{13}C{^{1}H} NMR (151 MHz, $CDCl_3$) δ 143.8 (q, $^2J_{C\text{-}F}$ = 38.5 Hz, C-3), 137.9, 137.3, 130.2, 128.4 (C-5), 121.4 (q, $^1J_{C\text{-}F}$ = 268.6 Hz, CF_3), 120.0, 105.9 (q, $^3J_{C\text{-}F}$ = 2.2 Hz, C-4), 21.1. ^{19}F NMR (565 MHz, $CDCl_3$) δ –62.01 (s, CF_3). IR (neat) ν 1510, 1387, 1275, 1126, 1059, 969 cm^{-1}. ESI-MS (*m/z*): 227.2 (100, $[M+H]^+$). Anal. calcd for $C_{11}H_9F_3N_2$ (226.2): C 58.41, H 4.01, N 12.38; found: C 58.32, H 4.11, N 12.64.

1-(4-Benzyloxyphenyl)-3-trifluoromethyl-1*H*-pyrazole (**3b**):

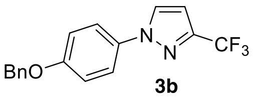

3b

CC (SiO_2, hexanes/DCM 2:3); colorless solid, 305 mg (96%); mp 98-99 °C. ^{1}H NMR (600 MHz, $CDCl_3$) δ 7.84 (m$_c$, 1H, 5-H), 7.61-7.59 (m, 2H), 7.46-7.33 (m, 5H), 7.08-7.05 (m, 2H), 6.69 (d$_{br}$, $J \approx 2.4$ Hz, 1H, 4-H), 5.11 (s, 2H). ^{13}C{^{1}H} NMR (151 MHz, $CDCl_3$) δ 158.4, 143.7 (q, $^2J_{C\text{-}F}$ = 38.3 Hz, C-3), 136.6, 133.4, 128.8, 128.5 (C-5), 128.3, 127.6, 121.8, 121.4 (q, $^1J_{C\text{-}F}$ = 268.6 Hz, CF_3), 115.8, 105.8 (q, $^3J_{C\text{-}F}$ = 2.0 Hz, C-4), 70.5. ^{19}F NMR (565 MHz, $CDCl_3$) δ –61.96 (s, CF_3). IR (neat) ν 1510, 1379, 1241, 1170, 1054, 1010 cm^{-1}. ESI-MS (*m/z*): 319.2 (100, $[M+H]^+$). Anal. calcd for $C_{17}H_{13}F_3N_2O$ (318.1): C 64.15, H 4.12, N 8.80; found: C 64.17, H 4.19, N 8.91.

1-(4-Methoxyphenyl)-3-trifluoromethyl-1*H*-pyrazole (**3c**):

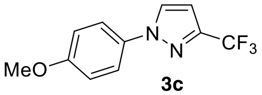

3c

CC (SiO_2, hexanes/EtOAc 9:1); light orange oil, 227 mg (94%). ^{1}H NMR (600 MHz, $CDCl_3$) δ 7.84 (m$_c$, 1H, 5-H), 7.61-7.58 (m, 2H), 7.00-6.97 (m, 2H), 6.69 (d$_{br}$, $J \approx 2.4$ Hz, 1H, 4-H), 3.85 (s, 3H). ^{13}C{^{1}H} NMR (151 MHz, $CDCl_3$) δ 159.3, 143.7 (q, $^2J_{C\text{-}F} = 38.4$ Hz, C-3), 133.2, 128.5 (C-5), 121.8, 121.4 (q, $^1J_{C\text{-}F} = 268.6$ Hz, CF_3), 114.8, 105.8 (q, $^3J_{C\text{-}F} = 2.3$ Hz, C-4), 55.7. ^{19}F NMR (565 MHz, $CDCl_3$) δ –61.96 (s, CF_3). IR (neat) ν 1510, 1387, 1249, 1170, 1126, 1055 cm^{-1}. ESI-MS (*m/z*): 243.2 (100, [M+H]$^+$). Anal. calcd for $C_{11}H_9F_3N_2O$ (242.1): C 54.55, H 3.75, N 11.57; found: C 54.56, H 3.90, N 11.37.

1-Phenyl-3-trifluoromethyl-1*H*-pyrazole[6] (**3d**):

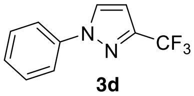

3d

CC (SiO_2, hexanes/DCM 3:2); thick yellow oil, 193 mg (91%). ^{1}H NMR (600 MHz, $CDCl_3$) δ 7.95 (m$_c$, 1H, 5-H), 7.72-7.70 (m, 2H), 7.50-7.47 (m, 2H), 7.39-7.36 (m, 1H), 6.72 (d$_{br}$, $J \approx 2.4$ Hz, 1H, 4-H). ^{13}C{^{1}H} NMR (151 MHz, $CDCl_3$) δ 144.1 (q, $^2J_{C\text{-}F} = 38.4$ Hz, C-3), 139.5, 129.7, 128.5 (C-5), 127.9, 121.4 (q, $^1J_{C\text{-}F} = 268.8$ Hz, CF_3), 120.1, 106.1 (q, $^3J_{C\text{-}F} = 1.9$ Hz, C-4). ^{19}F NMR (565 MHz, $CDCl_3$) δ –62.07 (s, CF_3). IR (neat) ν 1513, 1358, 1250, 1126, 1094, 1021 cm^{-1}. ESI-MS (*m/z*): 213.1 (100, [M+H]$^+$).

1-(4-Isopropylphenyl)-3-trifluoromethyl-1*H*-pyrazole (**3e**):

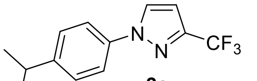

3e

CC (SiO_2, hexanes/EtOAc 9:1); light orange solid, 208 mg (82%); mp 49-51 °C. ^{1}H NMR (600 MHz, $CDCl_3$) δ 7.90 (m$_c$, 1H, 5-H), 7.62-7.59 (m, 2H), 7.34-7.32 (m, 2H), 6.70 (d$_{br}$, $J \approx 2.3$ Hz, 1H, 4-H), 2.96 (hept, $J = 6.9$ Hz, 1H), 1.28 (d, $J = 6.9$ Hz, 6H). ^{13}C{^{1}H} NMR (151 MHz, $CDCl_3$) δ 148.9, 143.9 (q, $^2J_{C\text{-}F} = 38.4$ Hz, C-3), 137.6, 128.4 (C-5), 127.7, 121.4 (q, $^1J_{C\text{-}F} = 268.7$ Hz, CF_3), 120.2, 105.9 (q, $^3J_{C\text{-}F} = 2.4$ Hz, C-4), 33.9, 24.1. ^{19}F NMR (565 MHz, $CDCl_3$) δ –62.03 (s, CF_3). IR (neat) ν 1513, 1387, 1271, 1126, 1059 cm^{-1}. ESI-MS (*m/z*): 255.2 (100, [M+H]$^+$). Anal. calcd for $C_{13}H_{13}F_3N_2$ (254.1): C 61.41, H 5.15, N 11.02; found: C 61.43, H 5.30, N 10.77.

1-(4-Bromophenyl)-3-trifluoromethyl-1*H*-pyrazole (**3f**):

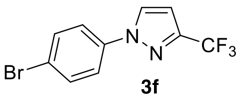

3f

CC (SiO_2, hexanes/DCM 9:1); thick yellow oil, 212 mg (73%). ^{1}H NMR (600 MHz, $CDCl_3$) δ 7.93 (m$_c$, 1H, 5-H), 7.60 (s$_{br}$, 4H), 6.73 (d$_{br}$, $J \approx 2.5$ Hz, 1H, 4-H). ^{13}C{^{1}H} NMR (151 MHz, $CDCl_3$) δ 144.5 (q, $^2J_{C\text{-}F} = 38.6$ Hz, C-3), 138.5, 132.8, 128.4 (C-5), 121.41, 121.38, 121.2 (q, $^1J_{C\text{-}F} = 268.9$ Hz, CF_3), 106.5 (q, $^3J_{C\text{-}F} = 1.8$ Hz, C-4). ^{19}F NMR (565 MHz, $CDCl_3$) δ –62.21 (s, CF_3). IR (neat) ν 1495, 1390, 1275, 1170, 1129, 1051 cm^{-1}. ESI-MS (*m/z*): 293.1 (99, [M{^{81}Br}+H]$^+$), 291.1 (100, [M{^{79}Br}+H]$^+$). Anal. calcd for $C_{10}H_6BrF_3N_2$ (291.1): C 41.26, H 2.08, N 9.62; found: C 41.19, H 2.24, N 9.68.

1-(4-Chlorophenyl)-3-trifluoromethyl-1*H*-pyrazole (**3g**):

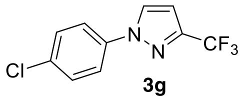

3g

CC (SiO_2, hexanes/DCM 3:2); colorless solid, 214 mg (87%); mp 52-53 °C. ^{1}H NMR (600 MHz, $CDCl_3$) δ 7.92 (m_c, 1H, 5-H), 7.67-7.64 (m, 2H), 7.46-7.44 (m, 2H), 6.73 (d_{br}, $J \approx 2.5$ Hz, 1H, 4-H). $^{13}C\{^{1}H\}$ NMR (151 MHz, $CDCl_3$) δ 144.4 (q, $^{2}J_{C\text{-}F}$ = 38.6 Hz, C-3), 130.1, 133.6, 129.9, 128.4 (C-5), 121.2 (q, $^{1}J_{C\text{-}F}$ = 268.9 Hz, CF_3), 121.1, 106.5 (q, $^{3}J_{C\text{-}F}$ = 2.2 Hz, C-4). ^{19}F NMR (565 MHz, $CDCl_3$) δ –62.19 (s, CF_3). IR (neat) ν 1495, 1387, 1275, 1178, 1122, 1096, 1055 cm^{-1}. ESI-MS (*m/z*): 249.3 (41, $[M\{^{37}Cl\}+H]^+$), 247.3 (100, $[M\{^{35}Cl\}+H]^+$). Anal. calcd for $C_{10}H_6ClF_3N_2$ (246.6): C 48.70, H 2.45, N 11.36; found: C 48.53, H 2.61, N 11.41.

1-(4-Fluorophenyl)-3-trifluoromethyl-1*H*-pyrazole (**3h**):

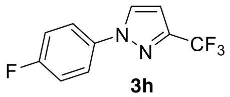

3h

CC (SiO_2, hexanes/DCM 4:1); light orange solid, 175 mg (76%); mp 60-61 °C. ^{1}H NMR (600 MHz, $CDCl_3$) δ 7.89 (m_c, 1H, 5-H), 7.69-7.66 (m, 2H), 7.19-7.17 (m, 2H), 6.72 (d_{br}, $J \approx 2.5$ Hz, 1H, 4-H). $^{13}C\{^{1}H\}$ NMR (151 MHz, $CDCl_3$) δ 162.0 (d, $^{1}J_{C\text{-}F}$ = 247.9 Hz), 144.3 (q, $^{2}J_{C\text{-}F}$ = 38.5 Hz, C-3), 135.9 (d, $^{4}J_{C\text{-}F}$ = 3.1 Hz), 128.6 (C-5), 122.0 (d, $^{3}J_{C\text{-}F}$ = 8.6 Hz), 121.3 (q, $^{1}J_{C\text{-}F}$ = 268.8 Hz, CF_3), 116.6 (d, $^{2}J_{C\text{-}F}$ = 23.3 Hz), 106.2 (q, $^{3}J_{C\text{-}F}$ = 2.2 Hz, C-4). ^{19}F NMR (565 MHz, $CDCl_3$) δ –62.13 (s, CF_3), –113.82 (m_c, Ar-F). IR (neat) ν 1510, 1390, 1275, 120, 1170, 1118, 1062 cm^{-1}. ESI-MS (*m/z*): 231.2 (100, $[M+H]^+$). Anal. calcd for $C_{10}H_6F_4N_2$ (230.2): C 52.18, H 2.63, N 12.17; found: C 52.37, H 2.79, N 11.99.

1-(2,4-Dichlorophenyl)-3-trifluoromethyl-1*H*-pyrazole (**3i**):

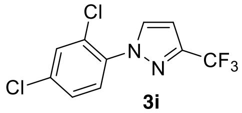

3i

CC (SiO_2, hexanes/DCM 1:1); brown oil, 151 mg (54%). ^{1}H NMR (600 MHz, $CDCl_3$) δ 7.89 (m_c, 1H, 5-H), 7.54-7.56 (m, 2H), 7.39 (dd, J = 2.3, 8.6 Hz, 1H), 6.73 (d_{br}, $J \approx 2.5$ Hz, 1H, 4-H). $^{13}C\{^{1}H\}$ NMR (151 MHz, $CDCl_3$) δ 144.4 (q, $^{2}J_{C\text{-}F}$ = 38.6 Hz, C-3), 136.1, 135.6, 133.1 (C-5), 130.6, 129.5, 128.9, 128.3, 121.2 (q, $^{1}J_{C\text{-}F}$ = 268.9 Hz, CF_3), 105.5 (q, $^{3}J_{C\text{-}F}$ = 2.2 Hz, C-4). ^{19}F NMR (565 MHz, $CDCl_3$) δ –62.13 (s, CF_3). IR (neat) ν 1487, 1387, 1275, 1170, 1129, 1040, 950 cm^{-1}. ESI-MS (*m/z*): 281.2 (100, $[M+H]^+$), 283.2 (61). Anal. calcd for $C_{10}H_5Cl_2F_3N_2$ (281.1): C 42.73, H 1.79, N 9.97; found: C 42.78, H 2.07, N 9.98.

4-(3'-Trifluoromethyl-1'*H*-pyrazol-1'-yl)phenyl benzoate (**3j**):

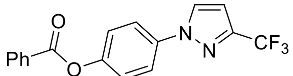

3j

CC (SiO_2, hexanes/DCM 2:3); light yellow solid, 259 mg (78%); mp 141-143 °C. ^{1}H NMR (600 MHz, $CDCl_3$) δ 8.23-8.21 (m, 2H), 7.95 (m_c, 1H, 5'-H), 7.78-7.76 (m, 2H), 7.68-7.65 (m, 1H), 7.55-7.52 (m, 2H), 7.38-7.35 (m, 2H), 6.74 (d_{br}, $J \approx 2.3$ Hz, 1H, 4'-H). $^{13}C\{^{1}H\}$ NMR (151 MHz, $CDCl_3$) δ 165.1, 150.3, 144.3 (q, $^{2}J_{C\text{-}F}$ = 38.5 Hz, C-3'), 137.2, 134.0, 130.4, 129.2, 128.8, 128.6, 123.1, 121.3

(q, $^{1}J_{C-F}$ = 268.8 Hz, CF_3), 121.2, 106.3 (q, $^{3}J_{C-F}$ = 2.2 Hz, C-4’). ^{19}F NMR (565 MHz, $CDCl_3$) δ –62.11 (s, CF_3). IR (neat) ν 1722, 1513, 1387, 1267, 1204, 1118, 1059 cm^{-1}. ESI-MS (*m/z*): 371.3 (49, $[M+K]^+$), 355.3 (100, $[M+Na]^+$). Anal. calcd for $C_{17}H_{11}F_3N_2O_2$ (332.3): C 61.45, H 3.34, N 8.43; found: C 61.58, H 3.36, N 8.37.

3-Trifluoromethyl-1-(4-trifluoromethylphenyl)-1*H*-pyrazole (**3k**):

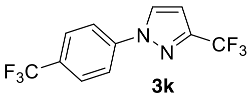

3k

CC (SiO_2, hexanes/DCM 9:1); yellow oil, 199 mg (71%). ^{1}H NMR (600 MHz, $CDCl_3$) δ 8.03 (m_c, 1H, 5-H), 7.88-7.85 (m, 2H), 7.77-7.75 (m, 2H), 6.77 (d_{br}, $J \approx 2.5$ Hz, 1H, 4-H). ^{13}C{^{1}H} NMR (151 MHz, $CDCl_3$) δ 145.0 (q, $^{2}J_{C-F}$ = 38.7 Hz, C-3), 141.9, 129.8 (q, $^{2}J_{C-F}$ = 33.1 Hz, *i*-C), 128.5 (C-5), 127.1 (q, $^{3}J_{C-F}$ = 3.7 Hz, 2CH), 123.8 (q, $^{1}J_{C-F}$ = 272.1 Hz, CF_3), 121.1 (q, $^{1}J_{C-F}$ = 268.9 Hz, 3-CF_3), 119.8, 106.9 (q, $^{3}J_{C-F}$ = 2.4 Hz, C-4). ^{19}F NMR (565 MHz, $CDCl_3$) δ –62.47, –62.35 (2s, 2CF_3). IR (neat) ν 1394, 1323, 1275, 1122, 1051 cm^{-1}. ESI-MS (*m/z*): 281.3 (100, $[M+H]^+$). Anal. calcd for $C_{11}H_6F_6N_2$ (280.2): C 47.16, H 2.16, N 10.00; found: C 47.21, H 2.36, N 9.87.

1-(4-Methoxycarbonylphenyl)-3-trifluoromethyl-1*H*-pyrazole (**3l**):

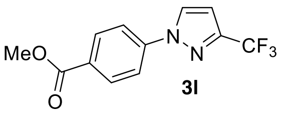

3l

CC (SiO_2, hexanes/EtOAc 4:1); orange solid, 154 mg (57%); mp 83-84 °C. ^{1}H NMR (600 MHz, $CDCl_3$) δ 8.18-8.16 (m, 2H), 8.04 (m_c, 1H, 5-H), 7.83-7.80 (m, 2H), 6.77 (d_{br}, $J \approx$ 2.5 Hz, 1H, 4-H), 3.95 (s, 3H). ^{13}C{^{1}H} NMR (151 MHz, $CDCl_3$) δ 166.2, 144.9 (q, $^{2}J_{C-F}$ = 38.7 Hz, C-3), 142.6, 131.4, 129.4, 128.5 (C-5), 121.1 (q, $^{1}J_{C-F}$ = 268.8 Hz, CF_3), 119.3, 106.8 (q, $^{3}J_{C-F}$ = 1.6 Hz, C-4), 52.5. ^{19}F NMR (565 MHz, $CDCl_3$) δ –62.32 (s, CF_3). IR (neat) ν 1711, 1607, 1394, 1275, 1152, 1107, 1051 cm^{-1}. ESI-MS (*m/z*): 271.3 (100, $[M+H]^+$). Anal. calcd for $C_{12}H_9F_3N_2O_2$ (270.2): C 53.34, H 3.36, N 10.37; found: C 53.53, H 3.48, N 10.16.

4-(3’-Trifluoromethyl-1’*H*-pyrazol-1’-yl)benzenesulfonamide (**3m**):

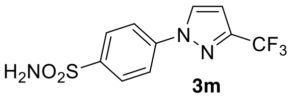

3m

CC (SiO_2, hexanes/EtOAc 1:1); brown solid, 183 mg (63%); mp 163-165 °C. ^{1}H NMR (600 MHz, DMSO-d_6) δ 8.86 (m_c, 1H, 5’-H), 8.12-8.09 (m, 2H), 7.99-7.97 (m, 2H), 7.50 (s_{br}, 2H), 7.13 (d_{br}, $J \approx 2.5$ Hz, 1H, 4’-H). ^{13}C{^{1}H} NMR (151 MHz, DMSO-d_6) δ 143.1 (q, $^{2}J_{C-F}$ = 37.8 Hz, C-3’), 143.0, 140.8, 131.2, 127.4, 121.2 (q, $^{1}J_{C-F}$ = 268.6 Hz, CF_3), 119.5, 106.9 (q, $^{3}J_{C-F}$ = 2.2 Hz, C-4’). ^{19}F NMR (565 MHz, DMSO-d_6) δ –60.81 (s, CF_3). IR (neat) ν 3265, 1599, 1502, 1394, 1327, 1282, 1155, 1115, 1059 cm^{-1}. ESI-MS (*m/z*): 314.2 (94, $[M+Na]^+$), 292.2 (100, $[M+H]^+$). Anal. calcd for $C_{10}H_8F_3N_3O_2S$ (291.2): C 41.24, H 2.77, N 14.43, S 11.01; found: C 41.00, H 2.80, N 14.54, S 11.13.

4-(3'-Trifluoromethyl-1'*H*-pyrazol-1'-yl)benzonitrile (**3n**):

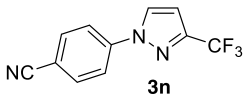

3n

CC (SiO_2, hexanes/DCM 3:2); colorless solid, 173 mg (73%); mp 112-113 °C. ^{1}H NMR (600 MHz, $CDCl_3$) δ 8.04 (dq, J = 0.9, 2.7 Hz, 1H, 5'-H), 7.90-7.87 (m, 2H), 7.81-7.79 (m, 2H), 6.79 (d_{br}, $J \approx 2.6$ Hz, 1H, 4'-H). $^{13}C\{^{1}H\}$ NMR (151 MHz, $CDCl_3$) δ 145.4 (q, $^{2}J_{C-F}$ = 38.9 Hz, C-3'), 142.3, 134.0, 128.5 (C-5'), 121.0 (q, $^{1}J_{C-F}$ = 269.0 Hz, CF_3), 119.9, 118.1, 111.4, 107.3 (q, $^{3}J_{C-F}$ = 1.7 Hz, C-4'). ^{19}F NMR (565 MHz, $CDCl_3$) δ –62.48 (s, CF_3). IR (neat) ν 2229, 1606, 1506, 1390, 1279, 1174, 1122, 1051 cm^{-1}. ESI-MS (*m/z*): 238.3 (100, $[M+H]^+$). Anal. calcd for $C_{11}H_6F_3N_3$ (237.2): C 55.70, H 2.55, N 17.72; found: C 55.70, H 2.72, N 17.75.

1-(4-Nitrophenyl)-3-trifluoromethyl-1*H*-pyrazole (**3o**):

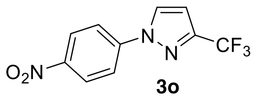

3o

CC (SiO_2, hexanes/DCM 1:1); colorless solid, 183 mg (71%); mp 116-117 °C. ^{1}H NMR (600 MHz, $CDCl_3$) δ 8.40-8.37 (m, 2H), 8.09 (dq, J = 0.9, 2.6 Hz, 1H, 5-H), 7.96-7.92 (m, 2H), 6.82 (d_{br}, $J \approx 2.6$ Hz, 1H, 4-H). $^{13}C\{^{1}H\}$ NMR (151 MHz, $CDCl_3$) δ 146.6, 145.7 (q, $^{2}J_{C-F}$ = 39.0 Hz, C-3), 143.6, 128.8 (C-5), 125.6, 120.9 (q, $^{1}J_{C-F}$ = 269.2 Hz, CF_3), 119.7, 107.5 (q, $^{3}J_{C-F}$ = 2.1 Hz, C-4). ^{19}F NMR (565 MHz, $CDCl_3$) δ –62.51 (s, CF_3). IR (neat) ν 1599, 1521, 1342, 1275, 1170, 1133, 1048 cm^{-1}. ESI-MS (*m/z*): 258.2 (100, $[M+H]^+$). Anal. calcd for $C_{10}H_6F_3N_3O_2$ (257.2): C 46.70, H 2.35, N 16.34; found: C 46.76, H 2.62, N 16.32.

1-(3-Nitrophenyl)-3-trifluoromethyl-1*H*-pyrazole (**3p**):

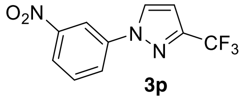

3p

CC (SiO_2, hexanes/DCM 3:2); light yellow solid, 179 mg (70%); mp 87-89 °C. ^{1}H NMR (600 MHz, $CDCl_3$) δ 8.58 (t, J = 2.2 Hz, 1H), 8.24 (ddd, J = 0.9, 2.2, 8.3 Hz, 1H), 8.14 (ddd, J = 0.9, 2.3, 8.1 Hz, 1H), 8.08 (m_c, 1H, 5-H), 7.71 (t_{br}, $J \approx 8.2$ Hz, 1H), 6.81 (d_{br}, $J \approx 2.6$ Hz, 1H, 4-H). $^{13}C\{^{1}H\}$ NMR (151 MHz, $CDCl_3$) δ 149.1, 145.2 (q, $^{2}J_{C-F}$ = 38.9 Hz, C-3), 140.2, 130.9, 128.6 (C-5), 125.3, 122.3, 121.0 (q, $^{1}J_{C-F}$ = 269.1 Hz, CF_3), 114.6, 107.2 (q, $^{3}J_{C-F}$ = 2.2 Hz, C-4). ^{19}F NMR (565 MHz, $CDCl_3$) δ –62.41 (s, CF_3). IR (neat) ν 1536, 1476, 1349, 1305, 1271, 1193, 1133, 1055 cm^{-1}. ESI-MS (*m/z*): 258.2 (100, $[M+H]^+$). Anal. calcd for $C_{10}H_6F_3N_3O_2$ (257.2): C 46.70, H 2.35, N 16.34; found: C 46.69, H 2.46, N 16.31.

1,3-Diphenyl-1*H*-pyrazole[7] (**3q**):

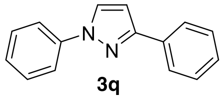

3q

CC (SiO_2, hexanes/DCM 3:2); pale yellow solid, 161 mg (73%); mp 81-82 °C (mp 80-82 °C)[7a]. ^{1}H NMR (600 MHz, $CDCl_3$) δ 7.96 (d, J = 2.5 Hz, 1H, 5-H), 7.94-7.91 (m, 2H), 7.80-7.77

(m, 2H), 7.49-7.42 (m, 4H), 7.36-7.28 (m, 2H), 6.79 (d, *J* = 2.5 Hz, 1H, 4-H). $^{13}C\{^1H\}$ NMR (151 MHz, $CDCl_3$) *δ* 153.1, 140.4, 133.3, 129.5, 128.8, 128.2, 128.1, 126.5, 126.0, 119.2. ESI-MS (*m/z*): 221.2 (100, $[M+H]^+$).

3-Ethoxycarbonyl-1-(4-tolyl)-1*H*-pyrazole (**3r**):

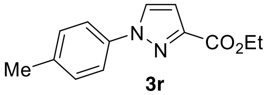

3r

CC (SiO_2, hexanes/EtOAc 4:1); thick orange oil, 106 mg (46%). ^1H NMR (600 MHz, $CDCl_3$) *δ* 7.88 (d, *J* = 2.5 Hz, 1H, 5-H), 7.62-7.60 (m, 2H), 7.26-7.24 (m, 2H), 6.97 (d, *J* = 2.5 Hz, 1H, 4-H), 4.43 (q, *J* = 7.1 Hz, 2H), 2.38 (s, 3H), 1.41 (t, *J* = 7.1 Hz, 3H). $^{13}C\{^1H\}$ NMR (151 MHz, $CDCl_3$) *δ* 162.5, 145.1, 137.7, 137.5, 130.1, 128.4, 120.1, 110.3, 61.2, 21.1, 14.5. IR (neat) *ν* 1718, 1510, 1379, 1260, 1174, 1059 cm^{-1}. ESI-MS (*m/z*): 269.3 (35, $[M+K]^+$), 253.3 (88, $[M+Na]^+$), 231.3 (100, $[M+H]^+$). Anal. calcd for $C_{13}H_{14}N_2O_2$ (230.3): C 67.81, H 6.13, N 12.17; found: C 67.83, H 6.14, N 11.96.

3-Ethoxycarbonyl-1-(4-methoxyphenyl)-1*H*-pyrazole[8] (**3s**):

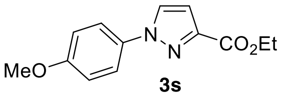

3s

CC (SiO_2, DCM); thick orange oil, 148 mg (60%). ^1H NMR (600 MHz, $CDCl_3$) *δ* 7.83 (d, *J* = 2.4 Hz, 1H, 5-H), 7.65-7.63 (m, 2H), 6.99-6.96 (m, 3H), 4.44 (q, *J* = 7.1 Hz, 2H), 3.85 (s, 3H), 1.42 (t, *J* = 7.1 Hz, 3H). $^{13}C\{^1H\}$ NMR (151 MHz, $CDCl_3$) *δ* 162.5, 159.2, 145.0, 133.5, 128.6, 121.9, 114.6, 110.3, 61.2, 55.7, 14.5. ESI-MS (*m/z*): 247.2 (100, $[M+H]^+$).

3-Acetyl-1-(4-tolyl)-1*H*-pyrazole (**3t**):

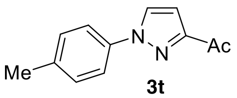

3t

CC (SiO_2, hexanes/DCM 3:2); orange solid, 142 mg (71%); mp 47-48 °C. ^1H NMR (600 MHz, $CDCl_3$) *δ* 7.89 (d, *J* = 2.5 Hz, 1H, 5-H), 7.63-7.61 (m, 2H), 7.30-7.27 (m, 2H), 6.96 (d, *J* = 2.5 Hz, 1H, 4-H), 2.67 (s, 3H), 2.41 (s, 3H). $^{13}C\{^1H\}$ NMR (151 MHz, $CDCl_3$) *δ* 194.3, 152.5, 137.7, 137.6, 130.3, 128.7, 119.8, 108.0, 26.7, 21.1. IR (neat) *ν* 1681, 1521, 1469, 1372, 1260, 1141, 1051 cm^{-1}. ESI-MS (*m/z*): 201.2 (100, $[M+H]^+$). Anal. calcd for $C_{12}H_{12}N_2O$ (200.2): C 71.98, H 6.04, N 13.99; found: C 71.79, H 6.07, N 13.86.

3-Acetyl-1-(4-methoxyphenyl)-1*H*-pyrazole (**3u**):

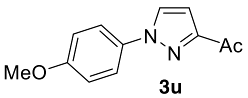

3u

CC (SiO_2, hexanes/DCM 1:4); orange solid, 166 mg (77%); mp 66-68 °C. ^1H NMR (600 MHz, $CDCl_3$) *δ* 7.83 (d, *J* = 2.4 Hz, 1H, 5-H), 7.66-7.63 (m, 2H), 7.02-6.99 (m, 2H), 6.95 (d, *J* = 2.5 Hz, 1H, 4-H), 3.86 (s, 3H), 2.66 (s, 3H). $^{13}C\{^1H\}$ NMR (151 MHz, $CDCl_3$) *δ* 194.3, 159.2, 152.4, 133.6, 128.8, 121.6, 114.8, 107.9, 55.8, 26.7. IR (neat) *ν* 1674, 1521, 1469, 1435, 1372, 1245, 1178, 1137, 1074, 1033

cm^{-1}. ESI-MS (*m/z*): 239.2 (14, $[M+Na]^+$), 217.2 (100, $[M+H]^+$). Anal. calcd for $C_{12}H_{12}N_2O_2$ (216.2): C 66.65, H 5.59, N 12.96; found: C 66.58, H 5.66, N 12.97.

3-Acetyl-1-(4-chlorophenyl)-1*H*-pyrazole (**3v**):

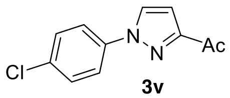

3v

CC (SiO_2, hexanes/DCM 2:3); brown solid, 150 mg (68%); mp 97-99 °C. ^1H NMR (600 MHz, $CDCl_3$) δ 7.90 (d, *J* = 2.5 Hz, 1H, 5-H), 7.71-7.69 (m, 2H), 7.48-7.46 (m, 2H), 6.98 (d, *J* = 2.5 Hz, 1H, 4-H), 2.66 (s, 3H). $^{13}C\{^1H\}$ NMR (151 MHz, $CDCl_3$) δ 194.0, 152.9, 138.4, 133.4, 129.9, 128.7, 121.0, 108.5, 26.7. IR (neat) *v* 1677, 1495, 1375, 1349, 1260, 1234, 1141, 1096, 1062 cm^{-1}. ESI-MS (*m/z*): 223.1 (32, $[M\{^{37}Cl\}+H]^+$), 221.1 (100, $[M\{^{35}Cl\}+H]^+$). Anal. calcd for $C_{11}H_9ClN_2O$ (220.7): C 59.88, H 4.11, N 12.70; found: C 60.06, H 4.00, N 12.73.

3-Acetyl-1-(4-ethoxycarbonylphenyl)-1*H*-pyrazole (**3w**):

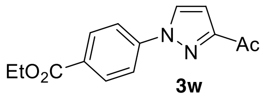

3w

CC (SiO_2, hexanes/EtOAc 4:1); orange solid, 181 mg (70%); mp 123-124 °C. ^1H NMR (600 MHz, $CDCl_3$) δ 8.19-8.17 (m, 2H), 8.01 (d, *J* = 2.6 Hz, 1H, 5-H), 7.85-7.83 (m, 2H), 7.00 (d, *J* = 2.6 Hz, 1H, 4-H), 4.42 (q, *J* = 7.1 Hz, 2H), 2.68 (s, 3H), 1.43 (t, *J* = 7.1 Hz, 3H). $^{13}C\{^1H\}$ NMR (151 MHz, $CDCl_3$) δ 194.0, 165.8, 153.3, 142.9, 131.4, 129.5, 128.8, 119.1, 108.7, 61.4, 26.7, 14.5. IR (neat) *v* 1703, 1681, 1607, 1469, 1379, 1349, 1260, 1178, 1126, 1111, 1059, 1018 cm^{-1}. ESI-MS (*m/z*): 281.2 (42, $[M+Na]^+$), 259.2 (100, $[M+H]^+$). Anal. calcd for $C_{14}H_{14}N_2O_3$ (258.3): C 65.11, H 5.46, N 10.85; found: C 65.25, H 5.35, N 10.91.

3-Acetyl-1-(4-nitrophenyl)-1*H*-pyrazole (**3x**):

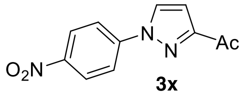

3x

CC (SiO_2, hexanes/DCM 1:4); orange solid, 118 mg (51%); mp >220 °C (decomp.). ^1H NMR (600 MHz, $CDCl_3$) δ 8.40-8.37 (m, 2H), 8.05 (d, *J* = 2.6 Hz, 1H, 5-H), 7.98-7.95 (m, 2H), 7.05 (d, *J* = 2.6 Hz, 1H, 4-H), 2.69 (s, 3H). $^{13}C\{^1H\}$ NMR (151 MHz, $CDCl_3$) δ 193.7, 153.8, 146.5, 144.1, 129.0, 125.6, 119.6, 109.4, 26.7. IR (neat) *v* 1689, 1595, 1510, 1461, 1382, 1334, 1300, 1260, 1141, 1111, 1055 cm^{-1}. ESI-MS (*m/z*): 254.1 (100, $[M+Na]^+$), 232.2 (19, $[M+H]^+$). Anal. calcd for $C_{11}H_9N_3O_3$ (231.2): C 57.14, H 3.92, N 18.17; found: C 57.18, H 3.89, N 18.04.

3-Acetyl-1-(2,4,6-trimethylphenyl)-1*H*-pyrazole (**3y**):

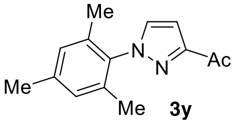

3y

CC (SiO_2, hexanes/EtOAc 4:1); orange solid, 191 mg (84%); mp 64-65 °C. ^{1}H NMR (600 MHz, $CDCl_3$) δ 7.44 (d, J = 2.4 Hz, 1H, 5-H), 6.97 (s_{br}, 2H), 6.96 (d, J = 2.4 Hz, 1H, 4-H), 2.61 (s, 3H), 2.34 (2, 3H), 1.99 (s, 6H). $^{13}C\{^{1}H\}$ NMR (151 MHz, $CDCl_3$) δ 194.4, 152.4, 139.6, 136.6, 135.6, 133.1, 129.2, 106.7, 26.7, 21.2, 17.3. IR (neat) ν 1685, 1469, 1353, 1245, 1126, 1044 cm^{-1}. ESI-MS (*m/z*): 229.3 (100, $[M+H]^+$). Anal. calcd for $C_{14}H_{16}N_2O$ (228.3): C 73.66, H 7.06, N 12.27; found: C 73.49, H 7.05, N 12.00.

Preparation of 1,3,4-thiadiazines 6o and 6x: Following the general protocol for synthesis of pyrazoles **3**, to a solution of hydrazonoyl halide **2o** or **2x** (1.0 mmol) and 2,5-dihydroxy-1,4-dithiane-2,5-diol (**4**, 0.55 mmol, 84 mg) in dry DCM (10 mL) was added dropwise triethylamine (10 mmol, 1.4 mL) and the resulting mixture was stirred at room temperature for 4 h. After a solution of *p*-toluenesulfonic chloride (2.5 mmol, 477 mg) in DCM (5 mL) was added dropwise, the mixture was stirred overnight at room temperature. Water (15 mL) was added, the mixture was extracted with DCM (3 × 10 mL), the combined organic layers were dried over anhydrous Na_2SO_4, filtered, and the solvents were removed under reduced pressure. The crude product **6** was purified by preparative thin layer chromatography (PTLC) using silica gel as stationary phase.

4-(4-Nitrophenyl)-2-trifluoromethyl-4*H*-1,3,4-thiadiazine (**6o**):

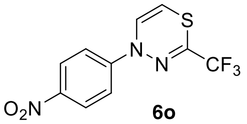

6o

PTLC (SiO_2, hexanes/DCM 1:1); pale yellow solid, 95 mg (34%); mp 88-92 °C (decomp.). ^{1}H NMR (600 MHz, $CDCl_3$) δ 8.29-8.25 (m, 2H), 7.42-7.39 (m, 2H), 6.55 and 5.42 ($2d_{br}$, $J \approx 5.7$ Hz, 1H each, 5-H and 6-H, respectively). $^{13}C\{^{1}H\}$ NMR (151 MHz, $CDCl_3$) δ 148.7, 143.7, 135.2 (q, $^{2}J_{C\text{-}F}$ = 38.9 Hz, C-2), 129.1 (C-5), 125.7, 119.2 (q, $^{1}J_{C\text{-}F}$ = 275.1 Hz, CF_3), 114.7, 99.8 (C-6). ^{19}F NMR (565 MHz, $CDCl_3$) δ –66.89 (s_{br}, CF_3). IR (neat) ν 1587, 1517, 1338, 1264, 1178, 1137, 1111, 977 cm^{-1}. HRMS (ESI-TOF) *m/z*: $[M+H]^+$ calcd for $C_{10}H_7F_3N_3O_2S$ 290.0209, found 290.0211.

2-Acetyl-4-(4-nitrophenyl)-4*H*-1,3,4-thiadiazine (**6x**):

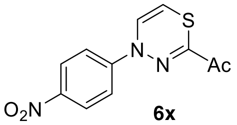

6x

PTLC (SiO_2, hexanes/DCM 1:1); brown solid, 145 mg (55%); mp 148-153 °C (decomp.). ^{1}H NMR (600 MHz, $CDCl_3$) δ 8.28-8.26 (m, 2H), 7.40-7.26 (m, 2H), 6.43 and 5.43 (2d, J = 6.4 Hz, 1H each, 5-H and 6-H, respectively), 2.60 (s, 3H). $^{13}C\{^{1}H\}$ NMR (151 MHz, $CDCl_3$) δ 192.2, 148.4, 143.5,

143.4, 126.2, 125.7, 114.3, 103.4, 26.1. IR (neat) *ν* 1689, 1588, 1558, 1498, 1320, 1245, 1208, 1182, 1096, 1032, 995 cm^{-1}. HRMS (ESI-TOF) *m/z*: $[M+H]^+$ calcd for $C_{11}H_{10}N_3O_3S$ 264.0445, found 264.0443.

Crystals of **6x** suitable for X-ray measurements were obtained from hexane solution by slow evaporation of the solvent.

Synthesis of 5-iodo-3-trifluoromethylpyrazoles 7c and 7m

5-Iodo-1-(4-methoxyphenyl)-3-trifluoromethyl-1*H*-pyrazole (**7c**):

Lithiated pyrazole was generated under inert atmosphere (dry argon) by treatment a solution of pyrazole **3c** (500 mg, 2.07 mmol) in dry THF (20 mL) with *n*-BuLi (2.5M in hexane, 0.83 mL, 2.07 mmol) at –78 °C, under vigorous stirring. After 30 min, a solution of iodine (578 mg, 2.28 mmol) in THF (4 mL) was added and the mixture was allowed to reach room temperature. Then, sat. aq. NH_4Cl (5 mL) was added and the resulting mixture was extracted with Et_2O (3 × 20 mL). Combined organic layers were washed with sat. aq. $Na_2S_2O_3$ (10 mL), then with H_2O (2 × 15 mL), dried over anh. Na_2SO_4, filtered, and the solvents were removed in vacuo. Crude product was purified by flash column chromatography (SiO_2, hexane/EtOAc 9:1) to give iodopyrazole **7c** (693 mg, 91%) as a colorless solid.

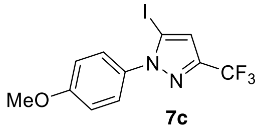

7c

Mp 83-85 °C. ^{1}H NMR (600 MHz, $CDCl_3$) *δ* 7.42-7.40 (m, 2H), 7.01-6.98 (m, 2H), 6.71 (s_{br}, 1H, 4-H), 3.88 (s, 3H). ^{13}C{^{1}H} NMR (151 MHz, $CDCl_3$) *δ* 160.5, 145.4 (q, $^2J_{C\text{-}F}$ = 38.9 Hz, C-3), 132.6, 128.0, 120.8 (q, $^1J_{C\text{-}F}$ = 269.3 Hz, CF_3), 115.0 (q, $^3J_{C\text{-}F}$ = 2.0 Hz, C-4), 114.3, 83.7 (C-5), 55.8. ^{19}F NMR (565 MHz, $CDCl_3$) *δ* –62.52 (s, CF_3). IR (neat) *ν* 1509, 1372, 1230, 1174, 1122, 1098, 1021, 980 cm^{-1}. ESI-MS (*m/z*): 369.1 (100, $[M+H]^+$). Anal. calcd for $C_{11}H_8F_3IN_2O$ (368.1): C 35.89, H 2.19, N 7.61; found: C 36.03, H 2.38, N 7.56.

4-(5'-Iodo-3'-trifluoromethyl-1'*H*-pyrazol-1'-yl)benzenesulfonamide (**7m**):

Following a modified literature protocol,[9] to a solution of pyrazole **3m** (291 mg, 1.0 mmol) in dry THF (20 mL), TMEDA (349 mg, 0.45 mL, 3.0 mmol) followed by *n*-BuLi (2.5M in hexane, 1.2 mL, 3.0 mmol) were added dropwise at –78 °C under vigorous stirring. After 60 min, a solution of excess iodine (762 mg, 3.0 mmol) in dry THF (5 mL) was added and the resulting mixture was allowed to reach room temperature. After 2 h, saturated aqueous solution of NH_4Cl (10 mL) was added, the resulting mixture was extracted with Et_2O (3 × 15 mL), combined organic layers were washed with 10% aqueous solution of $Na_2S_2O_3$ (20 mL), then with H_2O (10 mL), and dried over anhydrous Na_2SO_4 and filtered. After solvents were removed under reduced pressure, the crude product was purified by column chromatography (SiO_2, petroleum ether/EtOAc 3:2) to give iodopyrazole **7m** (250 mg, 60%) as a colorless solid.

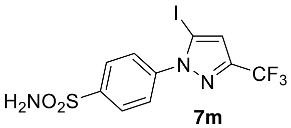

7m Mp 168-170 °C. ^{1}H NMR (600 MHz, DMSO-d_6) δ 8.03-8.01 (m, 2H), 7.85-7.82 (m, 2H), 7.59 (s_{br}, 2H), 7.35 (s_{br}, 1H, 4'-H). ^{13}C{^{1}H} NMR (151 MHz, DMSO-d_6) δ 144.9, 144.3 (q, $^2J_{C\text{-}F}$ = 38.0 Hz, C-3'), 141.4, 127.2, 126.8, 120.8 (q, $^1J_{C\text{-}F}$ = 269.2 Hz, CF_3), 115.6 (q, $^3J_{C\text{-}F}$ = 1.3 Hz, C-4'), 89.0 (C-5'). ^{19}F NMR (565 MHz, DMSO-d_6) δ –61.06 (s, CF_3). IR (neat) ν 3325, 3194, 1338, 1230, 1137, 1092 cm^{-1}. ESI-MS (m/z): 440.2 (100, $[M+Na]^+$), 418.3 (54, $[M+H]^+$). Anal. calcd for $C_{10}H_7F_3IN_3O_2S$ (417.1): C 28.79, H 1.69, N 10.07, S 7.69; found: C 28.74, H 1.96, N 10.01, S 7.74.

Synthesis of 1,5-diaryl-3-trifluoromethylpyrazoles 8-10: A solution of 5-iodopyrazole **7** (0.25 mmol), K_2CO_3 (1.09 mmol) and arylboronic acid (0.32 mmol) in THF/H_2O (4:1 mixture, 10 mL) was degassed by a repeated procedure of freeze-pump-thaw, and $Pd(PPh_3)_4$ (95 mg, 30 mol% with respect to iodopyrazole) was added. The mixture was refluxed for 2 days (oil bath), cooled to room temperature, H_2O was added and the resulting was extracted with DCM (3 × 5 mL). Combined organic layers were dried over anh. Na_2SO_4, filtered, and the solvents were removed in vacuo. Crude product was purified by standard column chromatography (CC).

5-(4-Chlorophenyl)-1-(4-methoxyphenyl)-3-trifluoromethyl-1*H*-pyrazole[10] (**8**, SC-560):

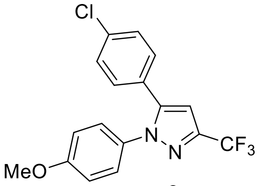

8 CC (SiO_2, hexanes/EtOAc 4:1); yellow oil, 76 mg (86%);. ^{1}H NMR (600 MHz, $CDCl_3$) δ 7.31-7.29 (m, 2H), 7.22-7.20 (m, 2H), 7.16-7.14 (m, 2H), 6.89-6.87 (m, 2H), 6.73 (s_{br}, 1H, 4-H), 3.83 (s, 3H). ^{13}C{^{1}H} NMR (151 MHz, $CDCl_3$) δ 159.8, 143.5, 143.1 (q, $^2J_{C\text{-}F}$ = 38.4 Hz), 135.2, 132.2, 130.1, 129.1, 127.8, 127.0, 121.4 (q, $^1J_{C\text{-}F}$ = 268.9 Hz, CF_3), 114.5, 105.4 (q, $^3J_{C\text{-}F}$ = 2.1 Hz), 55.7. ^{19}F NMR (565 MHz, $CDCl_3$) δ –62.18 (s, CF_3). ESI-MS (m/z): 355.2 (33, $[M\{^{37}Cl\}+H]^+$), 353.3 (100, $[M\{^{35}Cl\}+H]^+$).

4-[5'-(4''-Methylphenyl)-3'-trifluoromethyl-1'*H*-pyrazol-1'-yl)benzenesulfonamide[11] (**9**):

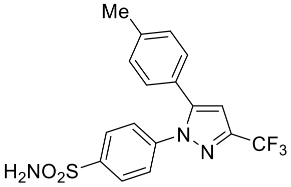

9 CC (SiO_2, hexanes/EtOAc 1:1); colorless solid, 81 mg (85%); mp 159-161 °C. ^{1}H NMR (600 MHz, DMSO-d_6) δ 7.87 (d_{br}, $J \approx$ 8.5 Hz, 2H), 7.55-7.52 (m, 4H), 7.23-7.19 (m, 5H), 2.31 (s, 3H). ^{13}C{^{1}H} NMR (151 MHz, DMSO-d_6) δ 145.3, 144.0, 142.2 (q, $^2J_{C\text{-}F}$ = 37.5 Hz, C-3'), 141.1, 139.1, 129.5,

128.8, 126.8, 126.0, 125.4, 121.3 (q, $^1J_{C\text{-}F}$ = 268.7 Hz, CF_3), 106.2 (br), 20.8. ^{19}F NMR (565 MHz, DMSO-d_6) δ –60.85 (s, CF_3). ESI-MS (*m/z*): 382.3 (100, $[M+H]^+$).

4-[5′-(4′′-Fluorophenyl)-3′-trifluoromethyl-1′*H*-pyrazol-1′-yl)benzenesulfonamide[11] (**10**):

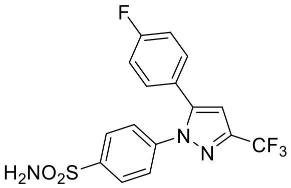

10 CC (SiO_2, hexanes/EtOAc 3:2); colorless solid, 89 mg (93%); mp 161-163 °C. ^{1}H NMR (600 MHz, $CDCl_3$) δ 7.94-7.91 (m, 2H), 7.48-7.45 (m, 2H), 7.24-7.21 (m, 2H), 7.11-7.07 (m, 2H), 6.76 (s_{br}, 1H), 4.91 (s_{br}, 2H). ^{13}C{^{1}H} NMR (151 MHz, $CDCl_3$) δ 163.4 (d, $^1J_{C\text{-}F}$ = 251.3 Hz), 144.4 (q, $^2J_{C\text{-}F}$ = 38.6 Hz, C-3′), 144.2, 142.4, 141.7, 131.0 (d, $^3J_{C\text{-}F}$ = 8.4 Hz), 127.8, 125.7, 124.9 (d, $^4J_{C\text{-}F}$ = 3.6 Hz), 121.1 (q, $^1J_{C\text{-}F}$ = 269.1 Hz, CF_3), 116.5 (d, $^2J_{C\text{-}F}$ = 22.0 Hz), 106.8 (q, $^3J_{C\text{-}F}$ = 1.7 Hz, C-4′). ^{19}F NMR (565 MHz, $CDCl_3$) δ –62.49 (s, CF_3), –110.13 (m_c, Ar-F). ESI-MS (*m/z*): 408.2 (61, $[M+Na]^+$), 386.3 (100, $[M+H]^+$).

Synthesis of hydrazonoyl bromide 2m:

A mixture of 4-hydrazinylbenzenesulfonamide hydrochloride (1.12 g, 5.0 mmol), fluoral hydrate (ca. 12.0 mmol), freshly dried and crushed molecular sieves 4Å (2.0 g) in methanol (25 mL) was heated at 80 °C (oil bath) in a closed ampoule for 16 h. The mixture was cooled to room temperature, filtered through Celite®, and the plug of Celite was washed with EtOAc (3 × 50 mL). The filtrate was washed with water (100 mL), then with 5% aq. $NaHCO_3$ (30 mL), the organic layer was dried over Na_2SO_4, filtered, and the solvents were removed in vacuo. The obtained crude product was flash chromatographed (SiO_2, petroleum ether/EtOAc 2:3) to give the respective hydrazone **11** (1.27 g, 95%) as pale yellow solid.

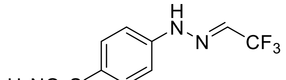

11 Mp 145-147 °C. ^{1}H NMR (600 MHz, DMSO-d_6) δ 11.42 (s_{br}, 1H), 7.73-7.71 (m, 2H), 7.42 (q, $J_{H\text{-}F}$ = 4.3 Hz, 1H), 7.18 (s_{br}, 2H), 7.15-7.12 (m, 2H). ^{13}C{^{1}H} NMR (151 MHz, DMSO-d_6) δ 146.1, 136.1, 127.6, 124.2 (q, $^2J_{C\text{-}F}$ = 37.3 Hz, =*C*-CF_3), 121.5 (q, $^1J_{C\text{-}F}$ = 269.0 Hz, CF_3), 112.3. ^{19}F NMR (565 MHz, DMSO-d_6) δ –64.05 (d, $J_{H\text{-}F}$ = 4.3 Hz, CF_3). IR (neat) ν 1595, 1327, 1260, 1141, 1096 cm^{-1}. ESI-MS (*m/z*): 290.2 (100, $[M+Na]^+$), 268.1 (8, $[M+H]^+$). Anal. calcd for $C_8H_8F_3N_3O_2S$ (267.2): C 35.96, H 3.02, N 15.72, S 12.00; found: C 35.97, H 2.94, N 15.78, S 12.25.

A solution of hydrazone **11** obtained in the previous step (534 mg, 2.0 mmol) in dry DMF (15 mL) was cooled to 0 °C, solid NBS (428 mg, 2.4 mmol) was added portion-wise, and the resulting mixture was stirred at room temperature overnight. Then H_2O (20 mL) was added, the mixture was extracted with EtOAc (3 × 30 mL), the organic layer was washed with brine (3 × 10 mL), dried over Na_2SO_4, filtered and the solvents were removed under reduced pressure. Crude product was purified by column chromatography (SiO_2, petroleum ether/EtOAc 2:3) to give bromide **2m** (484 mg, 70%) as a colorless solid.

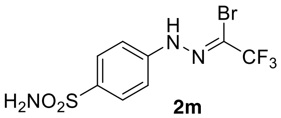
2m

Mp 148-149 °C. ^{1}H NMR (600 MHz, DMSO-d_6) δ 10.82 (s_{br}, 1H), 7.77-7.75 (m, 2H), 7.43-7.40 (m, 2H), 7.24 (s_{br}, 2H). ^{13}C{^{1}H} NMR (151 MHz, DMSO-d_6) δ 145.2, 137.5, 127.4, 118.7 (q, $^1J_{C-F}$ = 270.8 Hz, CF_3), 114.1, 102.5 (q, $^2J_{C-F}$ = 41.8 Hz, =*C*-CF_3). ^{19}F NMR (565 MHz, DMSO-d_6) δ –65.24 (s, CF_3). IR (neat) ν 3273, 1595, 1513, 1323, 1245, 1133, 1089 cm^{-1}. (–)-ESI-MS (*m/z*): 345.8 (100, [M{^{81}Br}–H]$^-$), 343.9 (89, [M{^{79}Br}–H]$^-$). Anal. calcd for $C_8H_7BrF_3N_3O_2S$ (346.1): C 27.76, H 2.04, N 12.14, S 9.26; found: C 27.81, H 2.14, N 11.96, S 9.19.

Synthesis of hydrazonoyl chlorides 2v-2y: A solution of 3-chloropentane-2,4-dione (1.34 g, 10 mmol) in EtOH (40 mL) was placed in an ice bath, and solid NaOH (0.4 g, 10 mmol) was added upon vigorous stirring, followed by portion-wise addition of the freshly prepared arene-diazonium salt [obtained by treatment of aniline derivative (12 mmol) with aq. HCl (6M, 7.0 mL) at 0 °C, followed by dropwise addition of a solution of $NaNO_2$ (0.83 g, 12 mmol) in H_2O (12 mL) at 0 °C for 20 min]. The stirring was continued for 3 h at 0 °C, the crude product was filtered off, washed with H_2O (3 × 30 mL), air dried and either recrystallized from hexanes (**2w** and **2y**) or used in the next step as received (**2v** and **2x**).

1-Chloro-1-[(4-chlorophenyl)hydrazono]-2-propanone (**2v**):

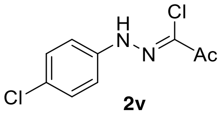
2v

Yellow solid, 1.69 g (73%); mp 171-173 °C. ^{1}H NMR (600 MHz, $CDCl_3$) δ 8.43 (s_{br}, 1H), 7.34-7.31 (m, 2H), 7.18-7.16 (m, 2H), 2.56 (s, 3H). ^{13}C{^{1}H} NMR (151 MHz, $CDCl_3$) δ 188.3, 140.2, 129.8, 128.7, 126.0, 115.8, 25.4. IR (neat) ν 3232, 1674, 1536, 1484, 1219, 1167, 1084, 1018 cm^{-1}. ESI-MS (*m/z*): 255.2 (63, [M{^{37}Cl}+Na]$^+$), 253.1 (100, [M{^{35}Cl}+Na]$^+$). Anal. calcd for $C_9H_8Cl_2N_2O$ (232.1): C 46.78, H 3.49, N 12.12; found: C 46.79, H 3.47, N 12.07.

1-Chloro-1-[(4-ethoxycarbonylphenyl)hydrazono]-2-propanone (**2w**):

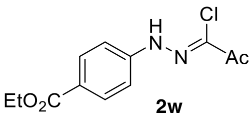
2w

Yellow solid, 2.18 g (81%); mp 167-169 °C (hexanes). ^{1}H NMR (600 MHz, $CDCl_3$) δ 8.55 (s_{br}, 1H), 8.08-8.05 (m, 2H), 7.27-7.24 (m, 2H), 4.38 (t, *J* = 7.1 Hz, 2H), 2.59 (s, 3H), 1.40 (t, *J* = 7.1 Hz, 3H). ^{13}C{^{1}H} NMR (151 MHz, $CDCl_3$) δ 188.3, 166.2, 145.0, 131.7, 127.2, 125.6, 114.0, 61.1, 25.5, 14.5. IR (neat) ν 3243, 1670, 1607, 1547, 1525, 1279, 1226, 1159, 1096, 1018 cm^{-1}. ESI-MS (*m/z*): 271.1 (28, [M{^{37}Cl}+H]$^+$), 269.1 (100, [M{^{35}Cl}+H]$^+$). Anal. calcd for $C_{12}H_{13}ClN_2O_3$ (268.7): C 53.64, H 4.88, N 10.43; found: C 53.75, H 4.78, N 10.52.

1-Chloro-1-[(4-nitrophenyl)hydrazono]-2-propanone (**2x**):

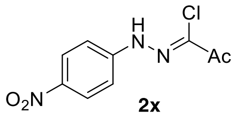

2x

Orange solid, 1.26 g (52%); mp 219-223 °C (decomp). ^{1}H NMR (600 MHz, $CDCl_3$) δ 8.65 (s_{br}, 1H), 8.29-8.27 (m, 2H), 7.33-7.31 (m, 2H), 2.61 (s, 3H). ^{13}C{^{1}H} NMR (151 MHz, $CDCl_3$) δ 188.0, 146.5, 143.4, 128.9, 126.2, 114.3, 25.6. IR (neat) ν 3254, 1681, 1603, 1536, 1506, 1334, 1226, 1163, 1111, 1021 cm^{-1}. ESI-MS (*m/z*): 245.1 (28, [M{^{37}Cl}+H]$^+$), 243.1 (100, [M{^{35}Cl}+H]$^+$). Anal. calcd for $C_9H_8ClN_3O_3$ (241.6): C 44.74, H 3.34, N 17.39; found: C 44.78, H 3.24, N 17.28.

1-Chloro-1-[(2,4,6-trimethylphenyl)hydrazono]-2-propanone (**2y**):

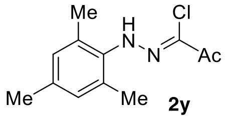

2y

Colorless solid, 1.46 g (61%); mp 80-82 °C (hexanes). ^{1}H NMR (600 MHz, $CDCl_3$) δ 8.13 (s_{br}, 1H), 6.91 (s, 2H), 2.46 (s, 3H), 2.34 (s, 6H), 2.29 (s, 3H). ^{13}C{^{1}H} NMR (151 MHz, $CDCl_3$) δ 188.4, 135.6, 135.4, 130.1, 130.0, 124.1, 25.3, 20.9, 18.8. IR (neat) ν 3288, 1685, 1528, 1443, 1219, 1098, 1018 cm^{-1}. ESI-MS (*m/z*): 241.2 (28, [M{^{37}Cl}+H]$^+$), 239.2 (100, [M{^{35}Cl}+H]$^+$). Anal. calcd for $C_{12}H_{15}ClN_2O$ (238.7): C 60.38, H 6.33, N 11.74; found: C 60.19, H 6.28, N 11.48.

3. Copies of NMR spectra

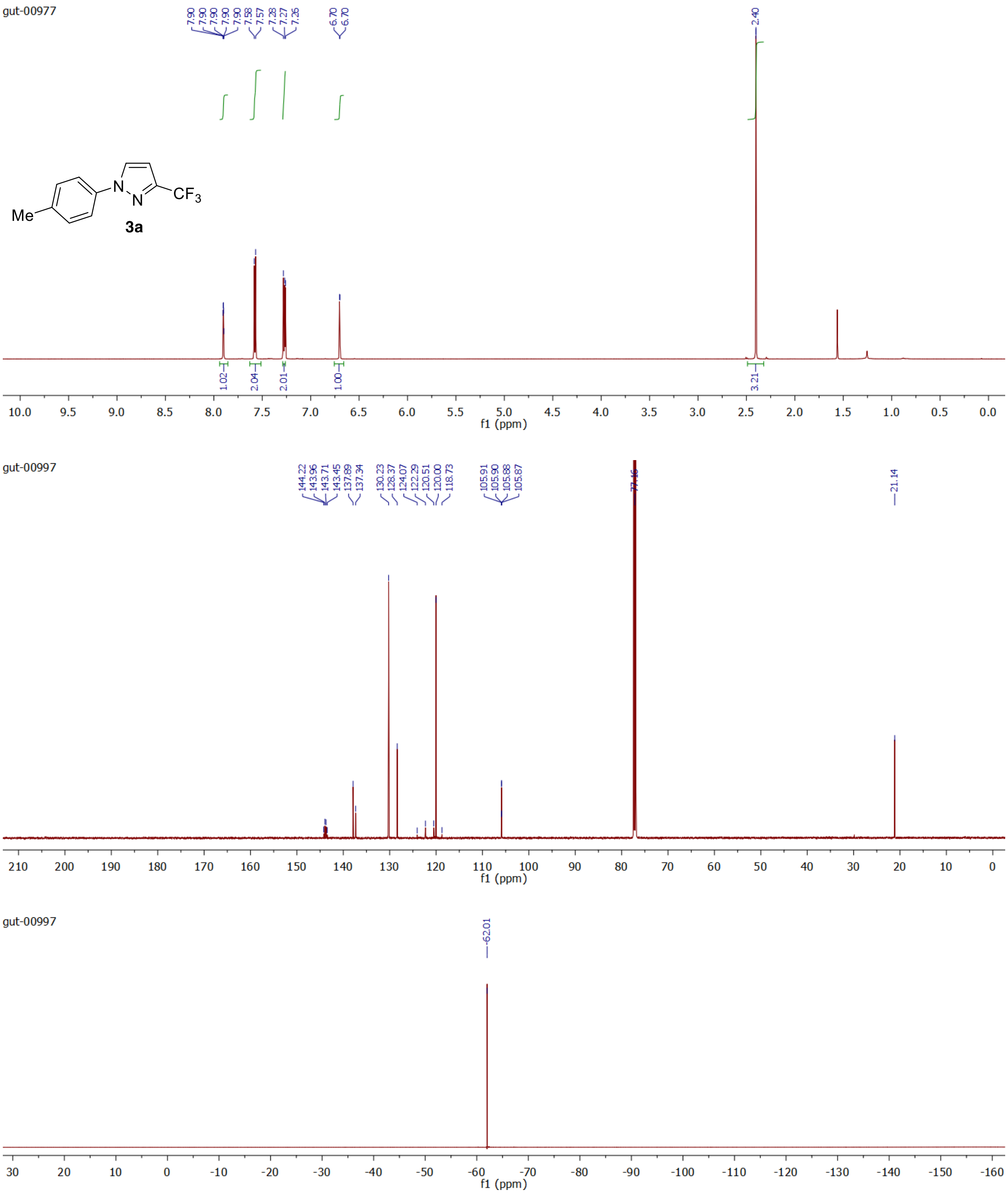

Fig S1. ^{1}H NMR (600 MHz, $CDCl_3$), ^{13}C NMR (151 MHz, $CDCl_3$) and ^{19}F NMR (565 MHz, $CDCl_3$) spectra for compound **3a**.

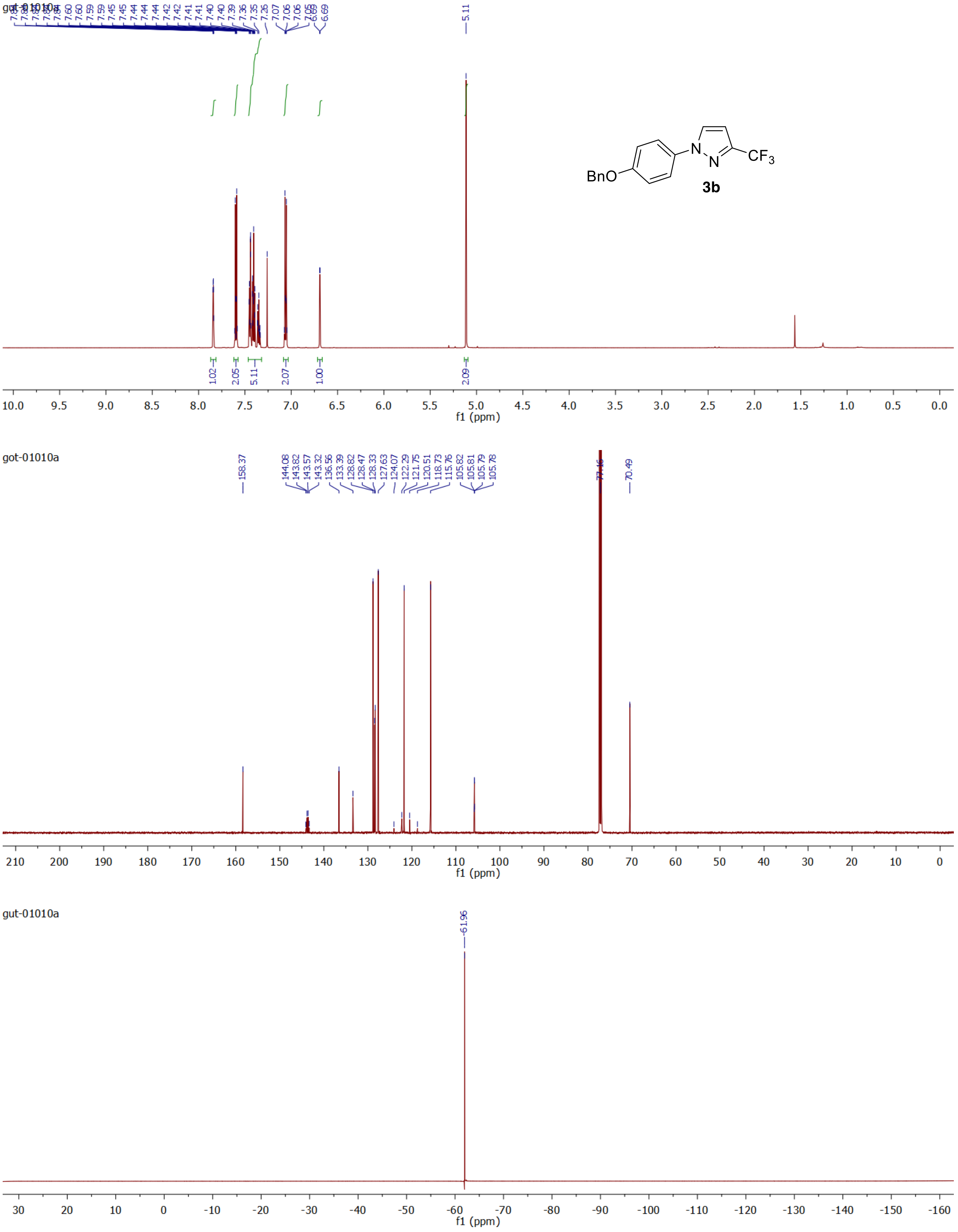

Fig S2. ^{1}H NMR (600 MHz, $CDCl_3$), ^{13}C NMR (151 MHz, $CDCl_3$) and ^{19}F NMR (565 MHz, $CDCl_3$) spectra for compound **3b**.

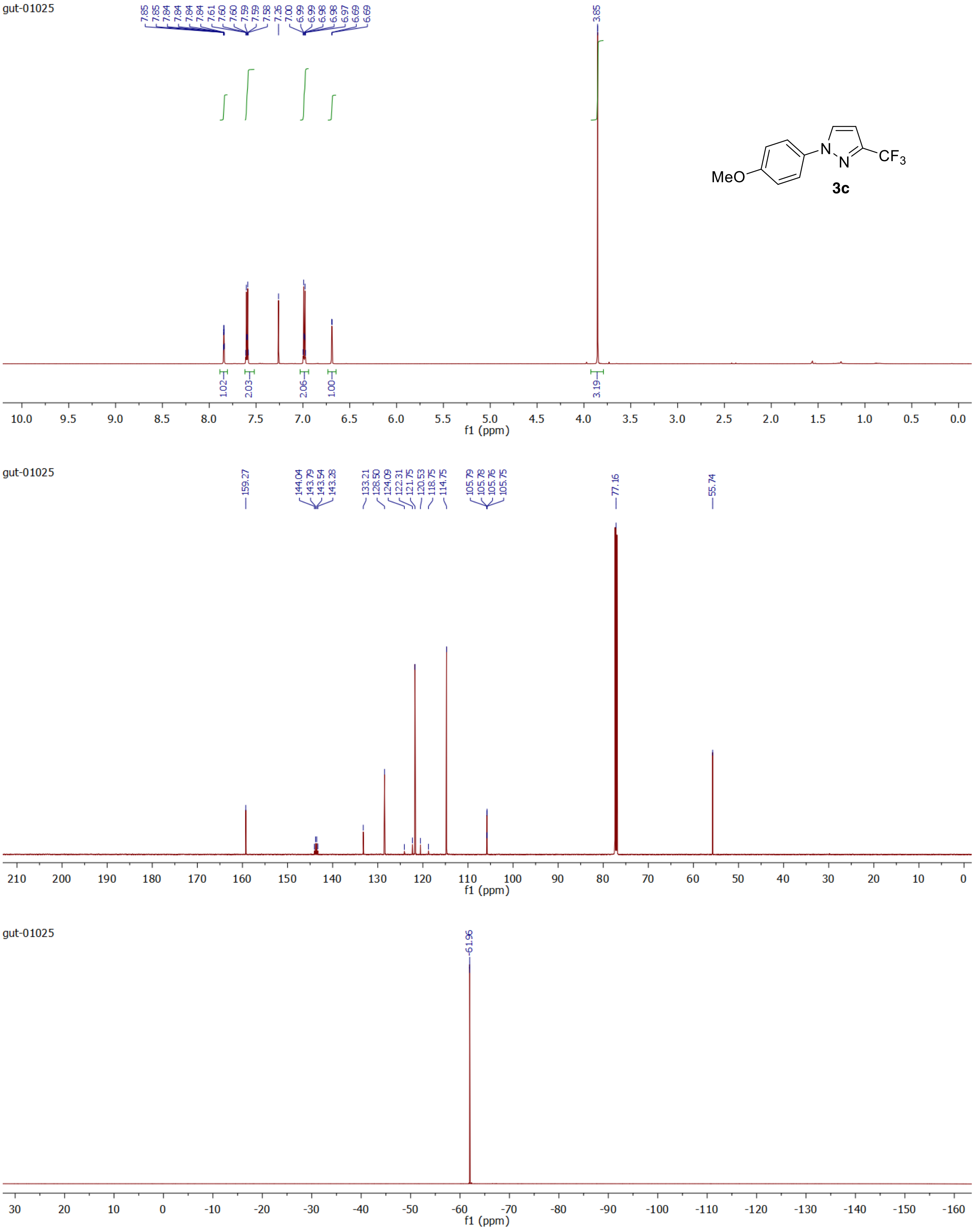

Fig S3. ^{1}H NMR (600 MHz, $CDCl_3$), ^{13}C NMR (151 MHz, $CDCl_3$) and ^{19}F NMR (565 MHz, $CDCl_3$) spectra for compound **3c**.

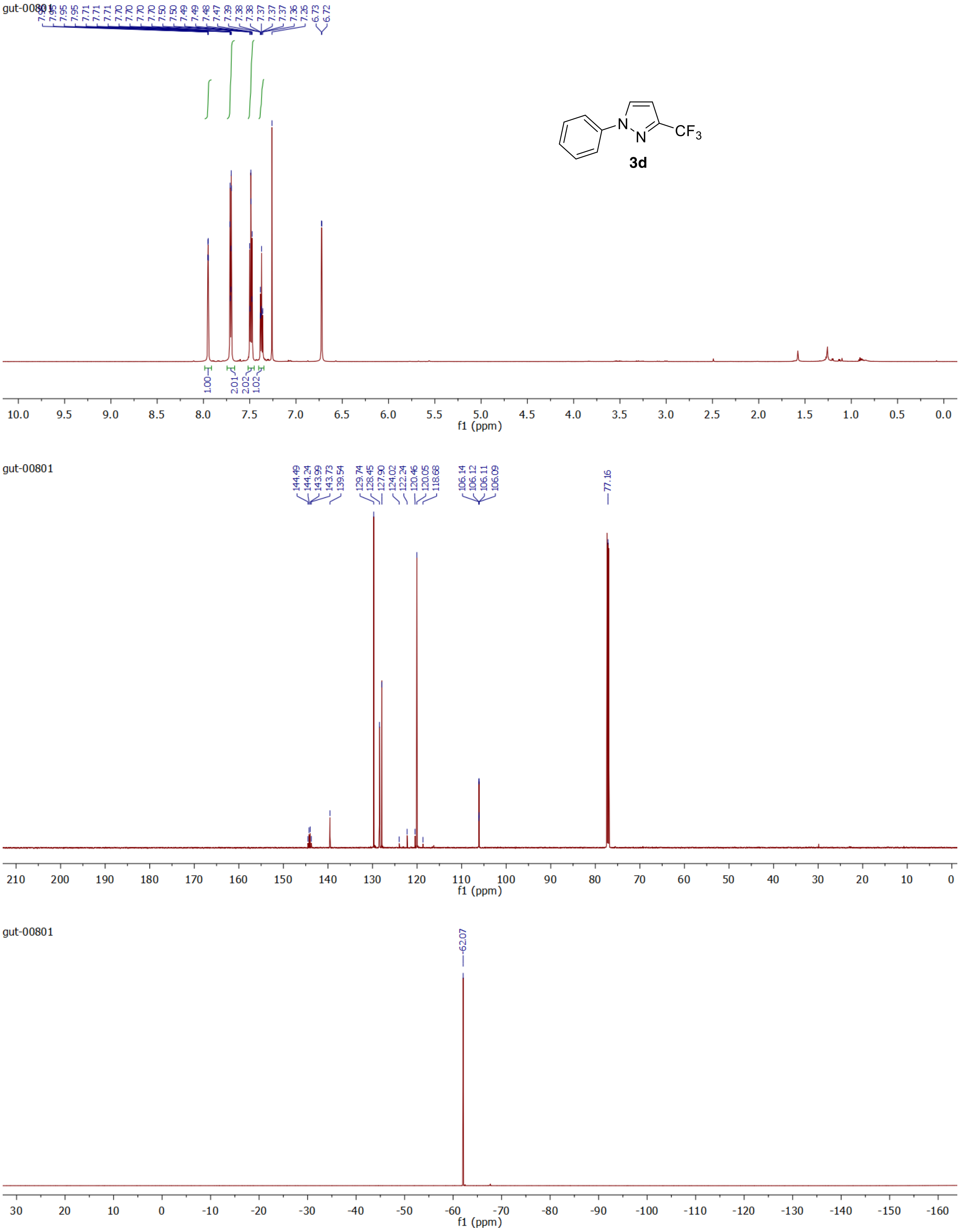

Fig S4. ^{1}H NMR (600 MHz, $CDCl_3$), ^{13}C NMR (151 MHz, $CDCl_3$) and ^{19}F NMR (565 MHz, $CDCl_3$) spectra for compound **3d**.

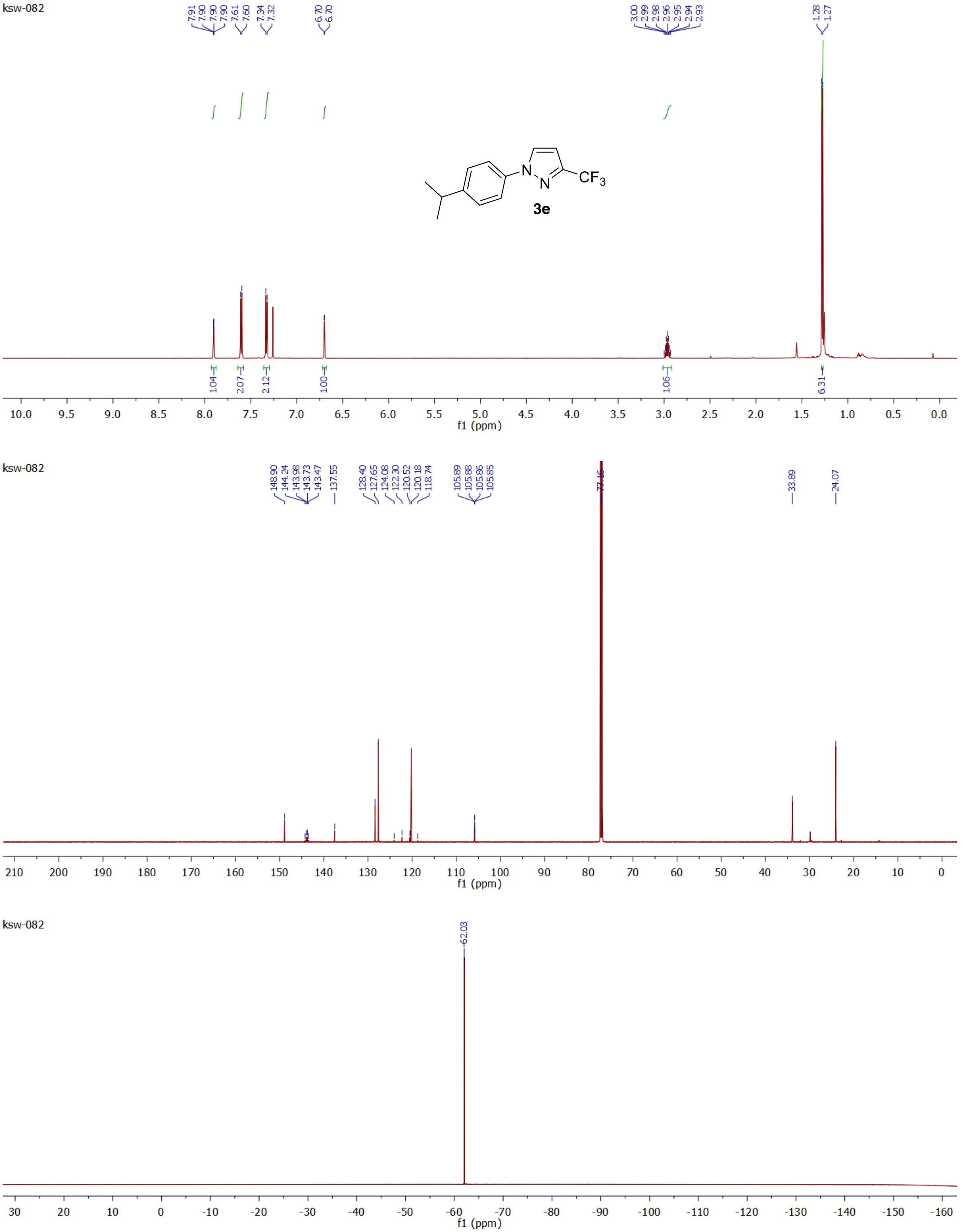

Fig S5. ^{1}H NMR (600 MHz, $CDCl_3$), ^{13}C NMR (151 MHz, $CDCl_3$) and ^{19}F NMR (565 MHz, $CDCl_3$) spectra for compound **3e**.

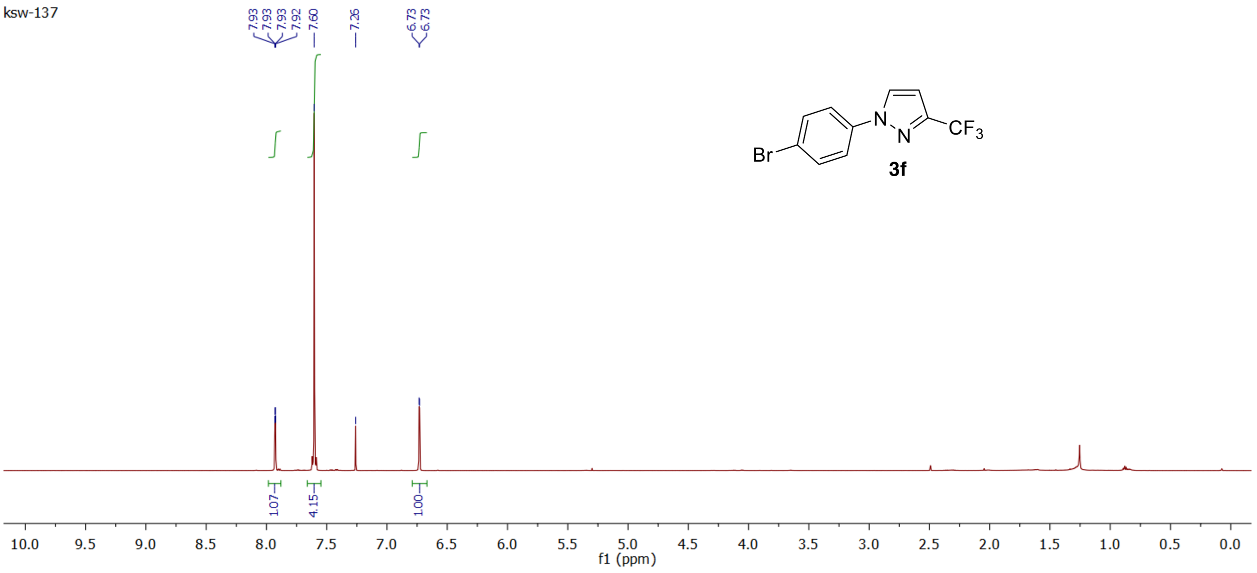

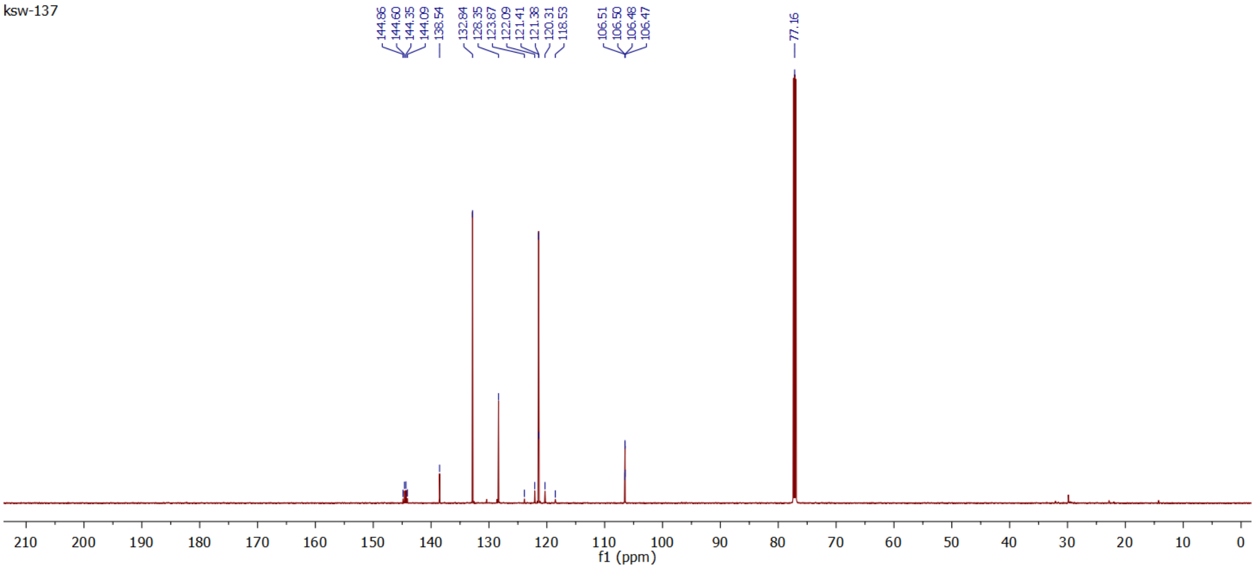

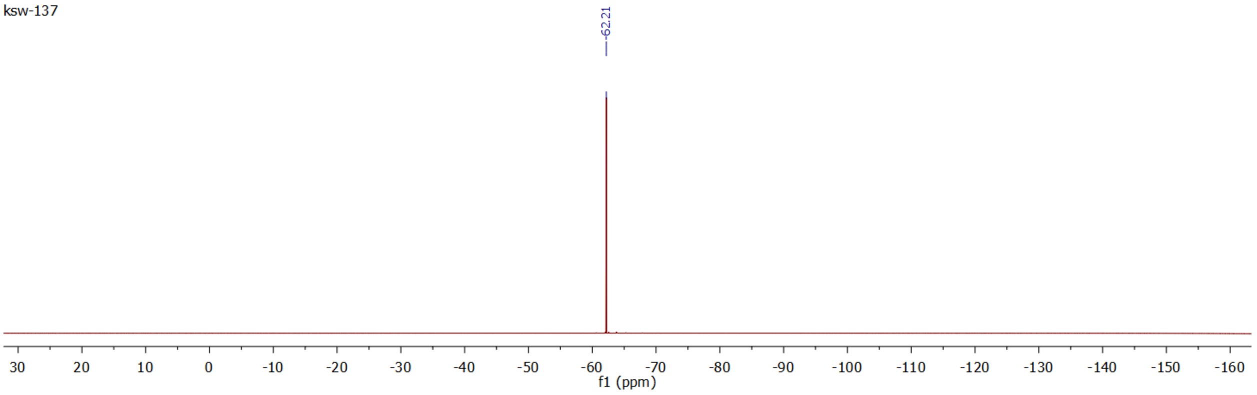

Fig S6. ^{1}H NMR (600 MHz, $CDCl_3$), ^{13}C NMR (151 MHz, $CDCl_3$) and ^{19}F NMR (565 MHz, $CDCl_3$) spectra for compound **3f**.

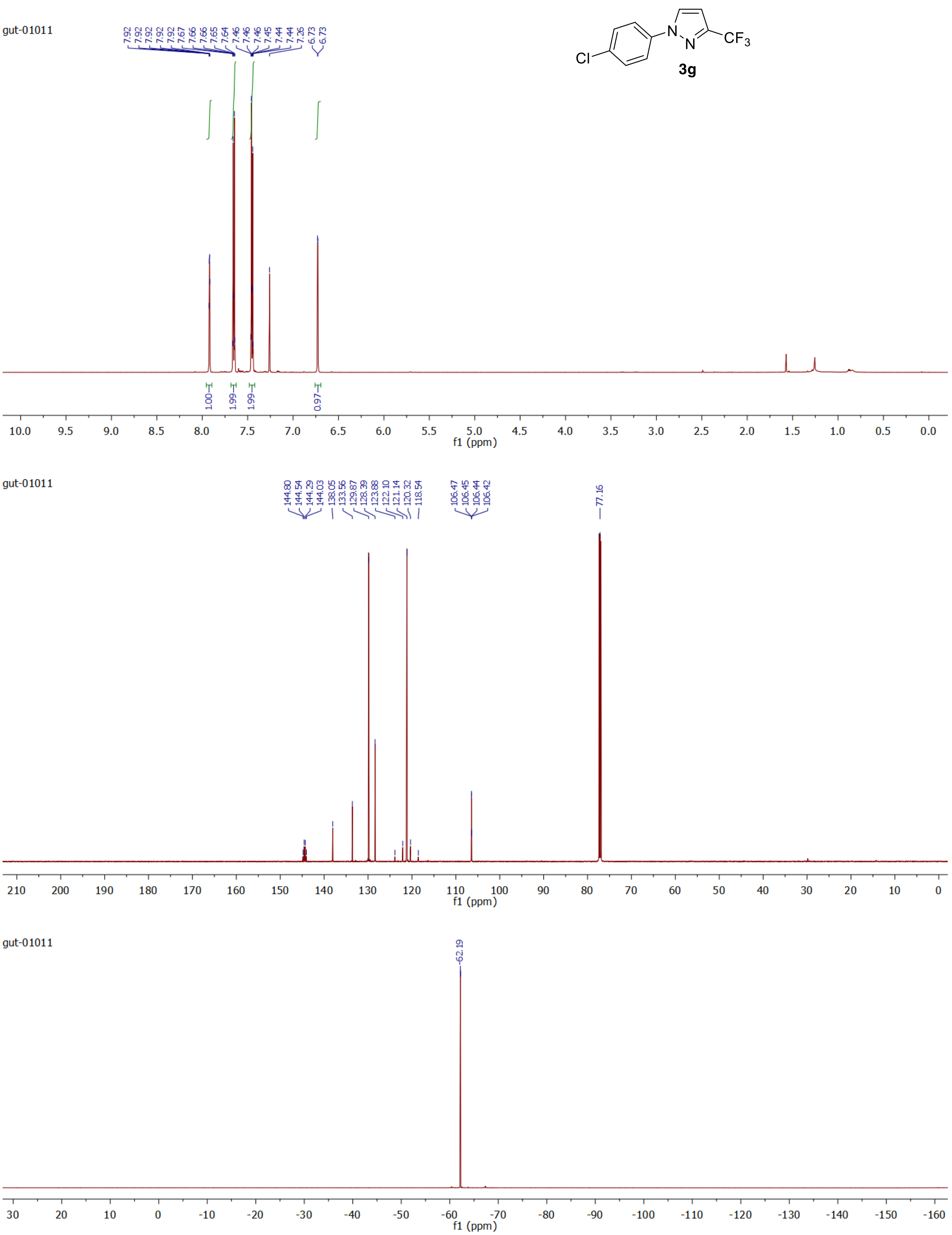

Fig S7. ^{1}H NMR (600 MHz, $CDCl_3$), ^{13}C NMR (151 MHz, $CDCl_3$) and ^{19}F NMR (565 MHz, $CDCl_3$) spectra for compound **3g**.

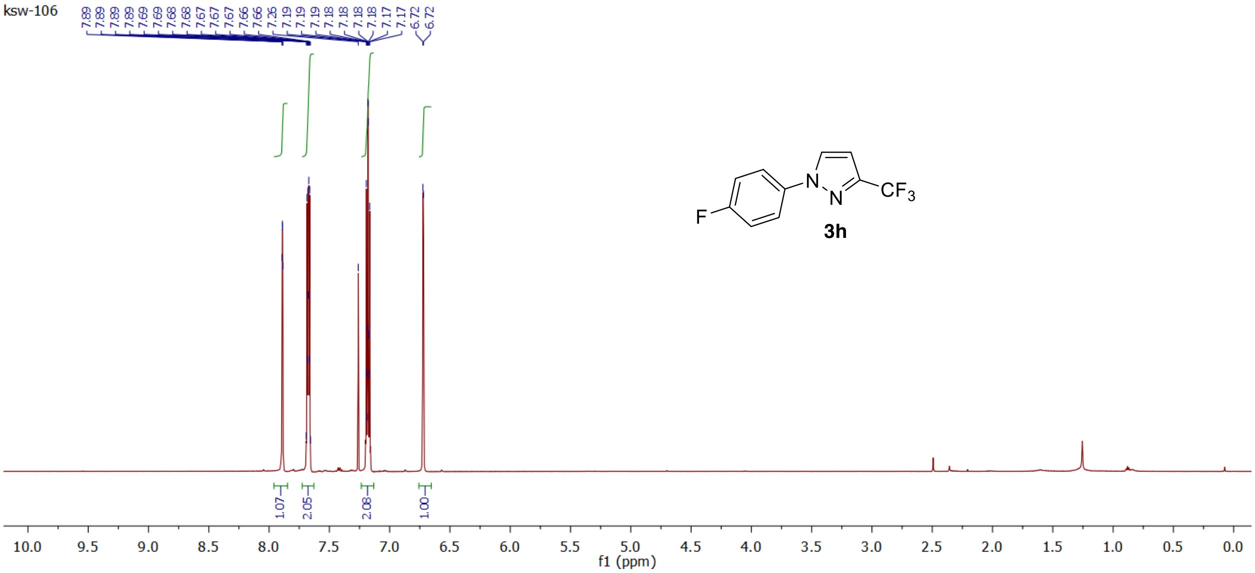

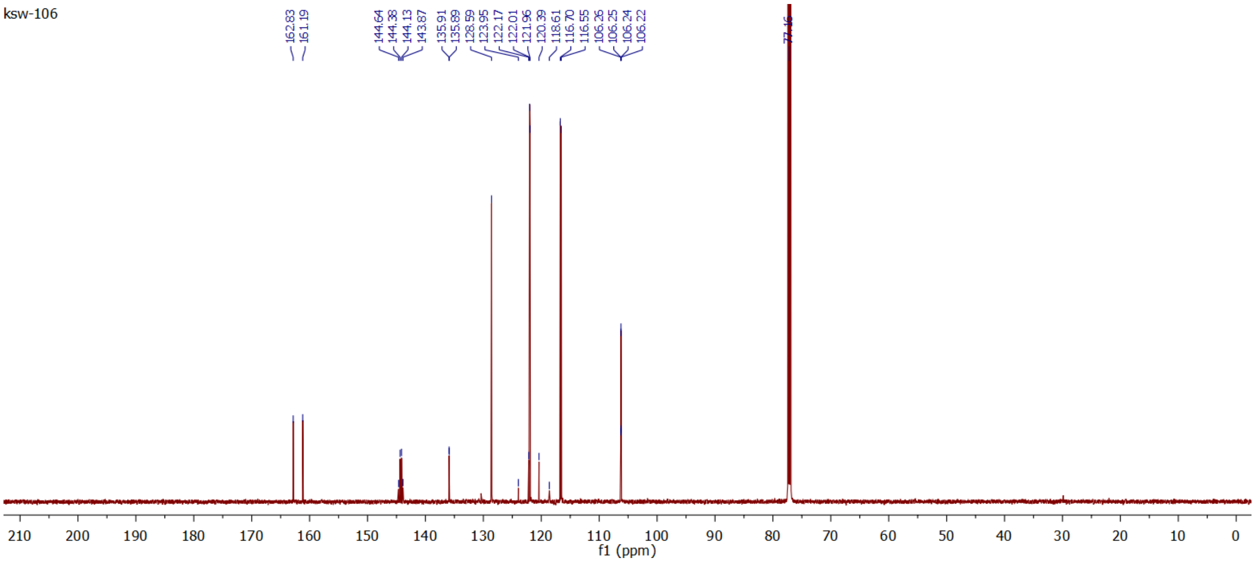

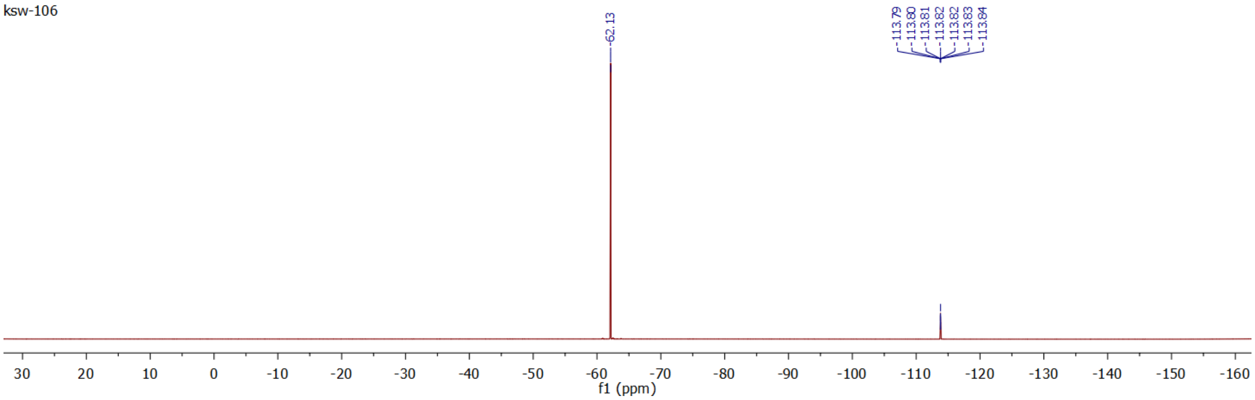

Fig S8. ^{1}H NMR (600 MHz, $CDCl_3$), ^{13}C NMR (151 MHz, $CDCl_3$) and ^{19}F NMR (565 MHz, $CDCl_3$) spectra for compound **3h**.

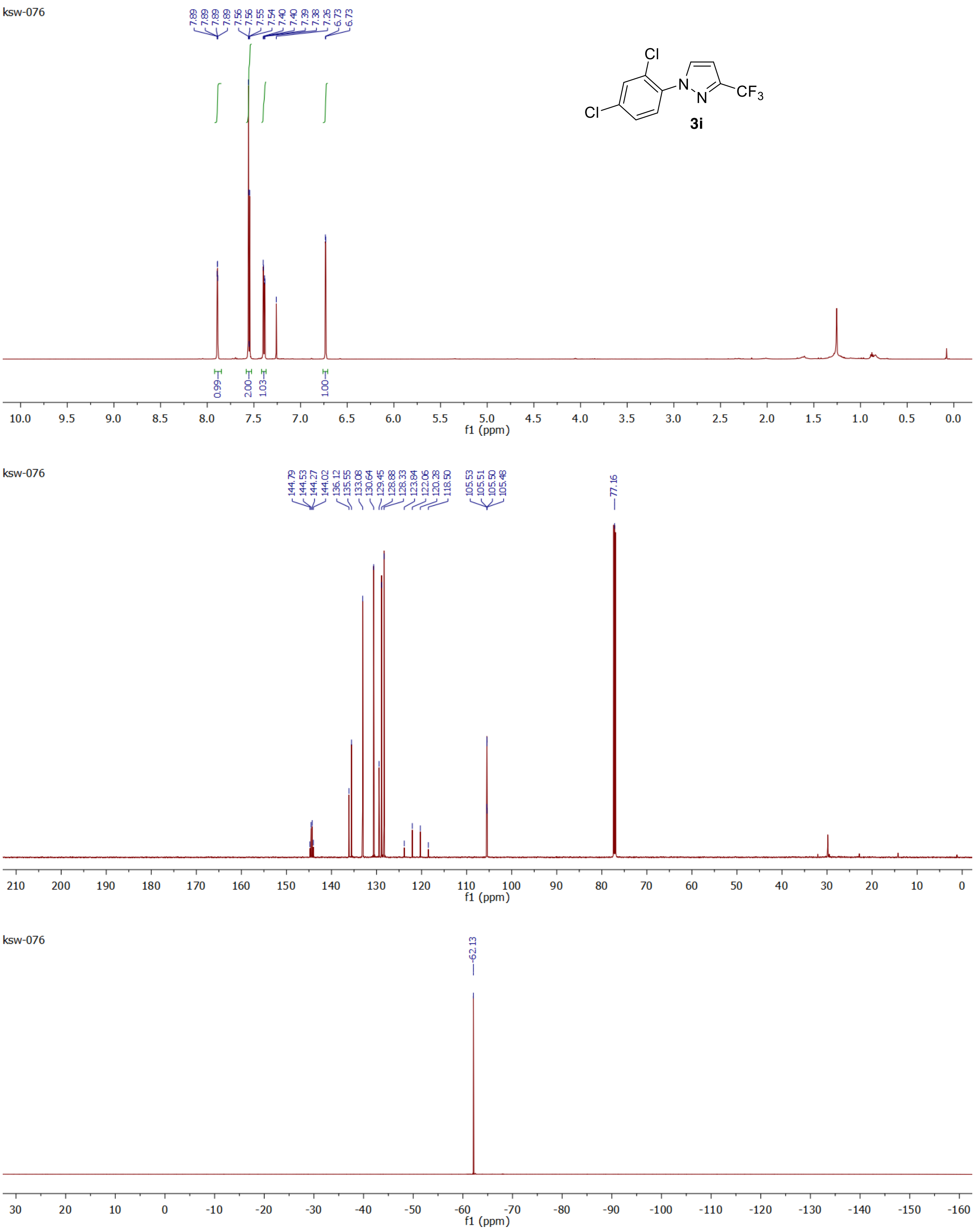

Fig S9. ^{1}H NMR (600 MHz, $CDCl_3$), ^{13}C NMR (151 MHz, $CDCl_3$) and ^{19}F NMR (565 MHz, $CDCl_3$) spectra for compound **3i**.

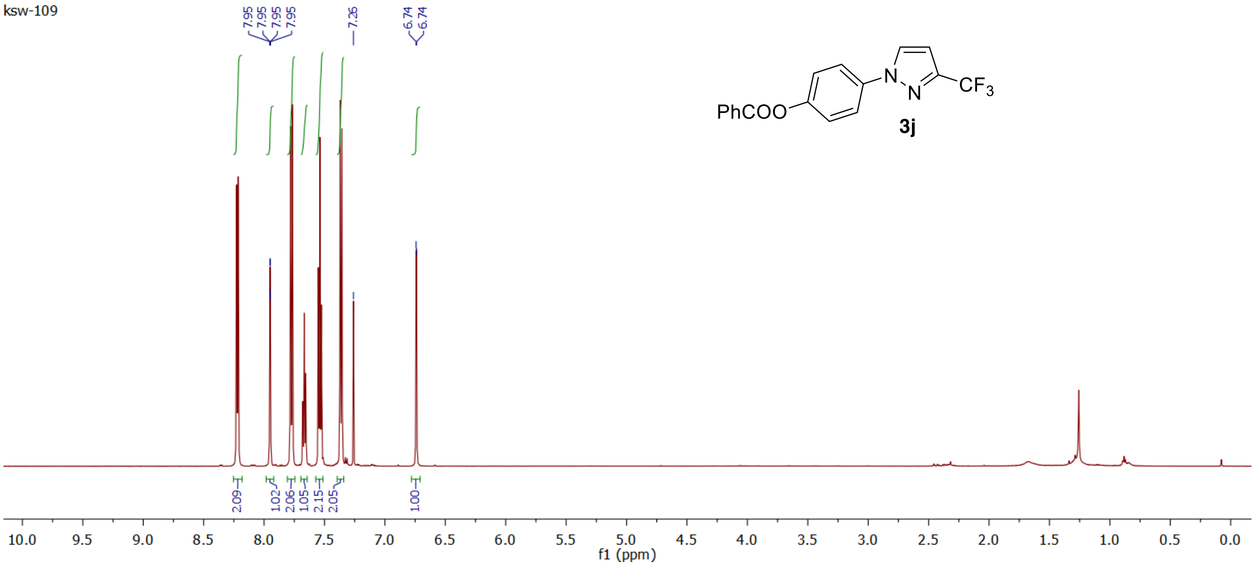

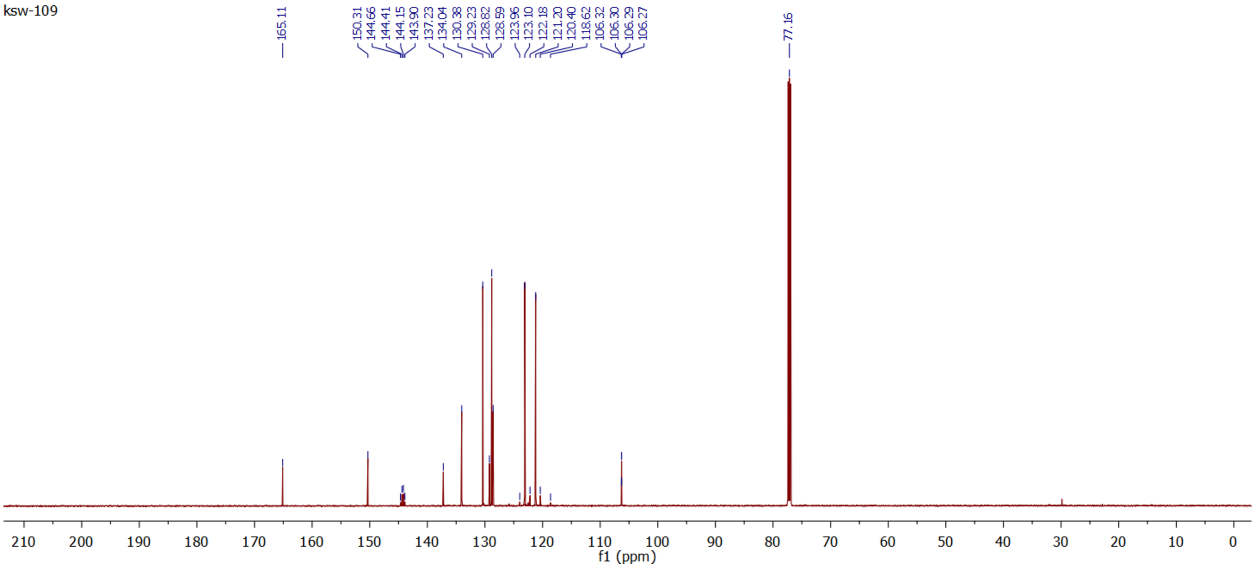

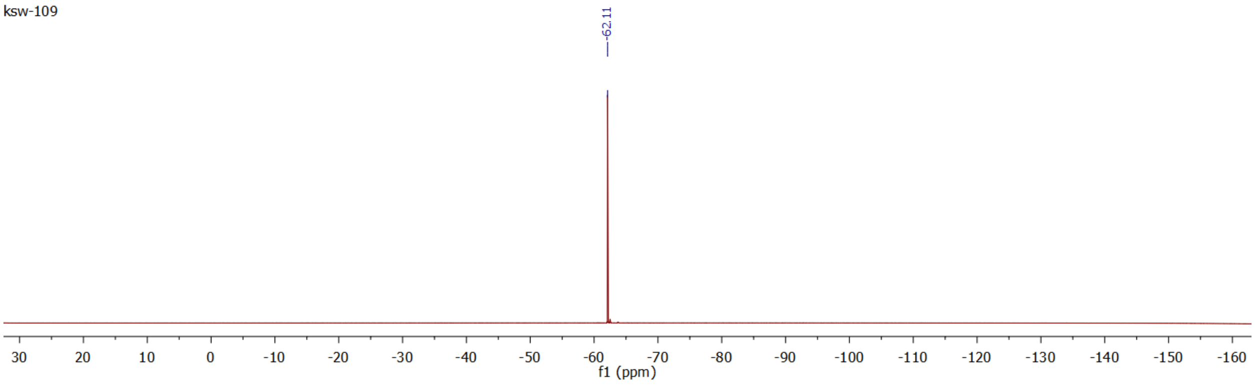

Fig S10. ^{1}H NMR (600 MHz, $CDCl_3$), ^{13}C NMR (151 MHz, $CDCl_3$) and ^{19}F NMR (565 MHz, $CDCl_3$) spectra for compound **3j**.

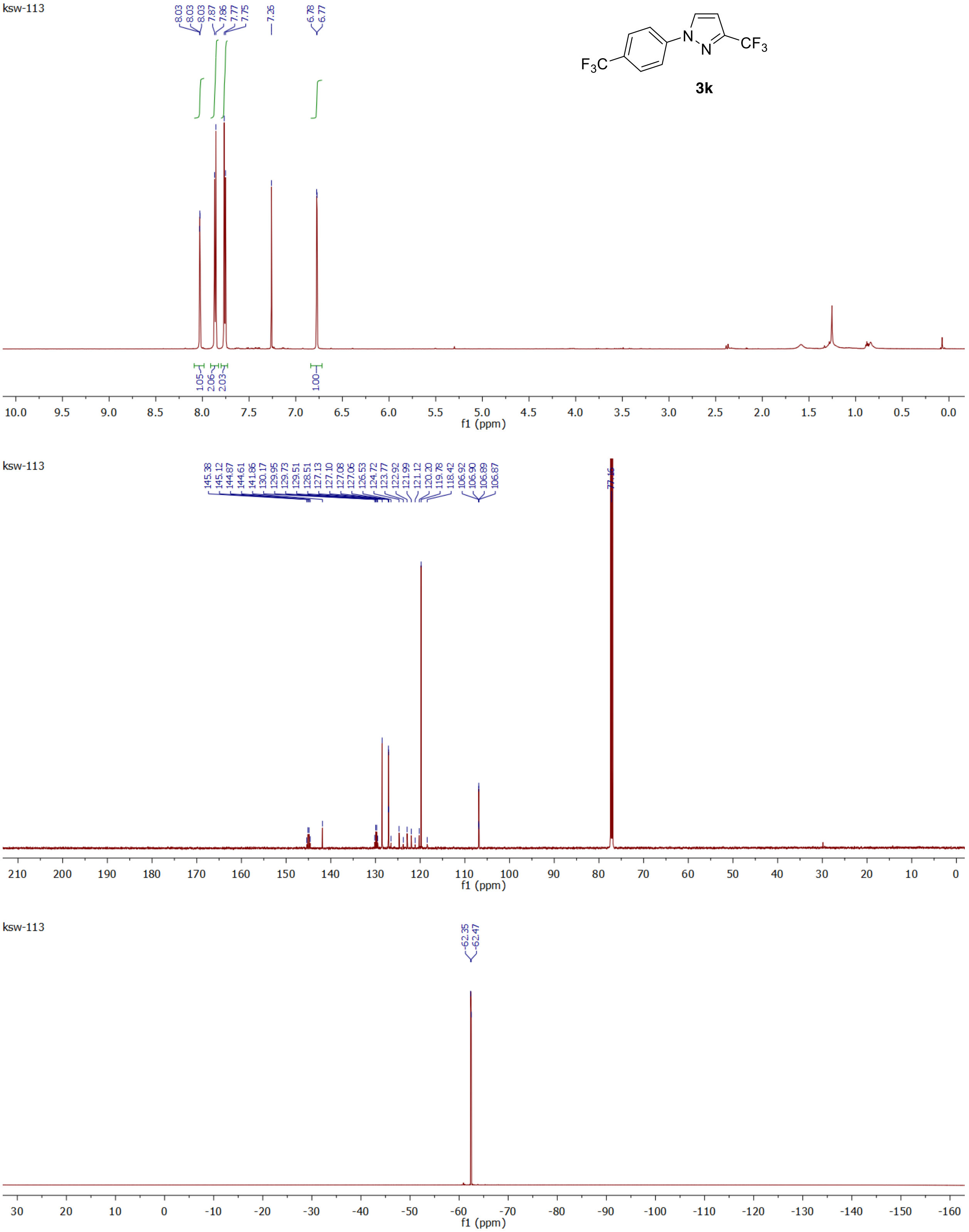

Fig S11. ^{1}H NMR (600 MHz, $CDCl_3$), ^{13}C NMR (151 MHz, $CDCl_3$) and ^{19}F NMR (565 MHz, $CDCl_3$) spectra for compound **3k**.

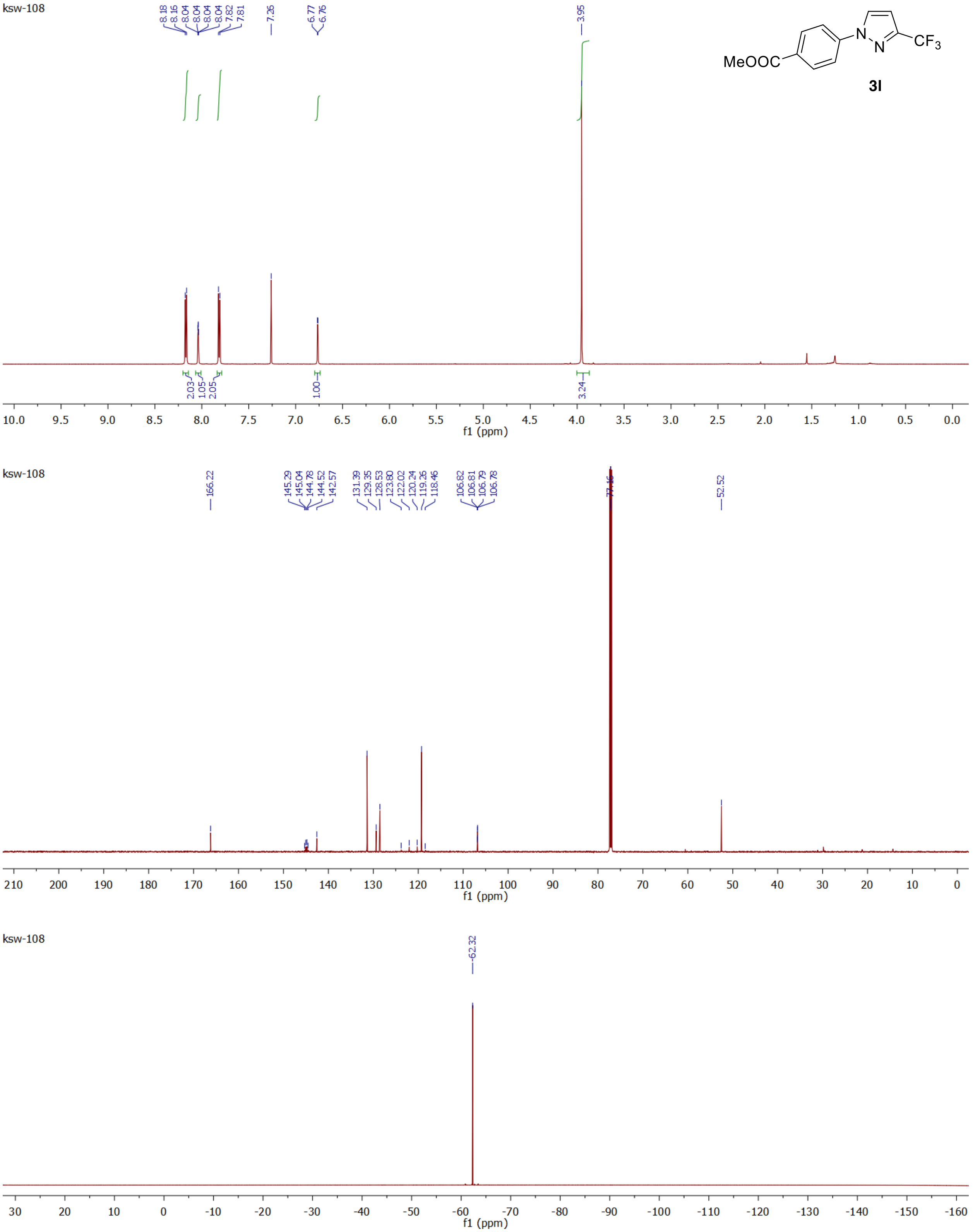

Fig S12. ^{1}H NMR (600 MHz, $CDCl_3$), ^{13}C NMR (151 MHz, $CDCl_3$) and ^{19}F NMR (565 MHz, $CDCl_3$) spectra for compound **3l**.

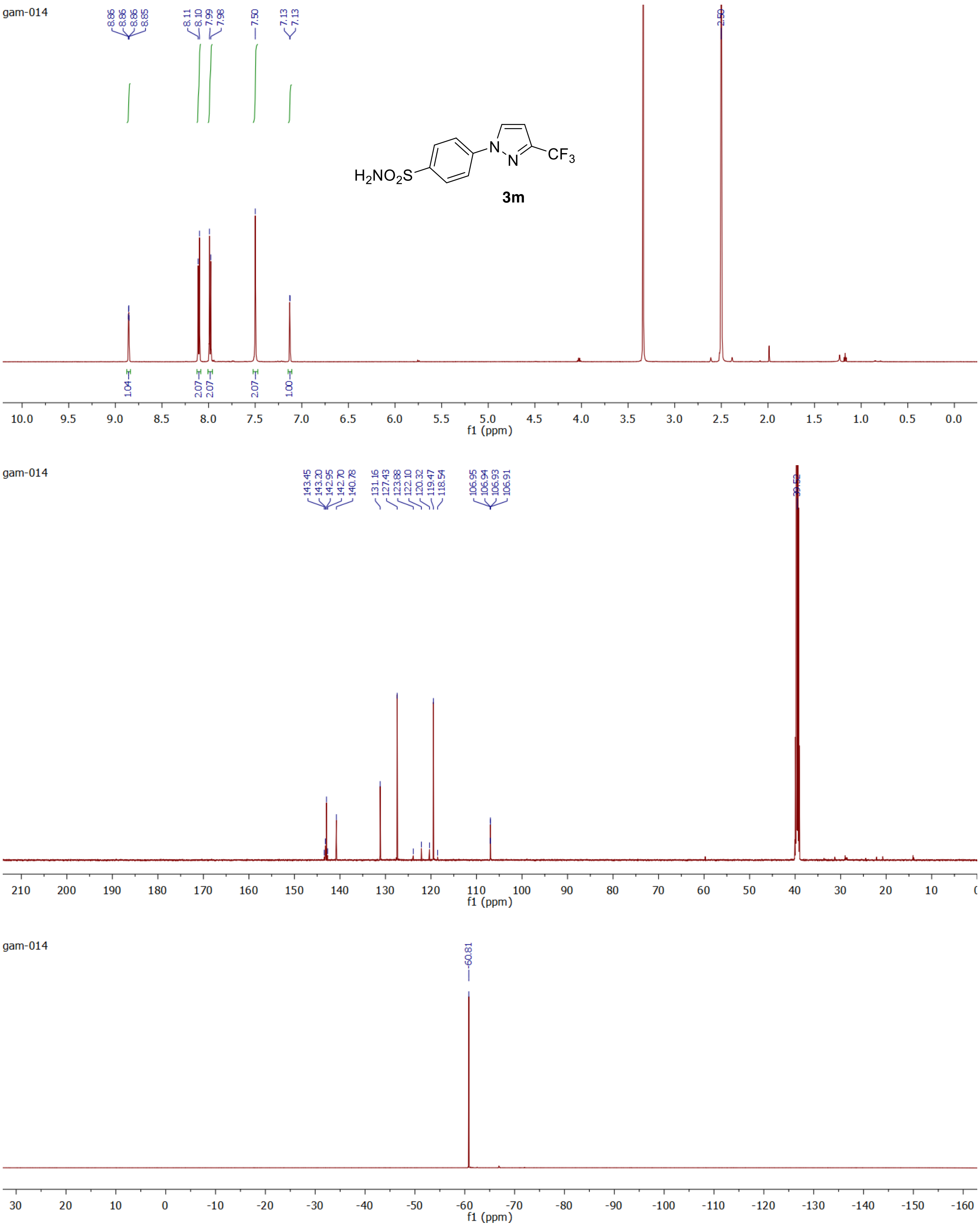

Fig S13. ^{1}H NMR (600 MHz), ^{13}C NMR (151 MHz) and ^{19}F NMR (565 MHz) spectra for compound **3m** (all taken in DMSO-d_6).

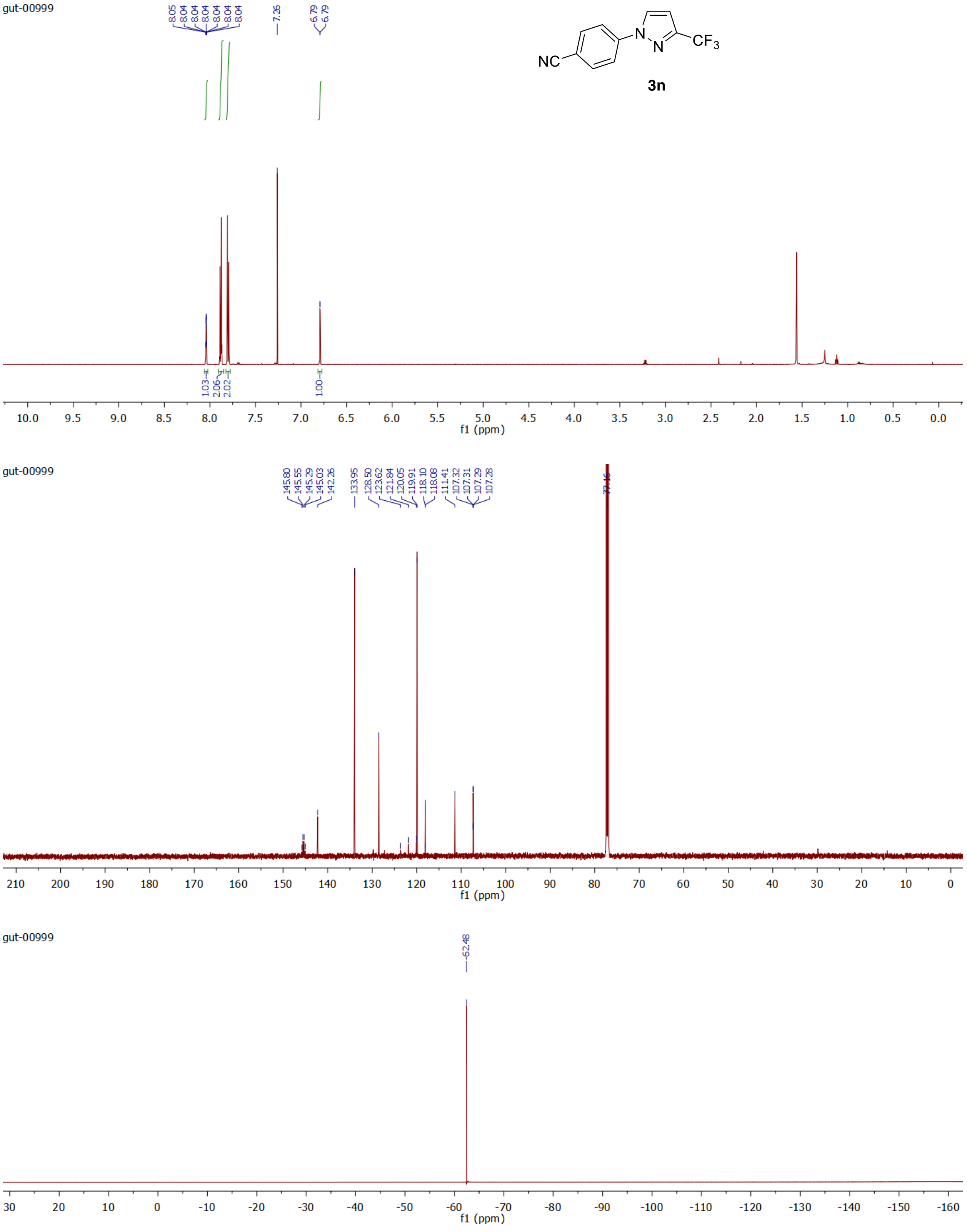

Fig S14. ^{1}H NMR (600 MHz, $CDCl_3$), ^{13}C NMR (151 MHz, $CDCl_3$) and ^{19}F NMR (565 MHz, $CDCl_3$) spectra for compound **3n**.

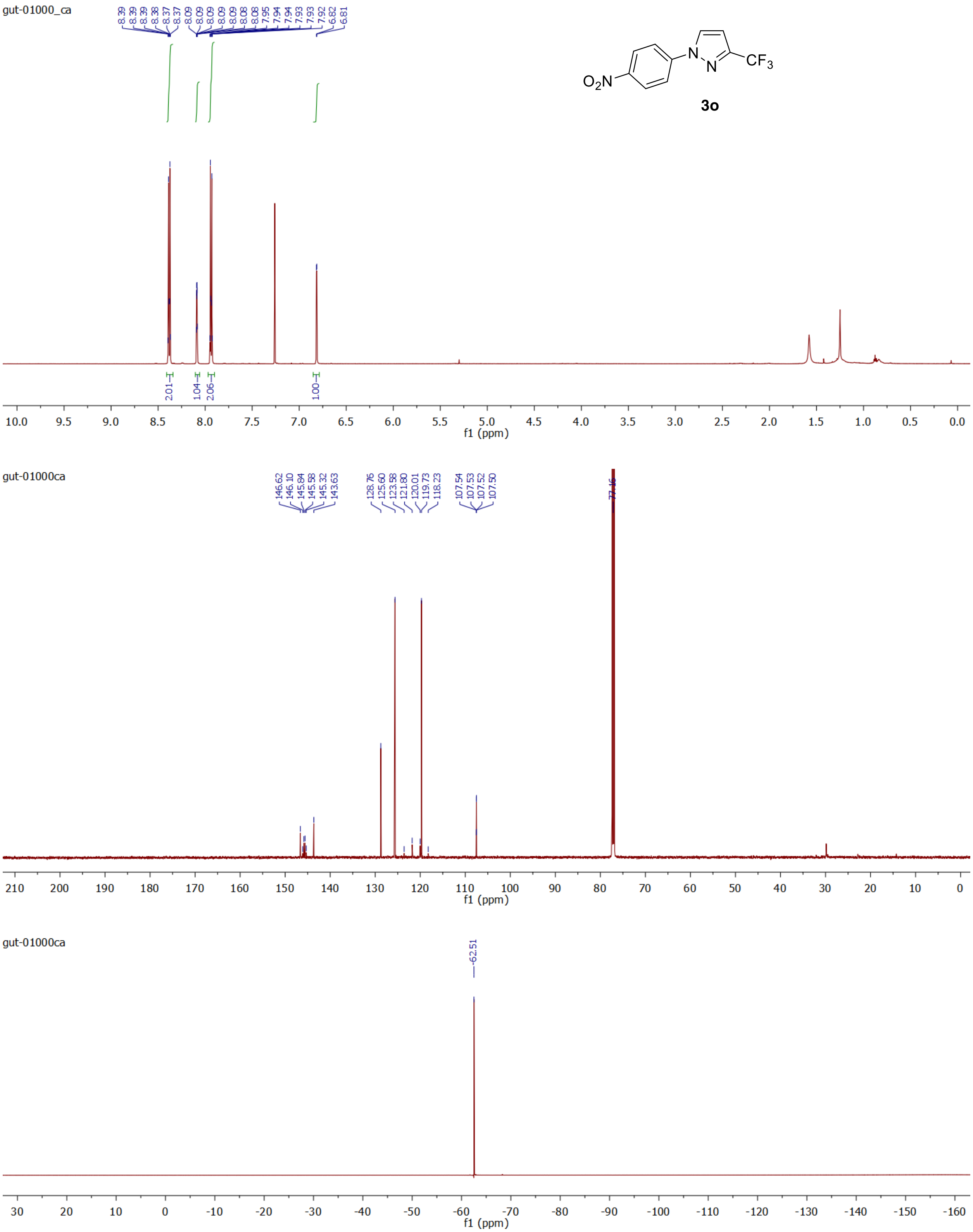

Fig S15. ^{1}H NMR (600 MHz, $CDCl_3$), ^{13}C NMR (151 MHz, $CDCl_3$) and ^{19}F NMR (565 MHz, $CDCl_3$) spectra for compound **3o**.

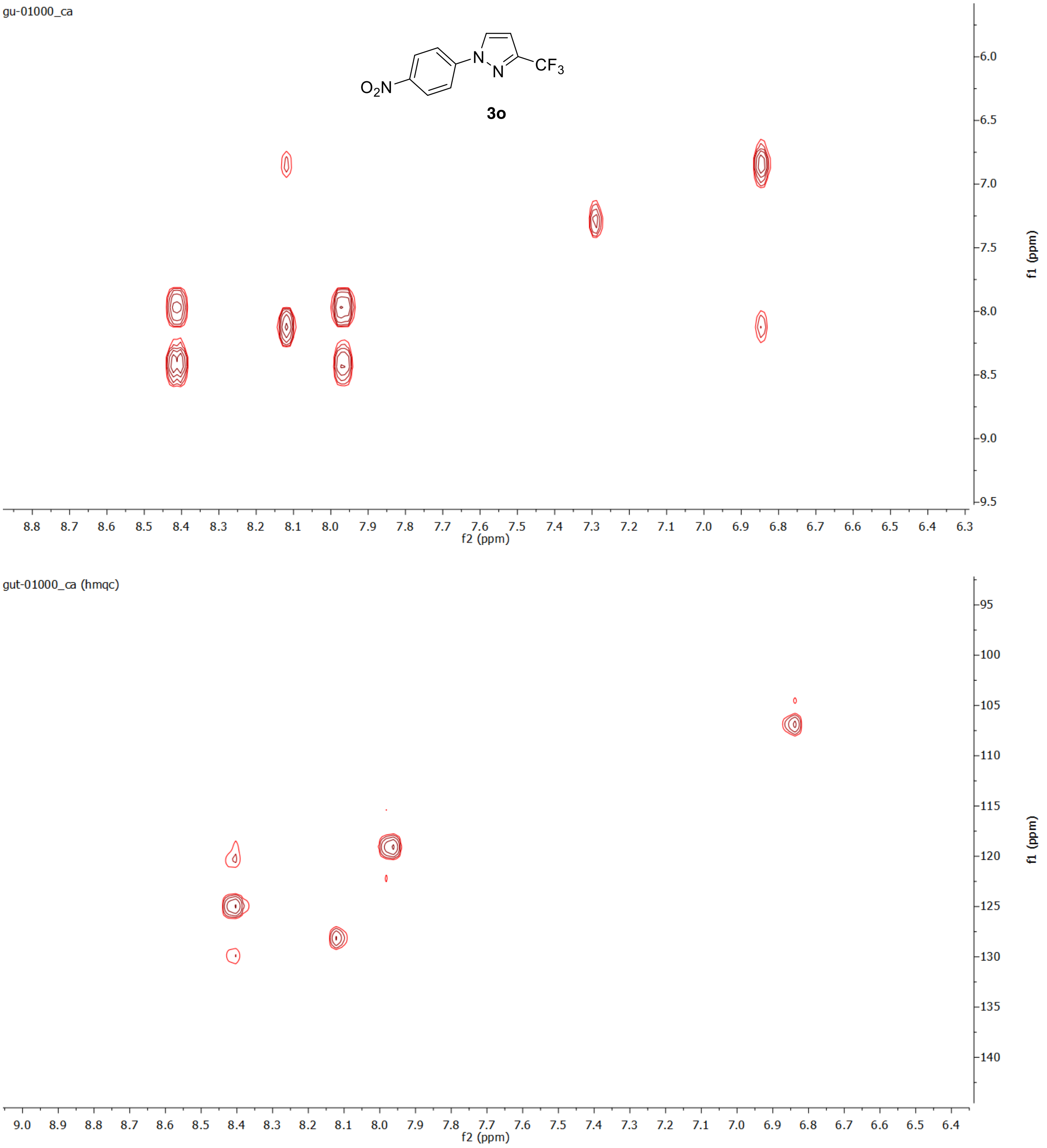

Fig S16. Diagnostic parts of 2D spectra (cosy, hmqc) for compound **3o**.

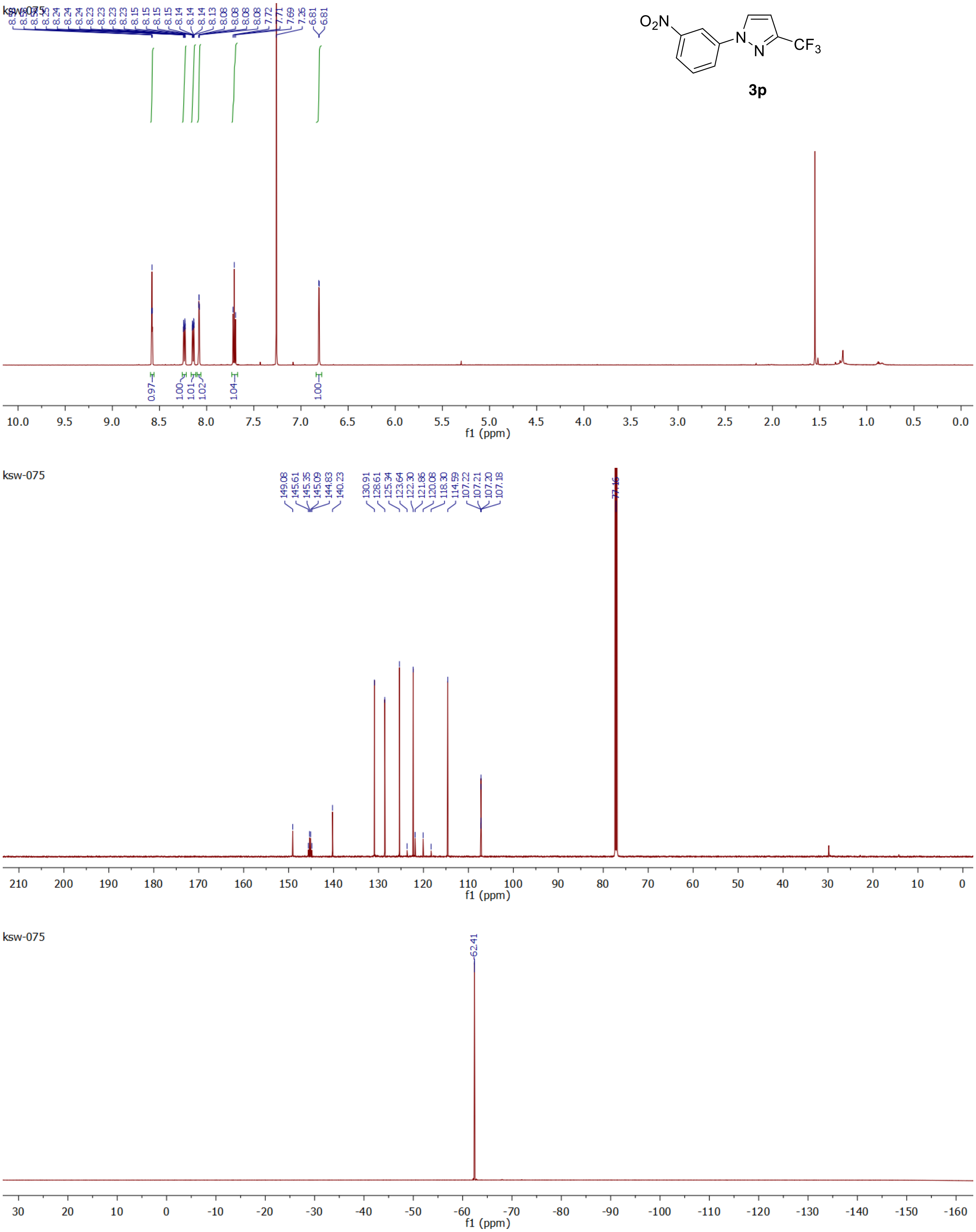

Fig S17. ^{1}H NMR (600 MHz, $CDCl_3$), ^{13}C NMR (151 MHz, $CDCl_3$) and ^{19}F NMR (565 MHz, $CDCl_3$) spectra for compound **3p**.

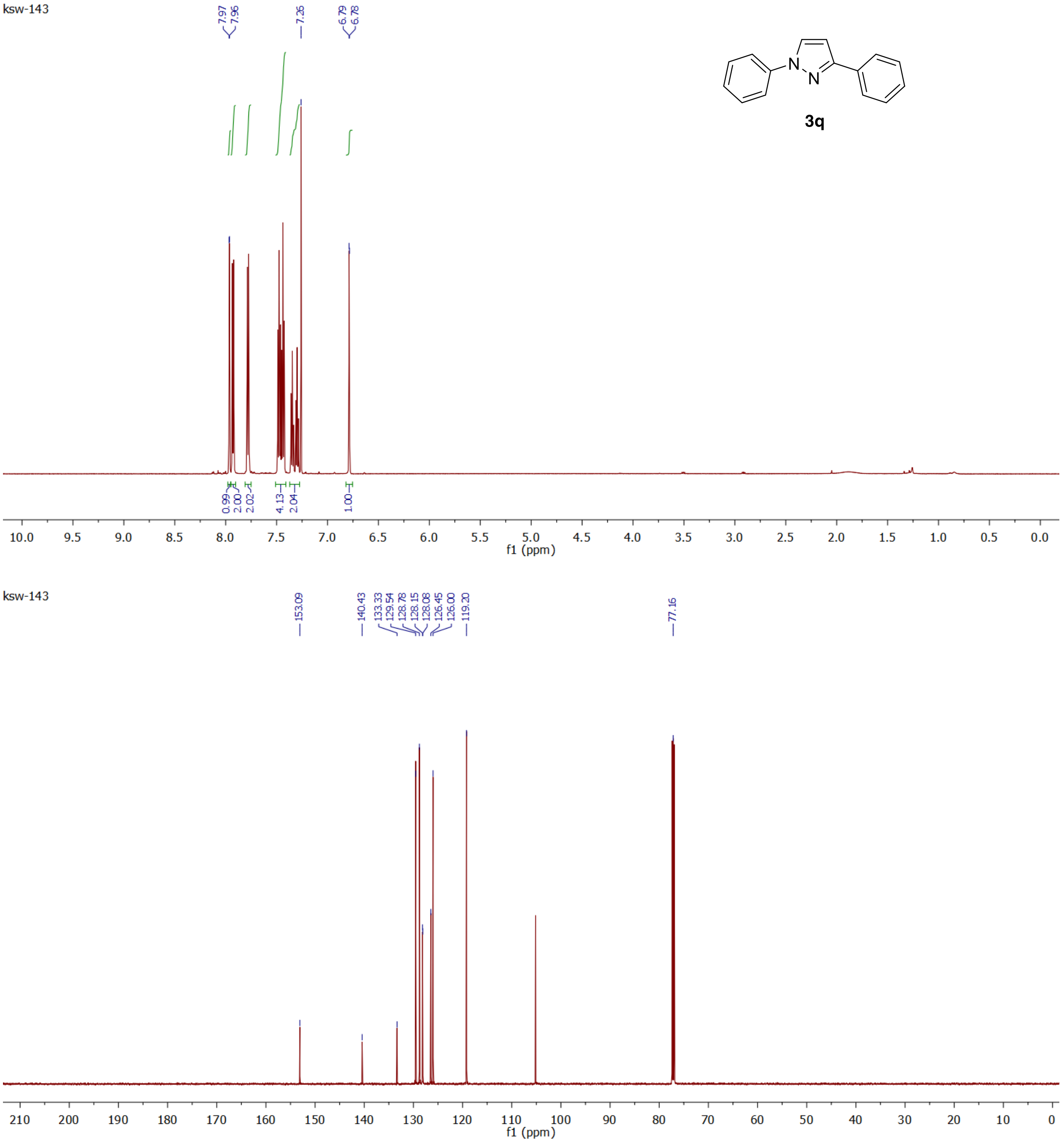

Fig S18. ^{1}H NMR (600 MHz, $CDCl_3$) and ^{13}C NMR (151 MHz, $CDCl_3$) spectra for compound **3q**.

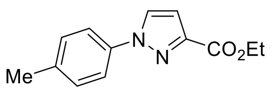

3r

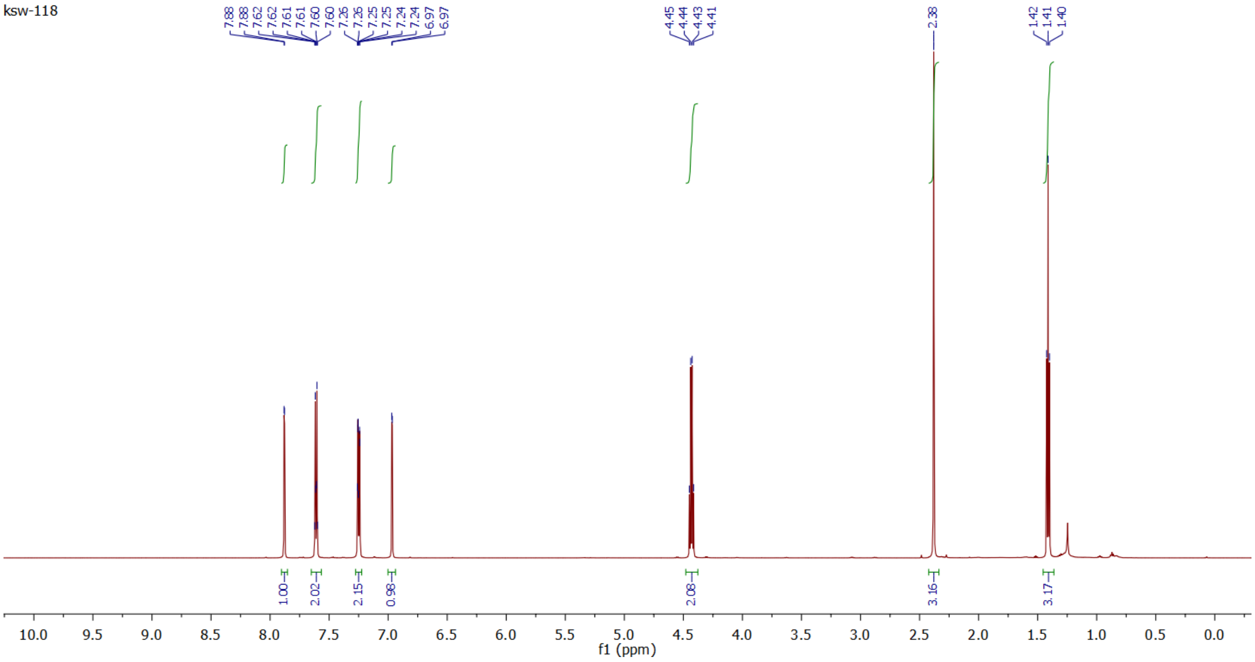

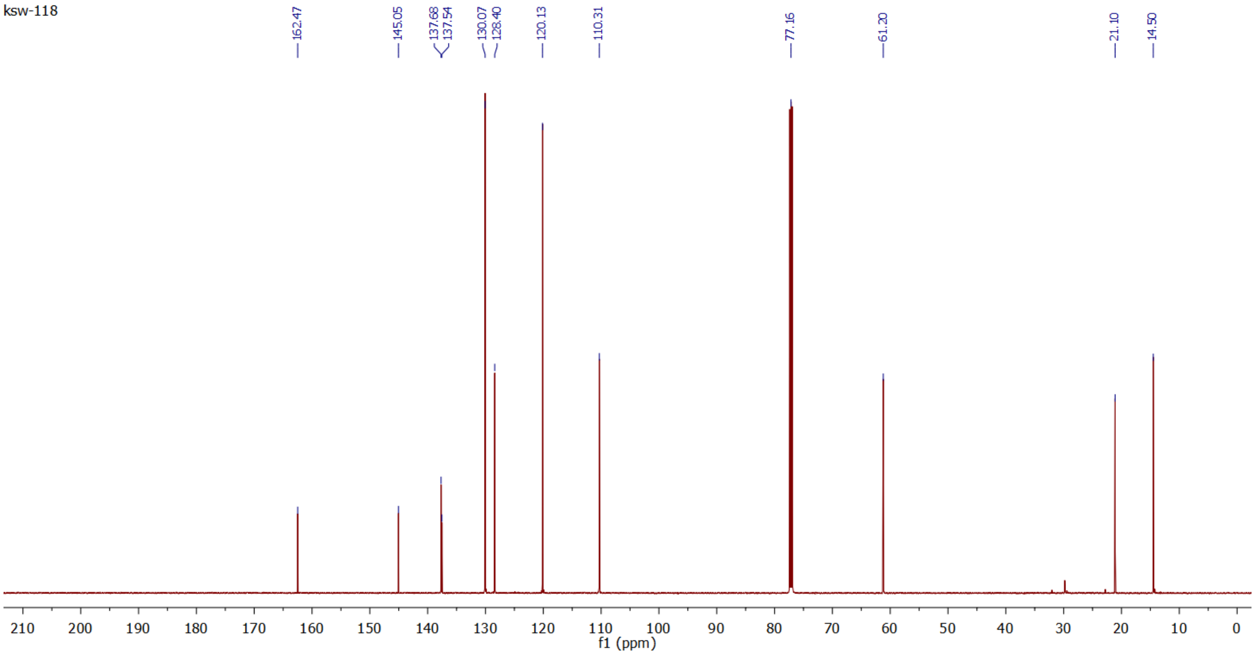

Fig S19. ^{1}H NMR (600 MHz, $CDCl_3$) and ^{13}C NMR (151 MHz, $CDCl_3$) spectra for compound **3r**.

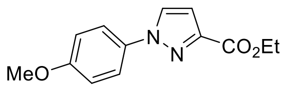

3s

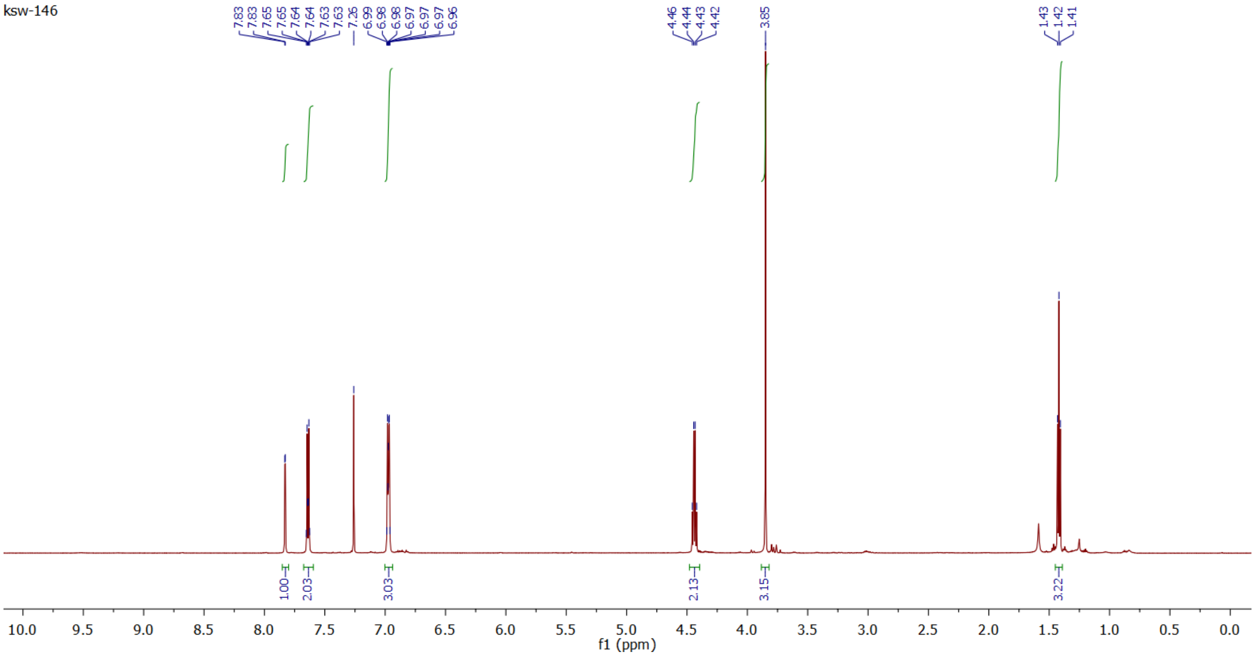

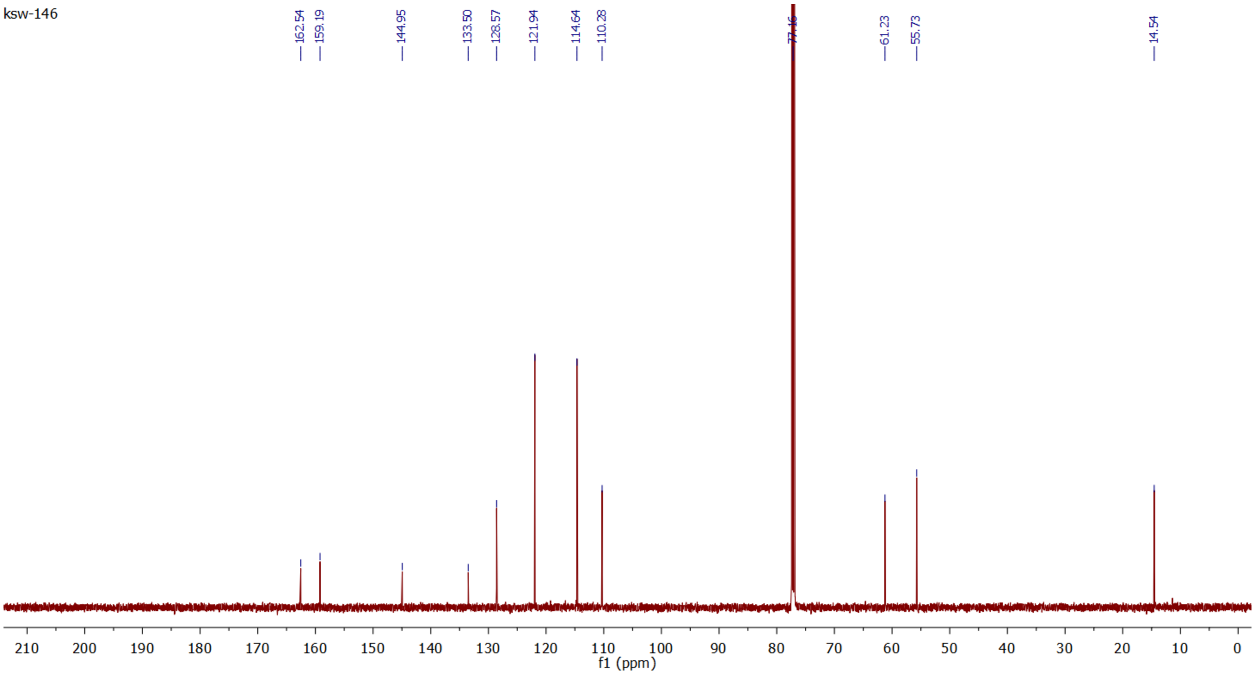

Fig S20. ^{1}H NMR (600 MHz, $CDCl_3$) and ^{13}C NMR (151 MHz, $CDCl_3$) spectra for compound **3s**.

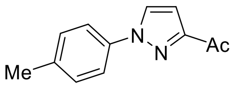

3t

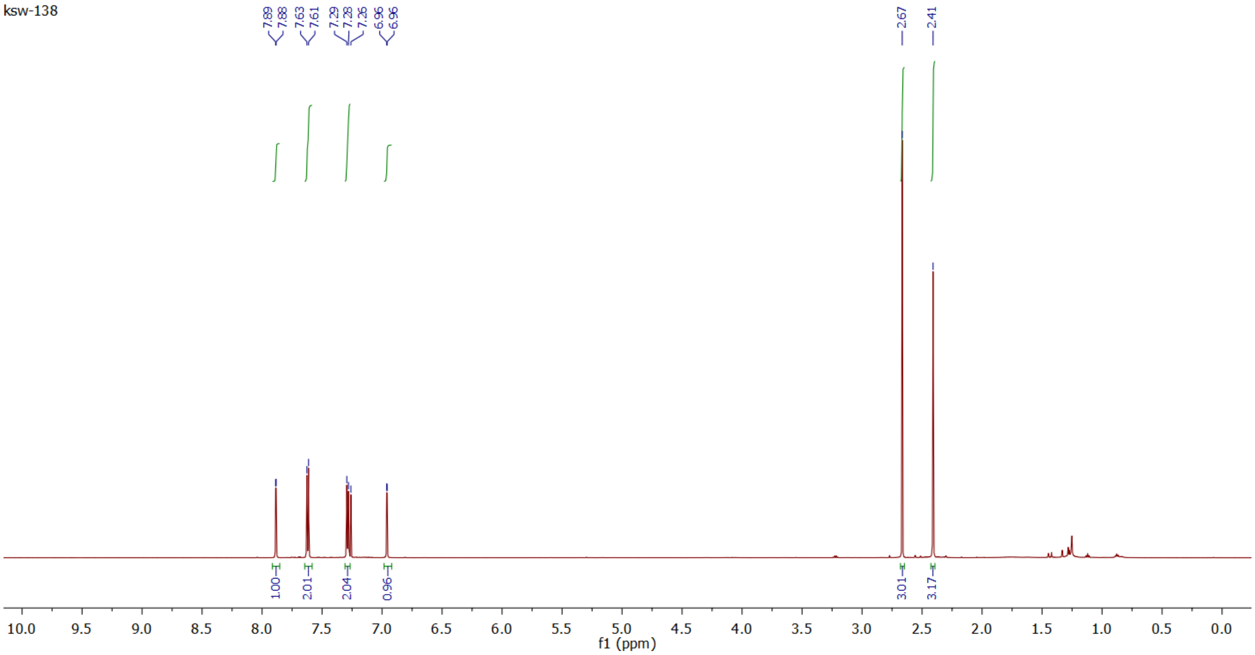

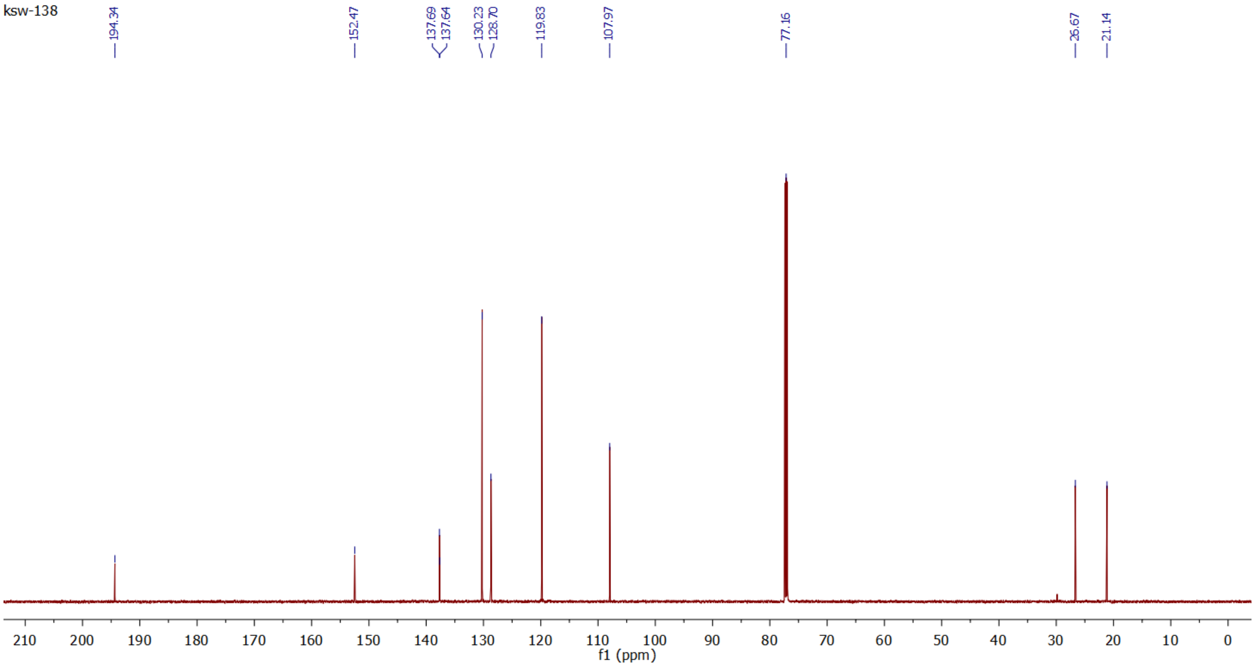

Fig S21. ^{1}H NMR (600 MHz, $CDCl_3$) and ^{13}C NMR (151 MHz, $CDCl_3$) spectra for compound **3t**.

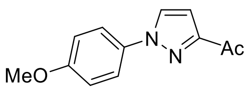

3u

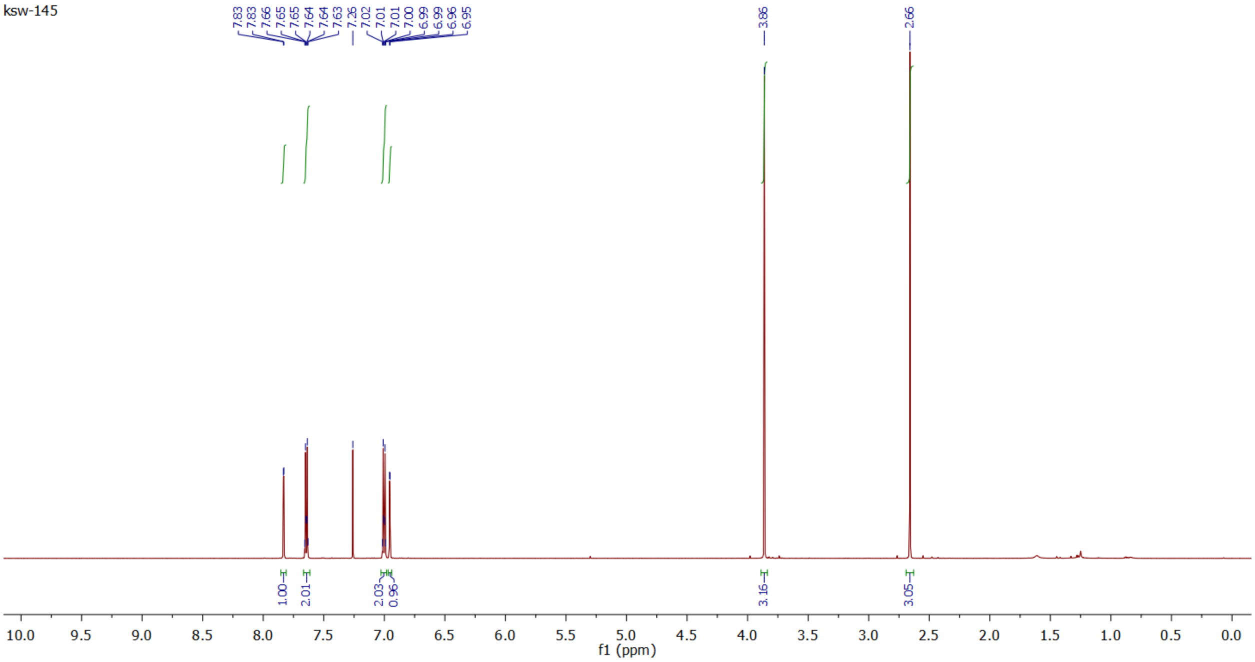

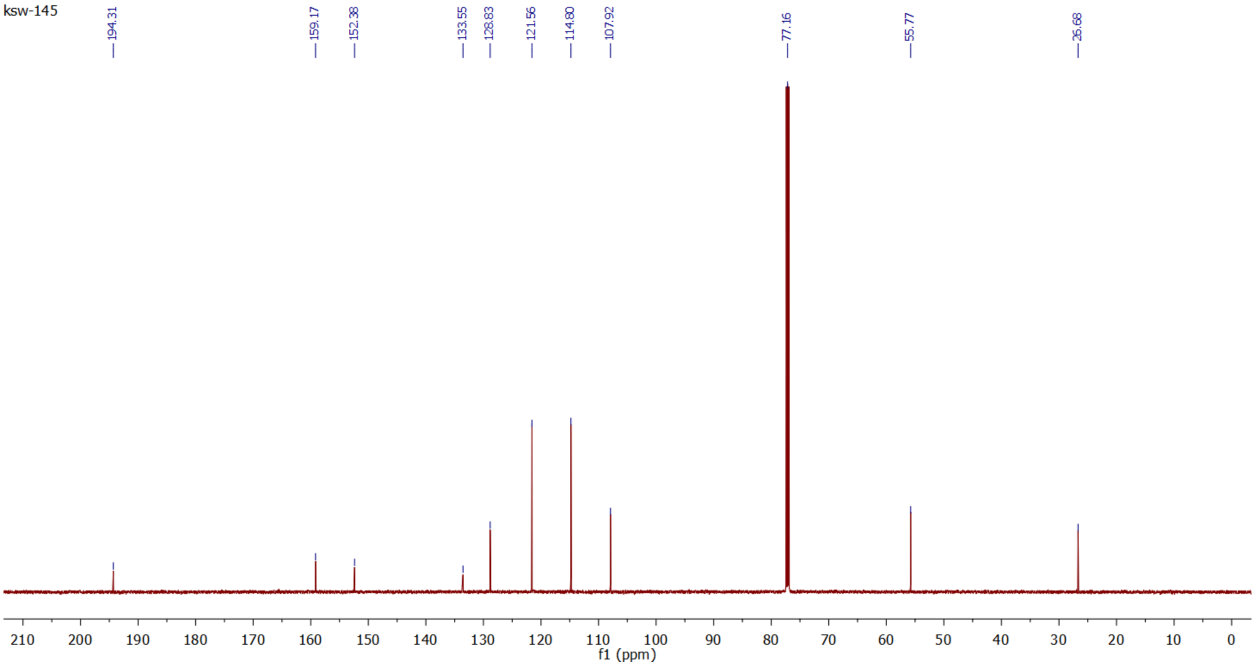

Fig S22. ^{1}H NMR (600 MHz, $CDCl_3$) and ^{13}C NMR (151 MHz, $CDCl_3$) spectra for compound **3u**.

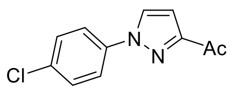

3v

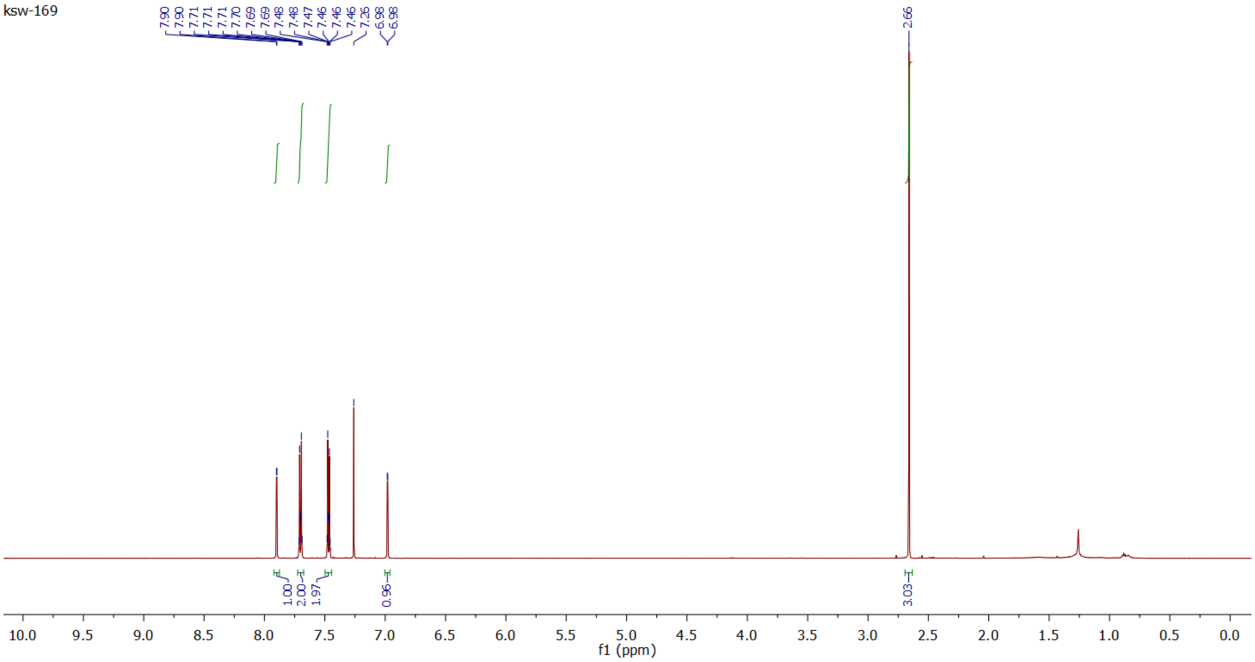

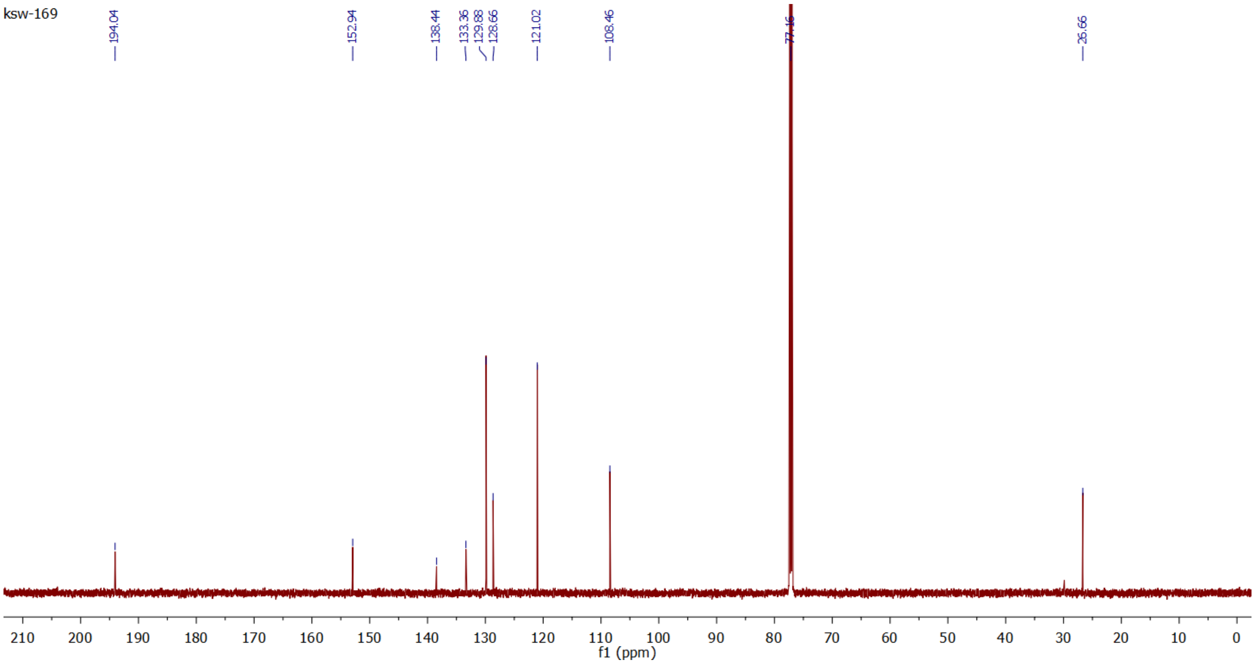

Fig S23. ^{1}H NMR (600 MHz, $CDCl_3$) and ^{13}C NMR (151 MHz, $CDCl_3$) spectra for compound **3v**.

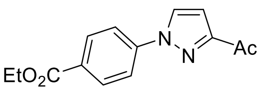

3w

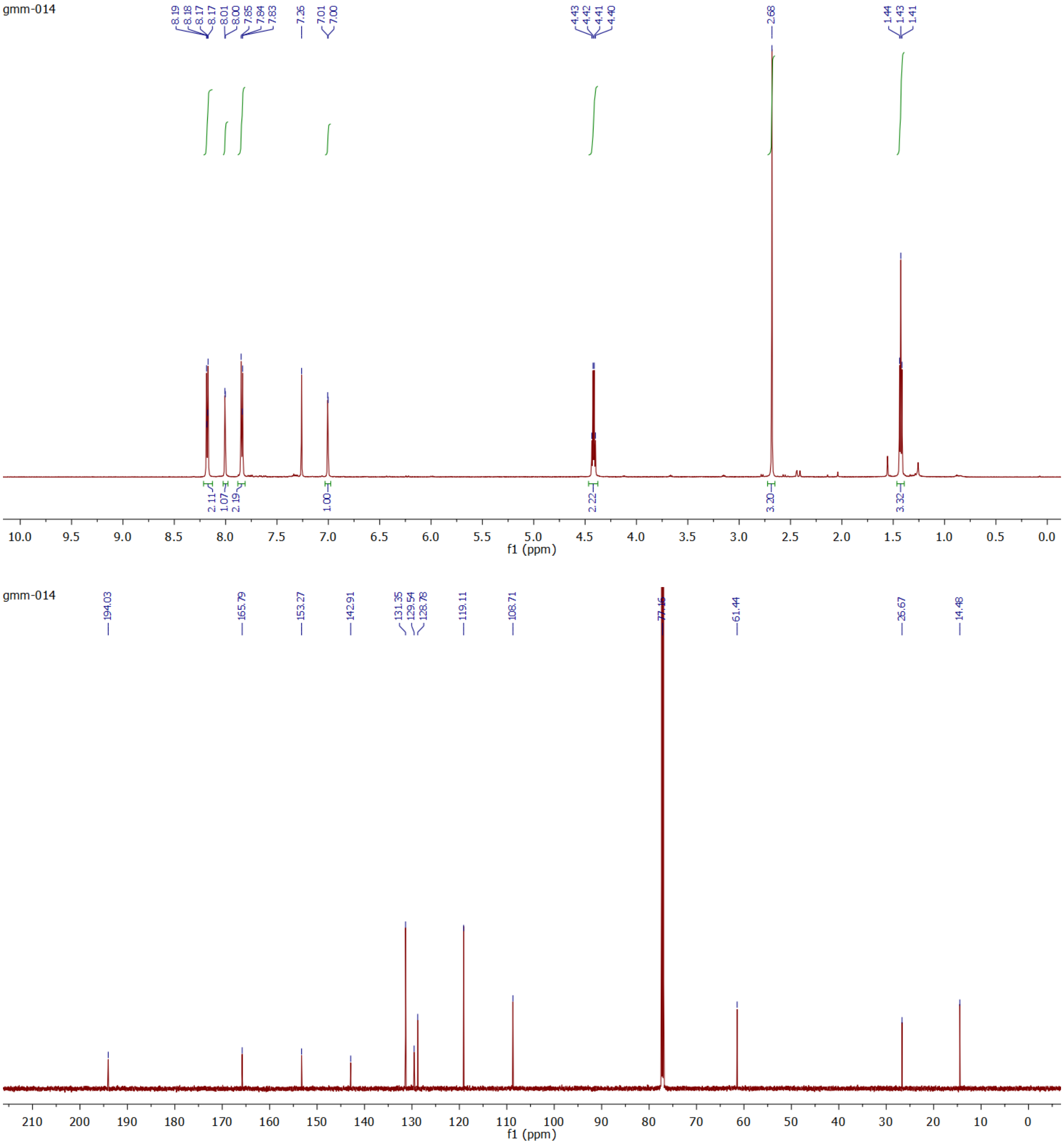

Fig S24. ^{1}H NMR (600 MHz, $CDCl_3$) and ^{13}C NMR (151 MHz, $CDCl_3$) spectra for compound **3w**.

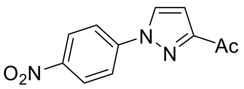

3x

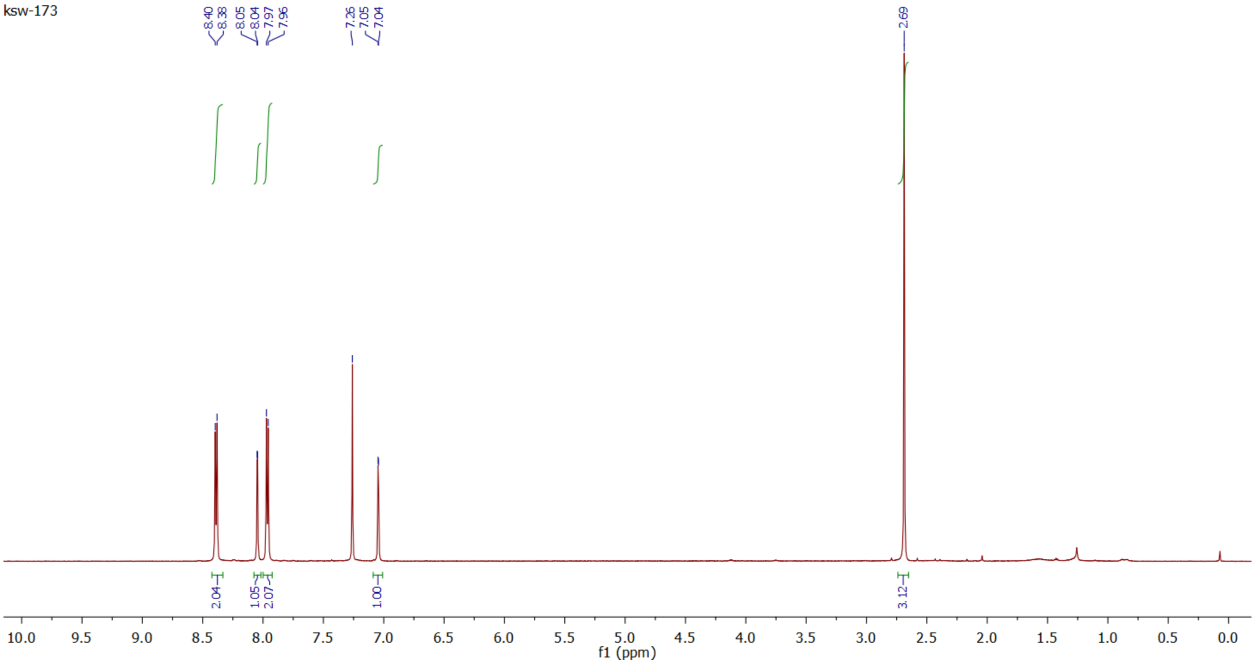

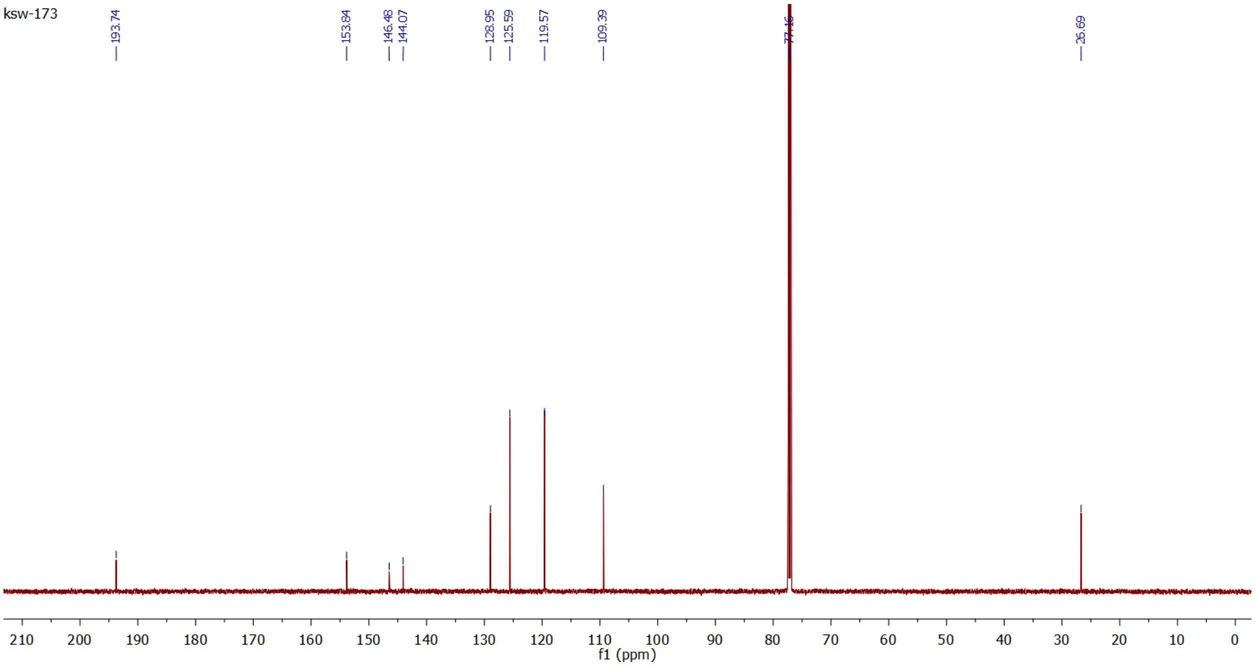

Fig S25. ^{1}H NMR (600 MHz, $CDCl_3$) and ^{13}C NMR (151 MHz, $CDCl_3$) spectra for compound **3x**.

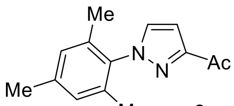

3y

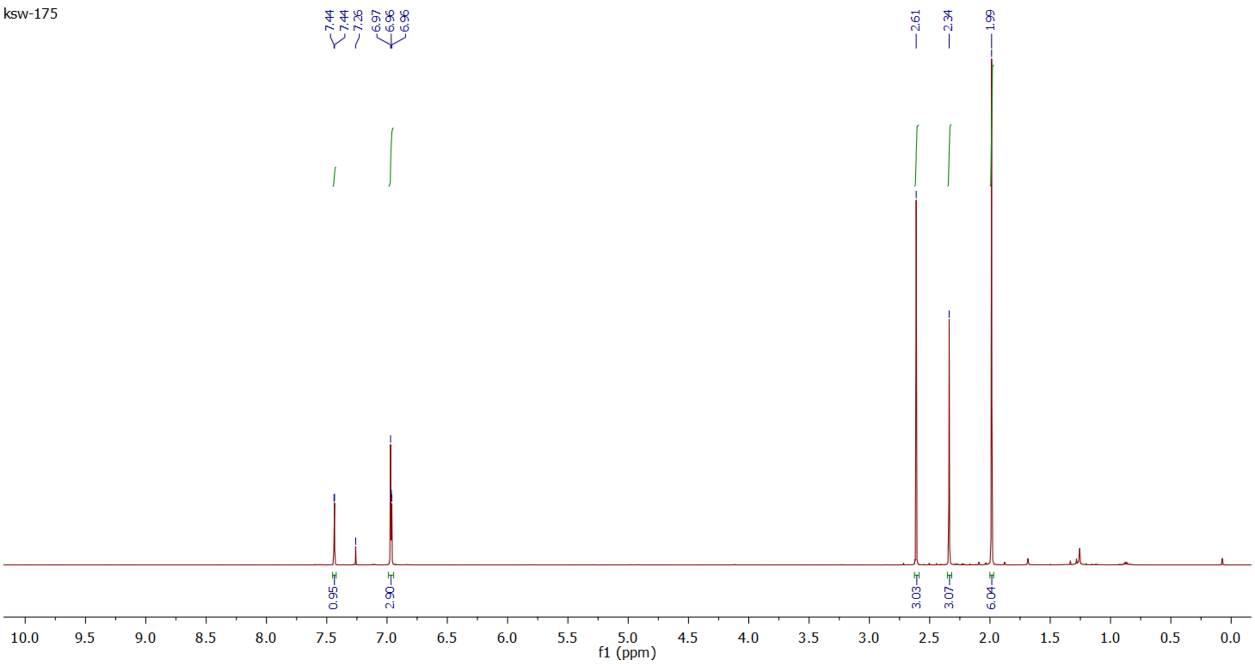

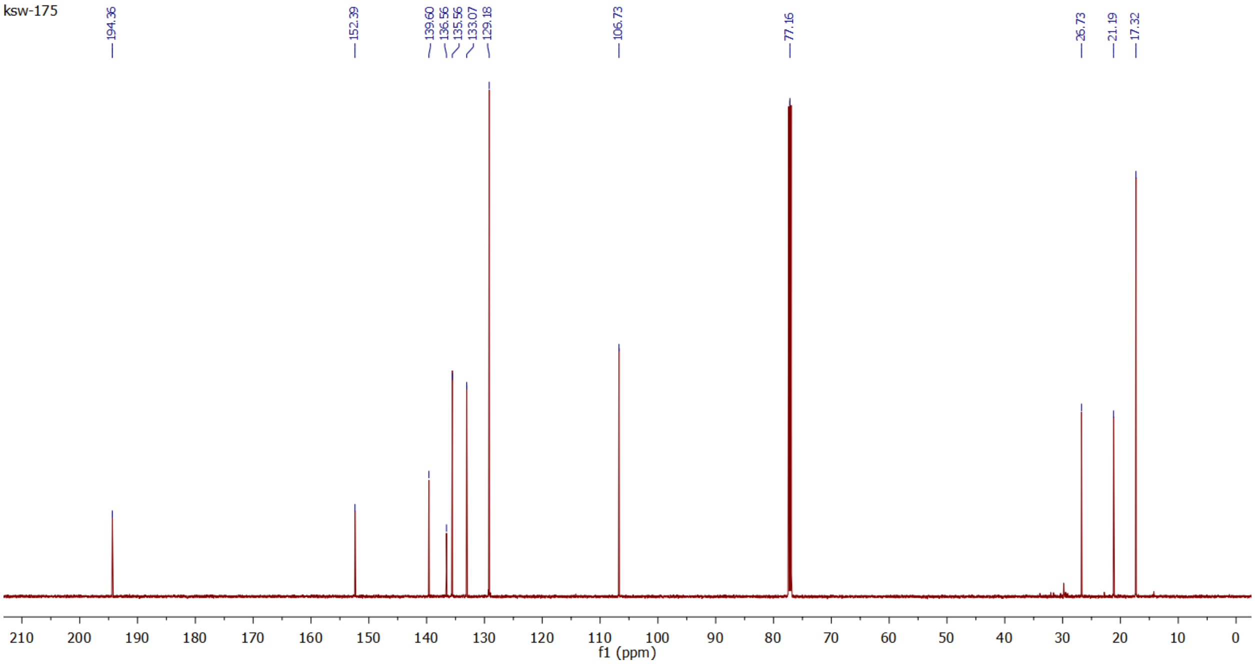

Fig S26. ^{1}H NMR (600 MHz, $CDCl_3$) and ^{13}C NMR (151 MHz, $CDCl_3$) spectra for compound **3y**.

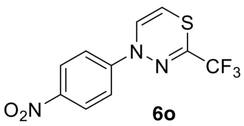

6o

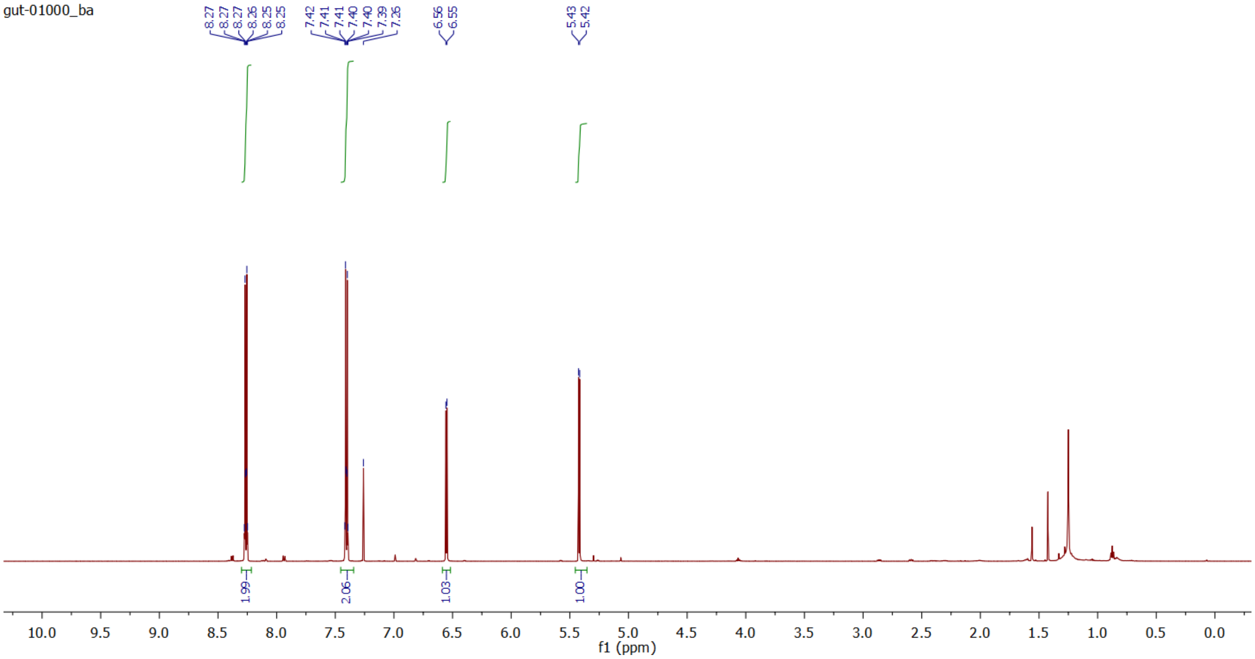

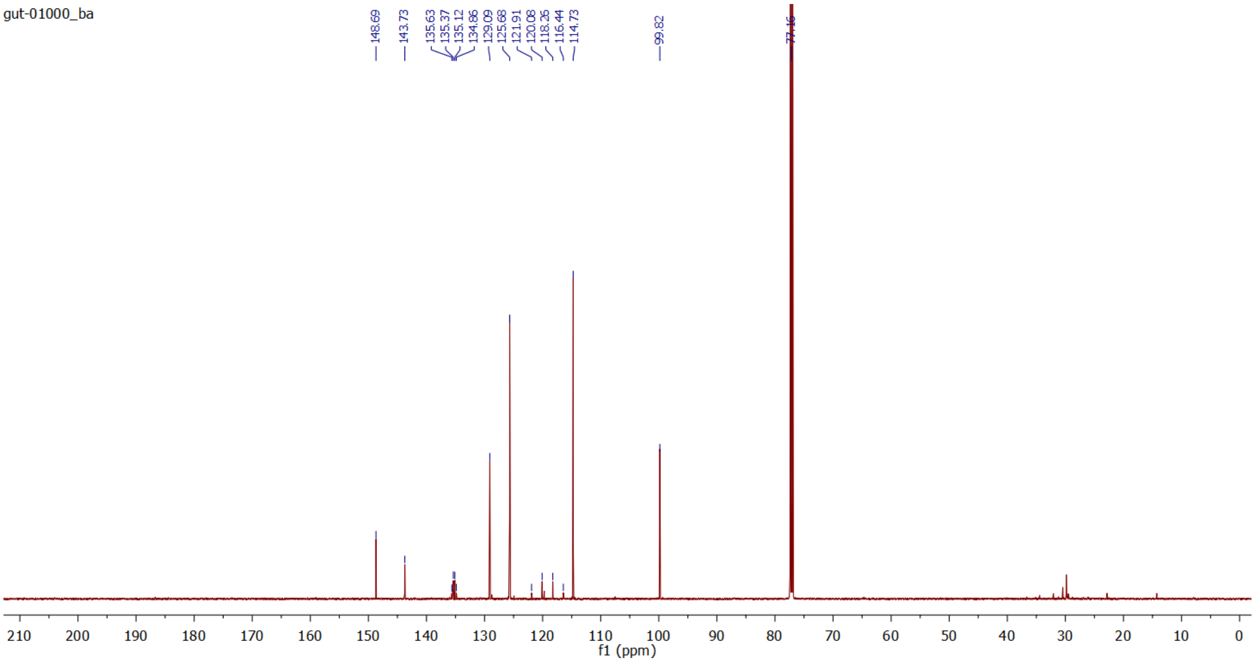

Fig S27. ^{1}H NMR (600 MHz, $CDCl_3$) and ^{13}C NMR (151 MHz, $CDCl_3$) spectra for compound **6o**.

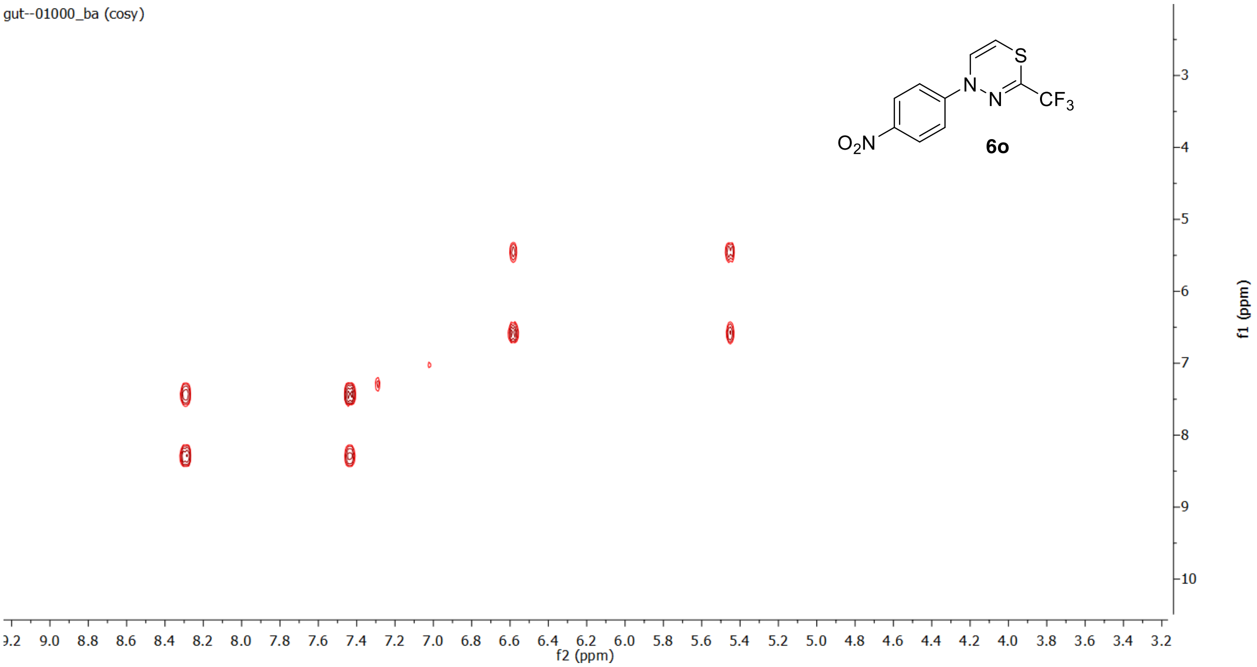

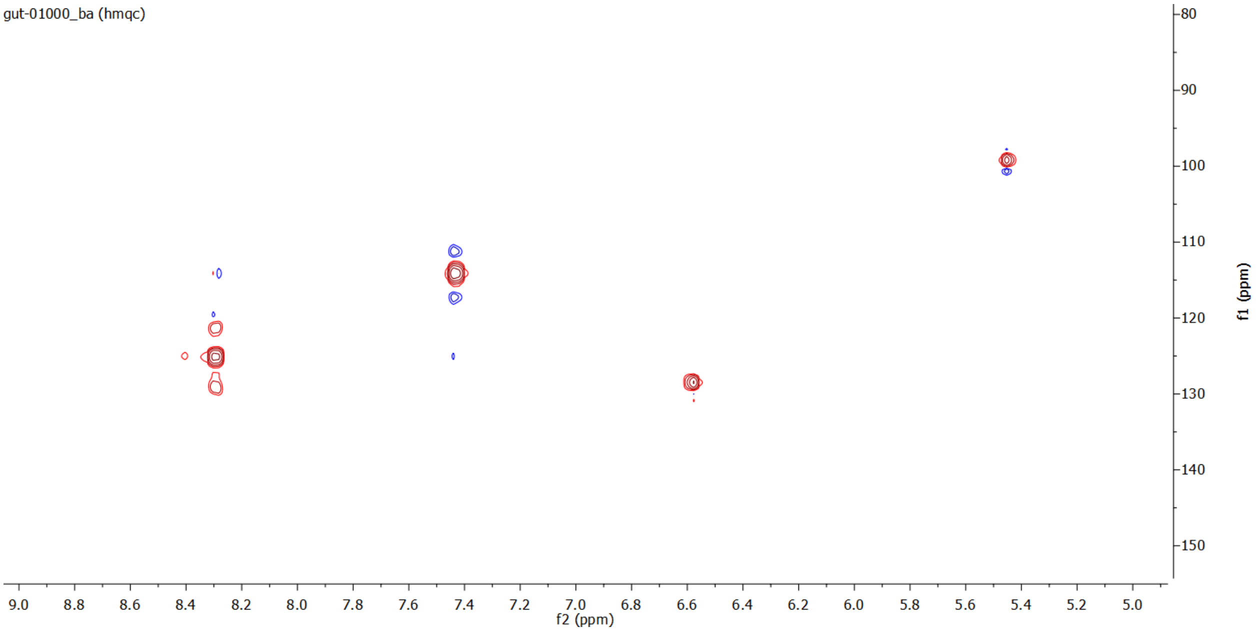

Fig S28. Diagnostic parts of 2D spectra (cosy, hmqc) for compound **6o**.

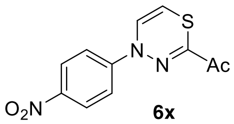

6x

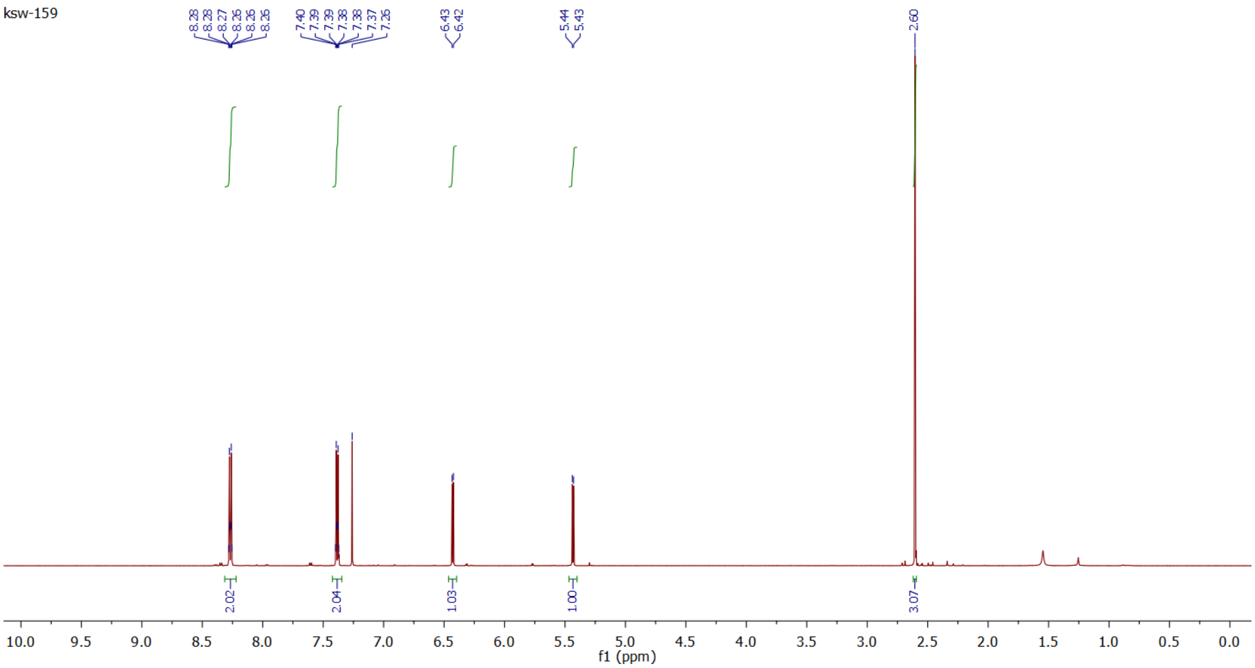

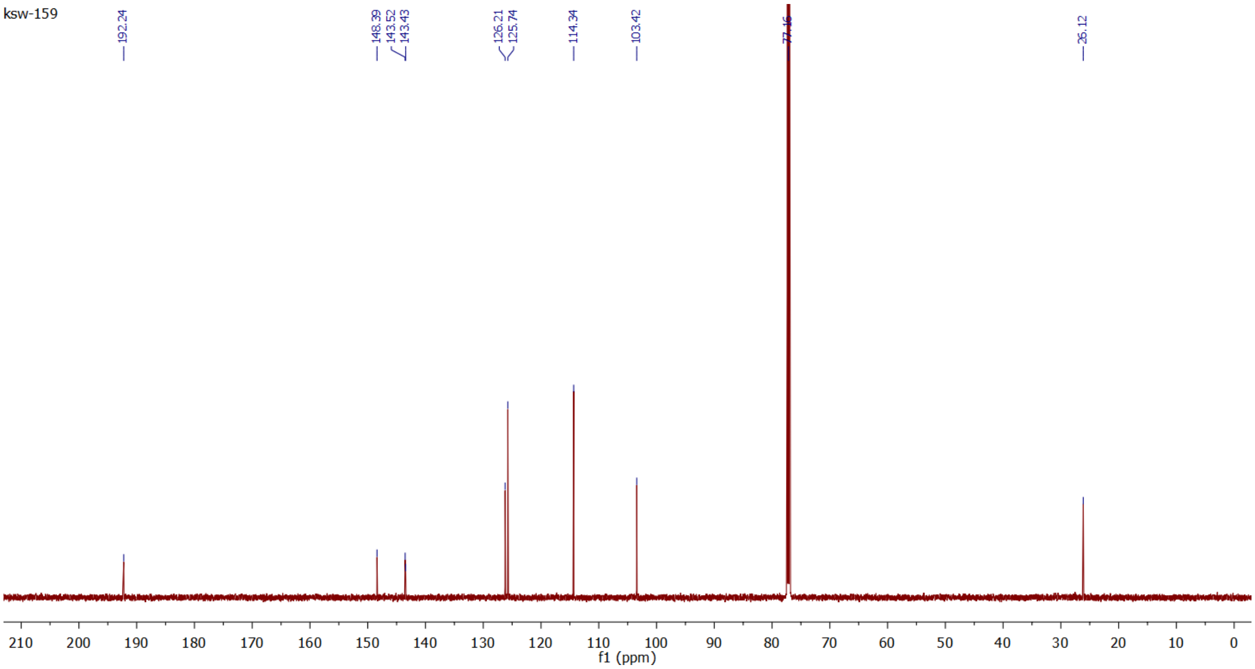

Fig S29. ^{1}H NMR (600 MHz, $CDCl_3$) and ^{13}C NMR (151 MHz, $CDCl_3$) spectra for compound **6x**.

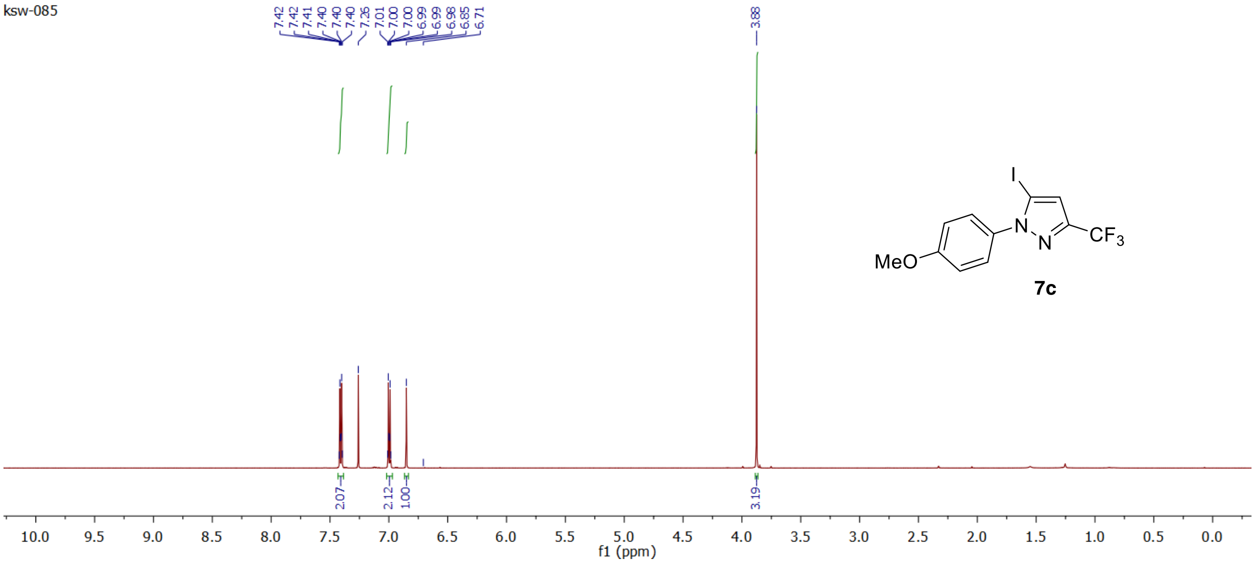

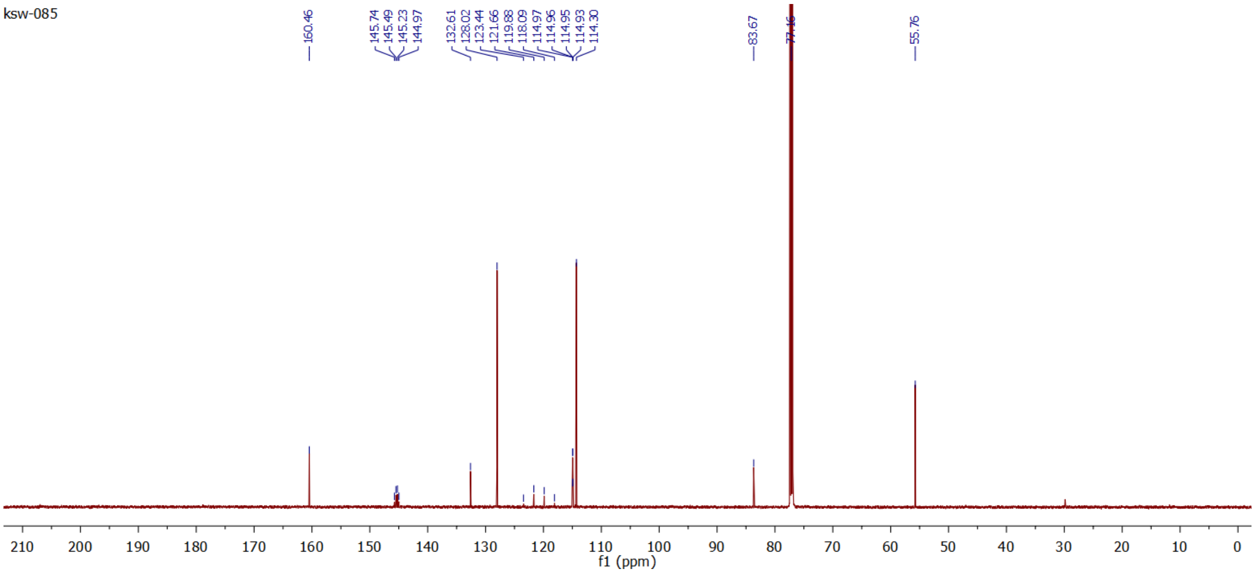

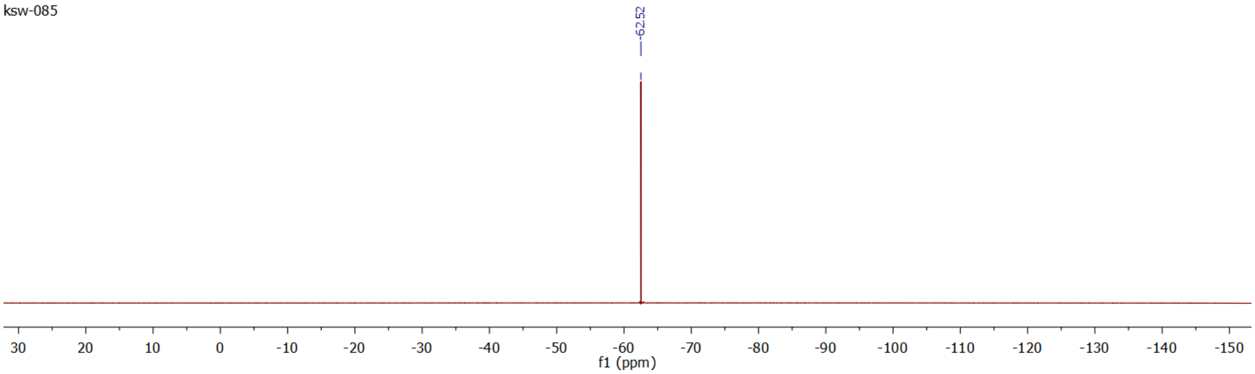

Fig S30. ^{1}H NMR (600 MHz, $CDCl_3$), ^{13}C NMR (151 MHz, $CDCl_3$) and ^{19}F NMR (565 MHz, $CDCl_3$) spectra for compound **7c**.

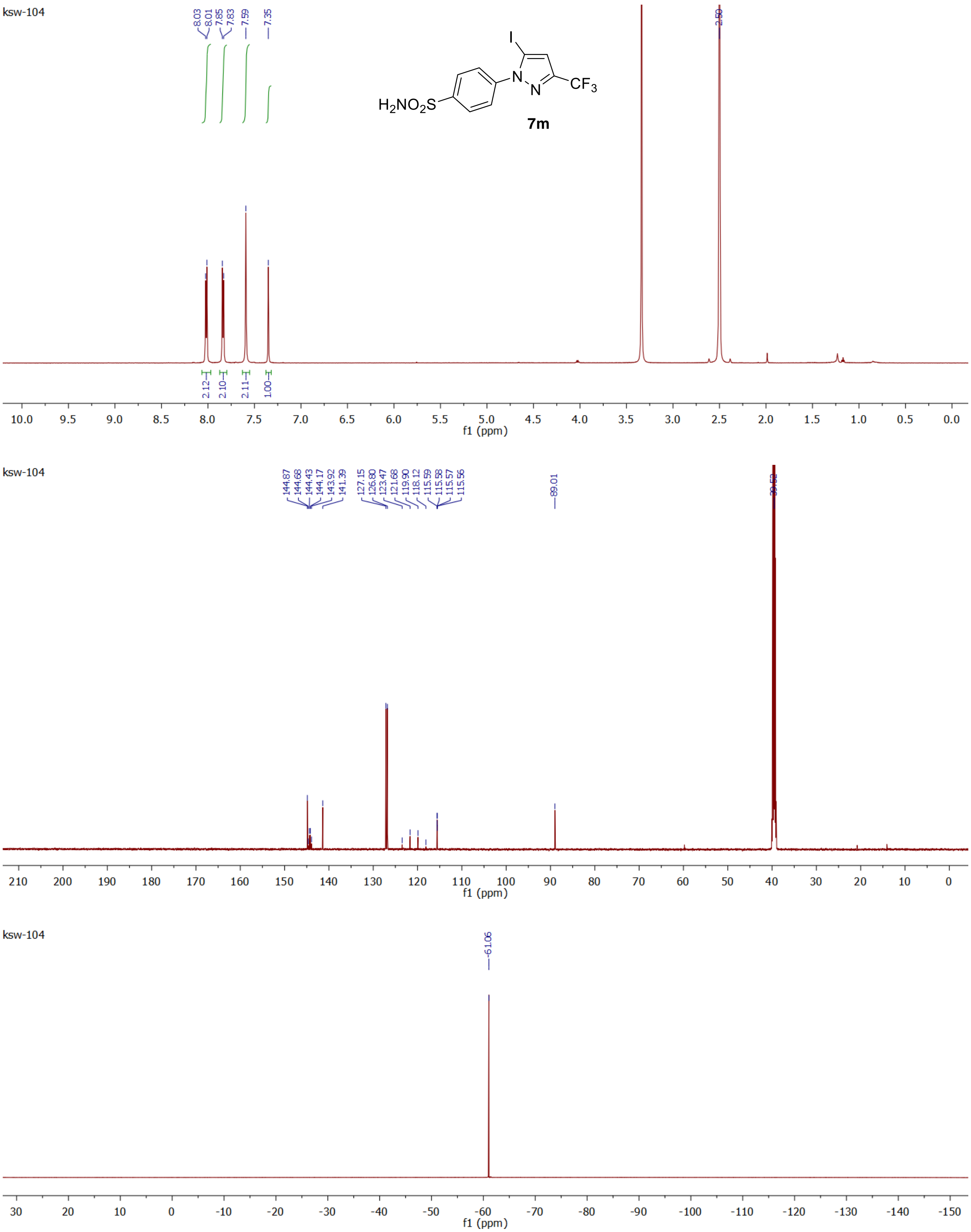

Fig S31. ^{1}H NMR (600 MHz, DMSO-d_6), ^{13}C NMR (151 MHz, DMSO-d_6) and ^{19}F NMR (565 MHz, DMSO-d_6) spectra for compound **7m**.

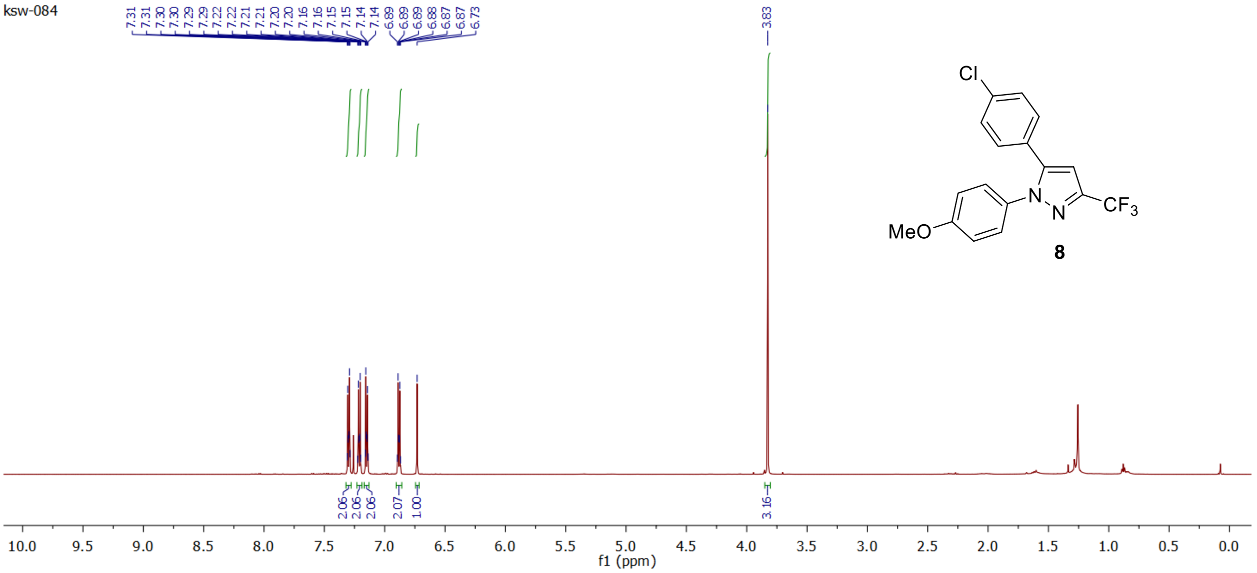

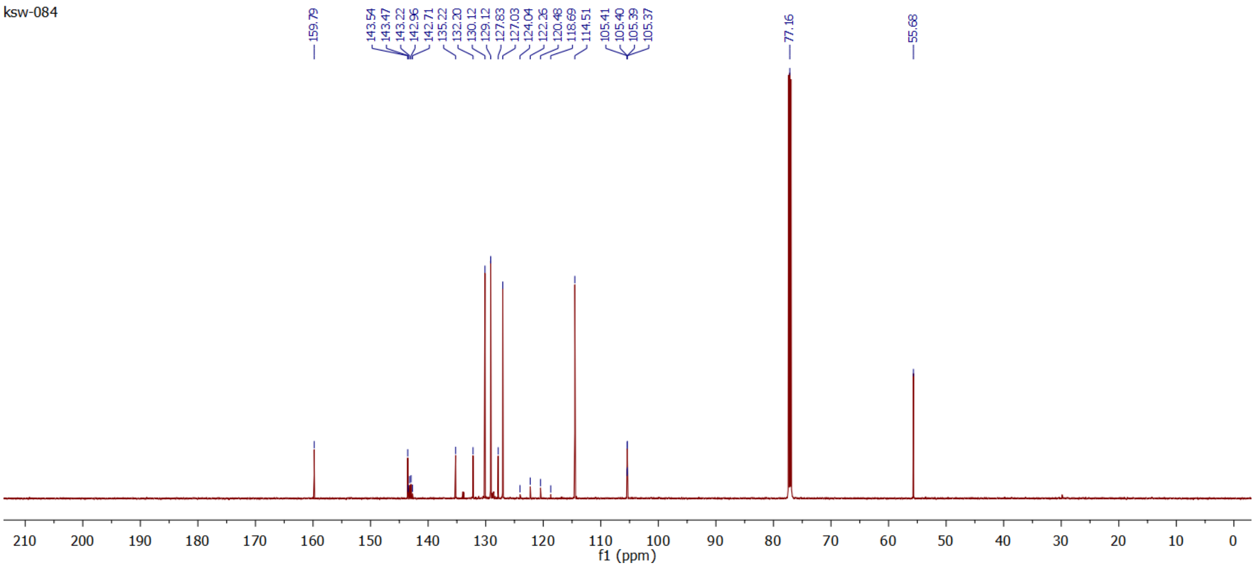

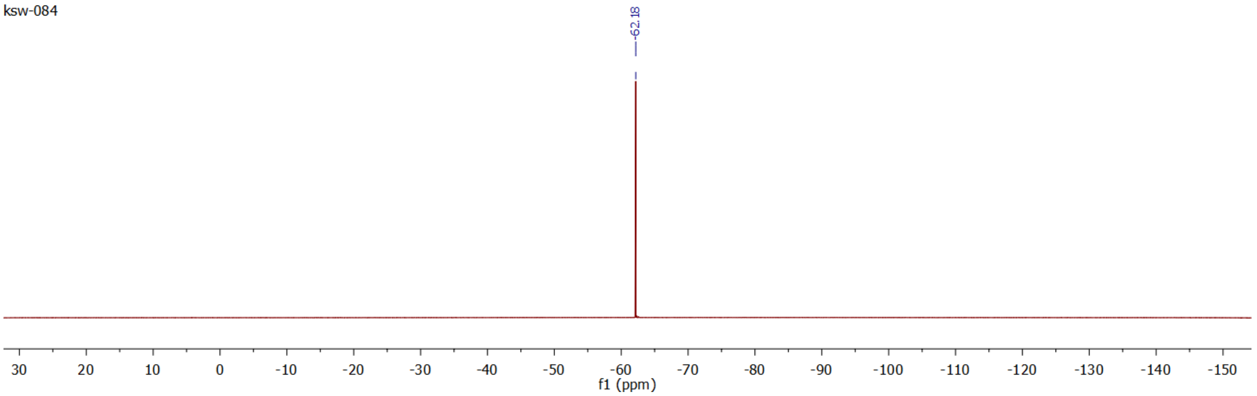

Fig S32. ^{1}H NMR (600 MHz, $CDCl_3$), ^{13}C NMR (151 MHz, $CDCl_3$) and ^{19}F NMR (565 MHz, $CDCl_3$) spectra for compound **8** (SC-560).

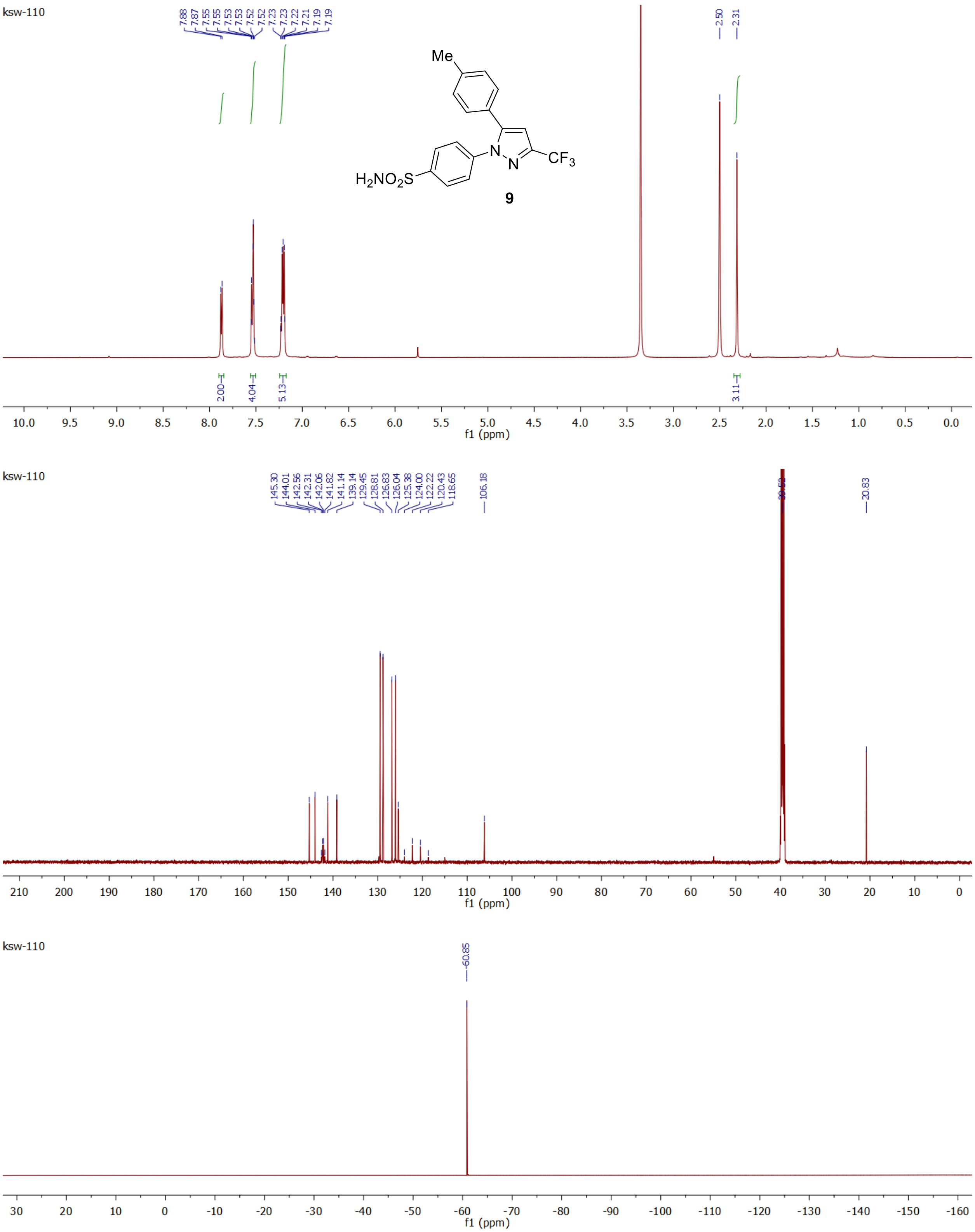

Fig S33. ^{1}H NMR (600 MHz, DMSO-d_6), ^{13}C NMR (151 MHz, DMSO-d_6) and ^{19}F NMR (565 MHz, DMSO-d_6) spectra for compound **9** (celecoxib).

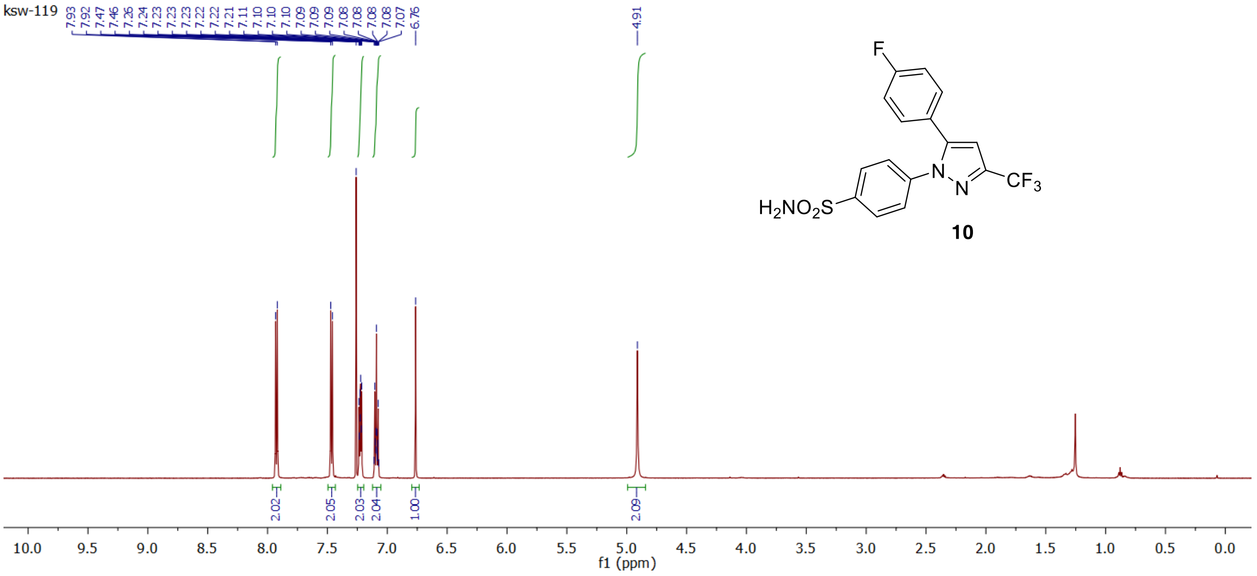

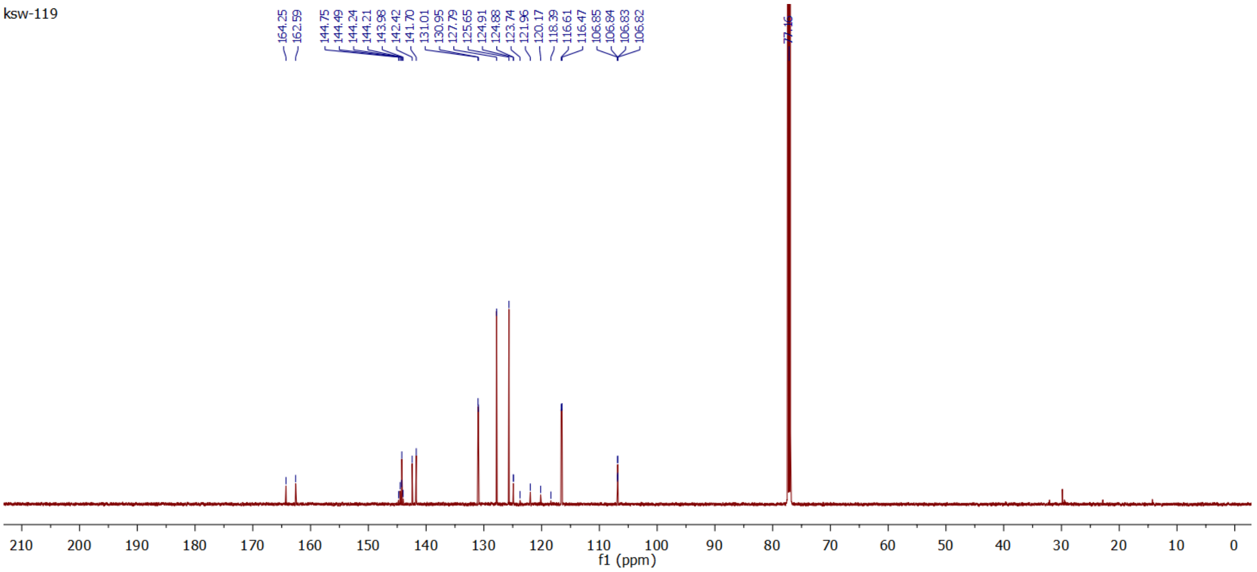

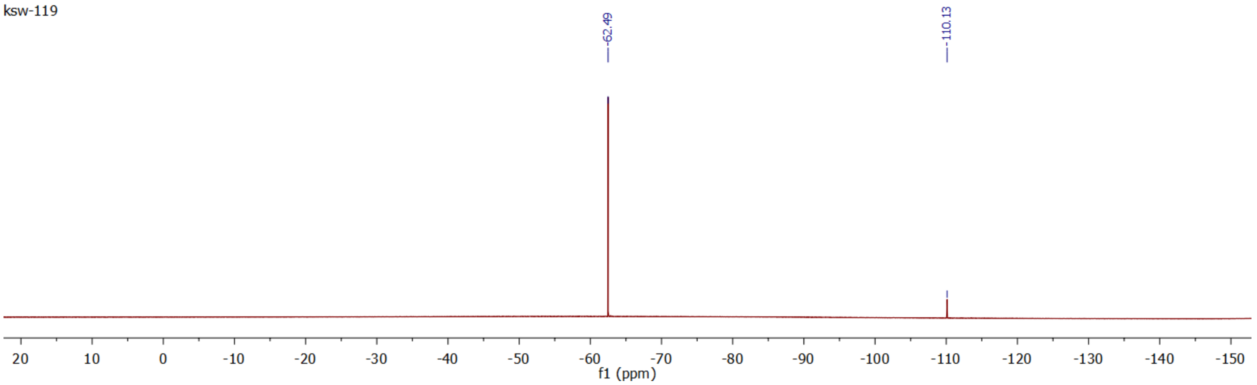

Fig S34. ^{1}H NMR (600 MHz, $CDCl_3$), ^{13}C NMR (151 MHz, $CDCl_3$) and ^{19}F NMR (565 MHz, $CDCl_3$) spectra for **10** (mavacoxib).

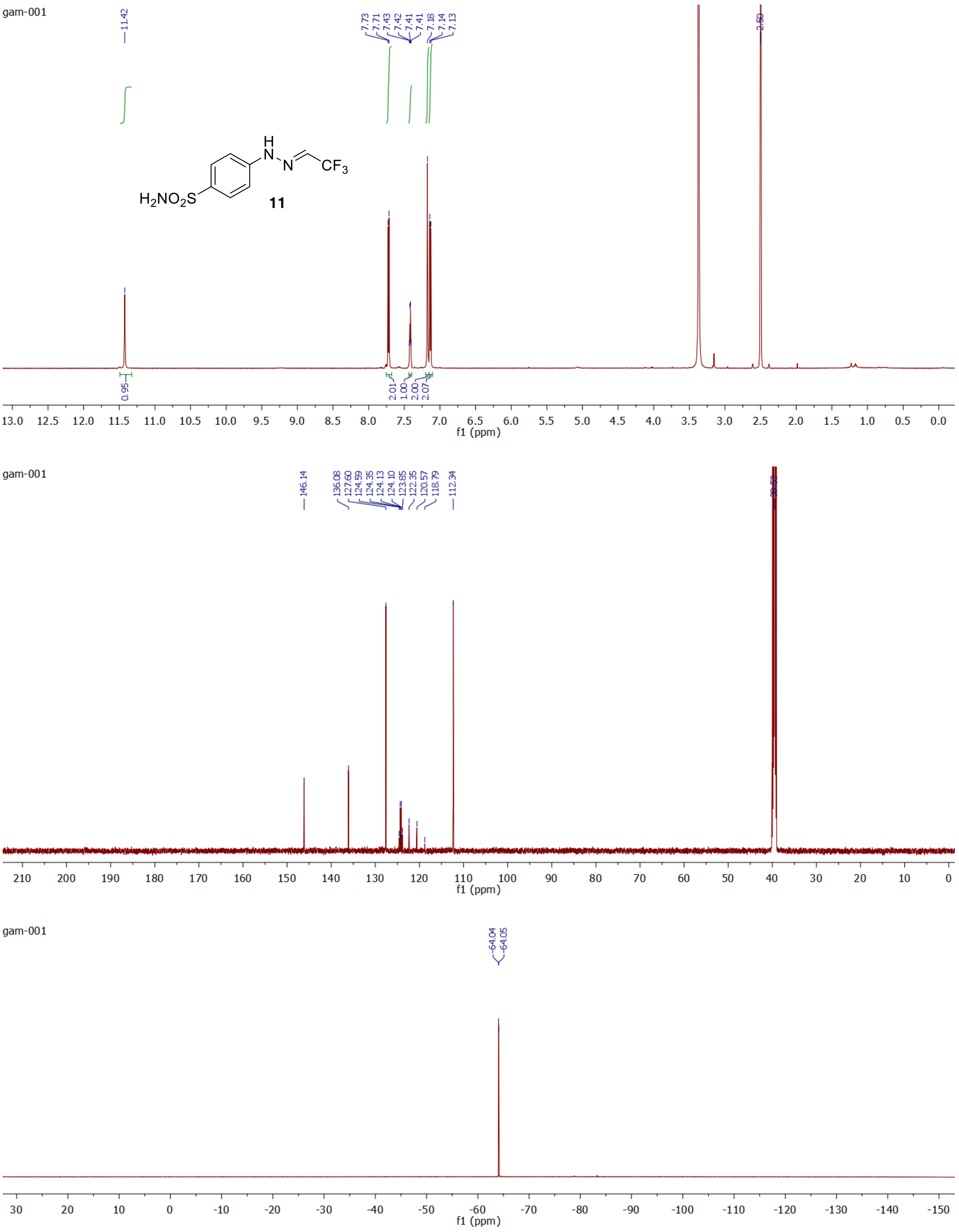

Fig S35. ^{1}H NMR (600 MHz, DMSO-d_6), ^{13}C NMR (151 MHz, DMSO-d_6) and ^{19}F NMR (565 MHz, DMSO-d_6) spectra for **11**.

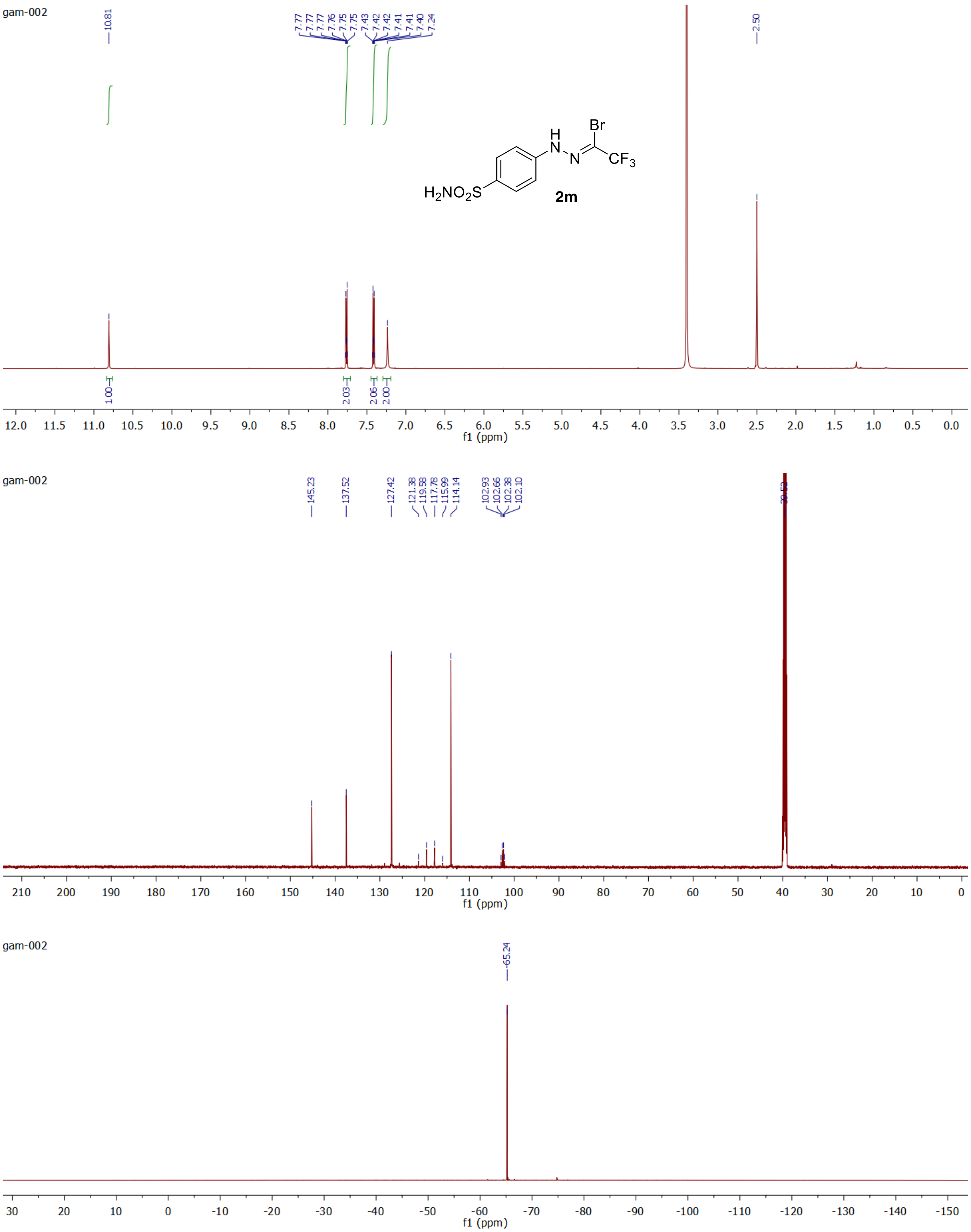

Fig S36. ^{1}H NMR (600 MHz, DMSO-*d*$_6$), ^{13}C NMR (151 MHz, DMSO-*d*$_6$) and ^{19}F NMR (565 MHz, DMSO-*d*$_6$) spectra for **2m**.

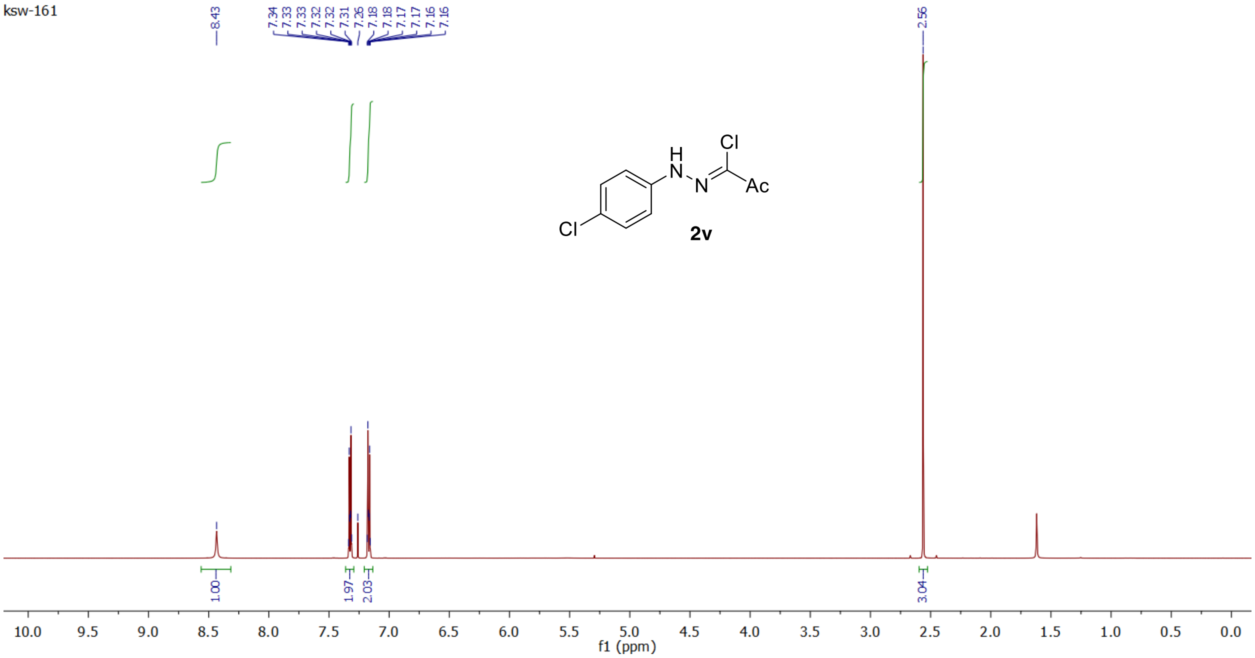

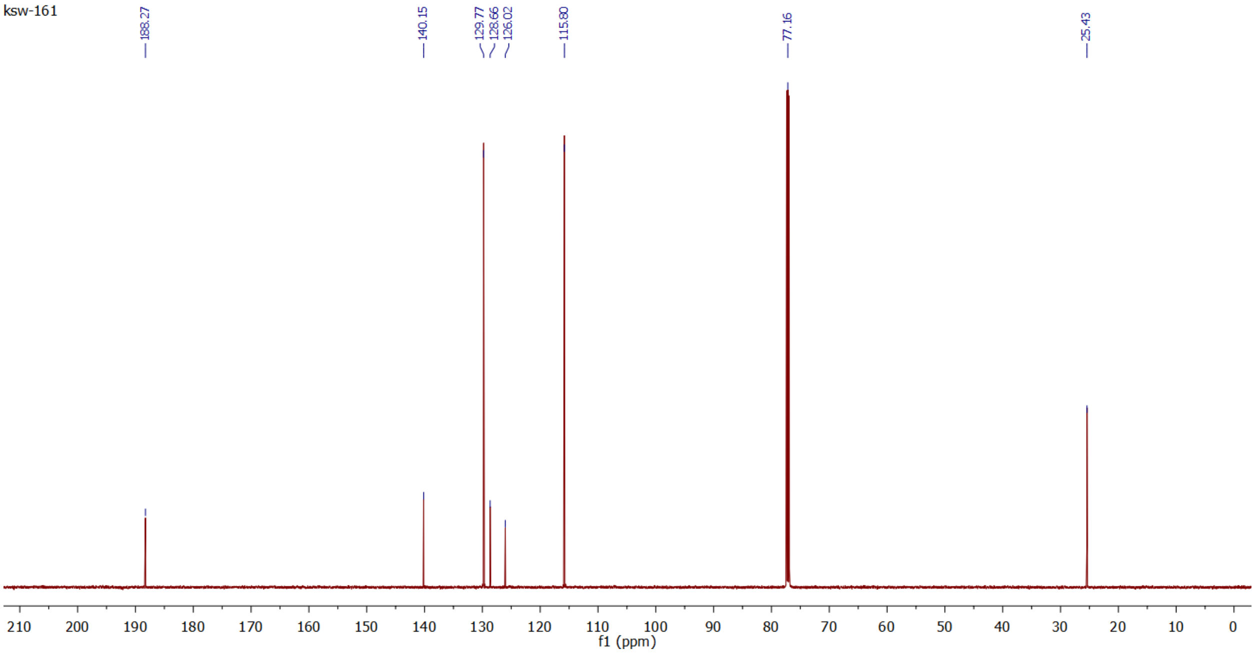

Fig S37. ^{1}H NMR (600 MHz, DMSO-d_6), ^{13}C NMR (151 MHz, DMSO-d_6) and ^{19}F NMR (565 MHz, DMSO-d_6) spectra for **2v**.

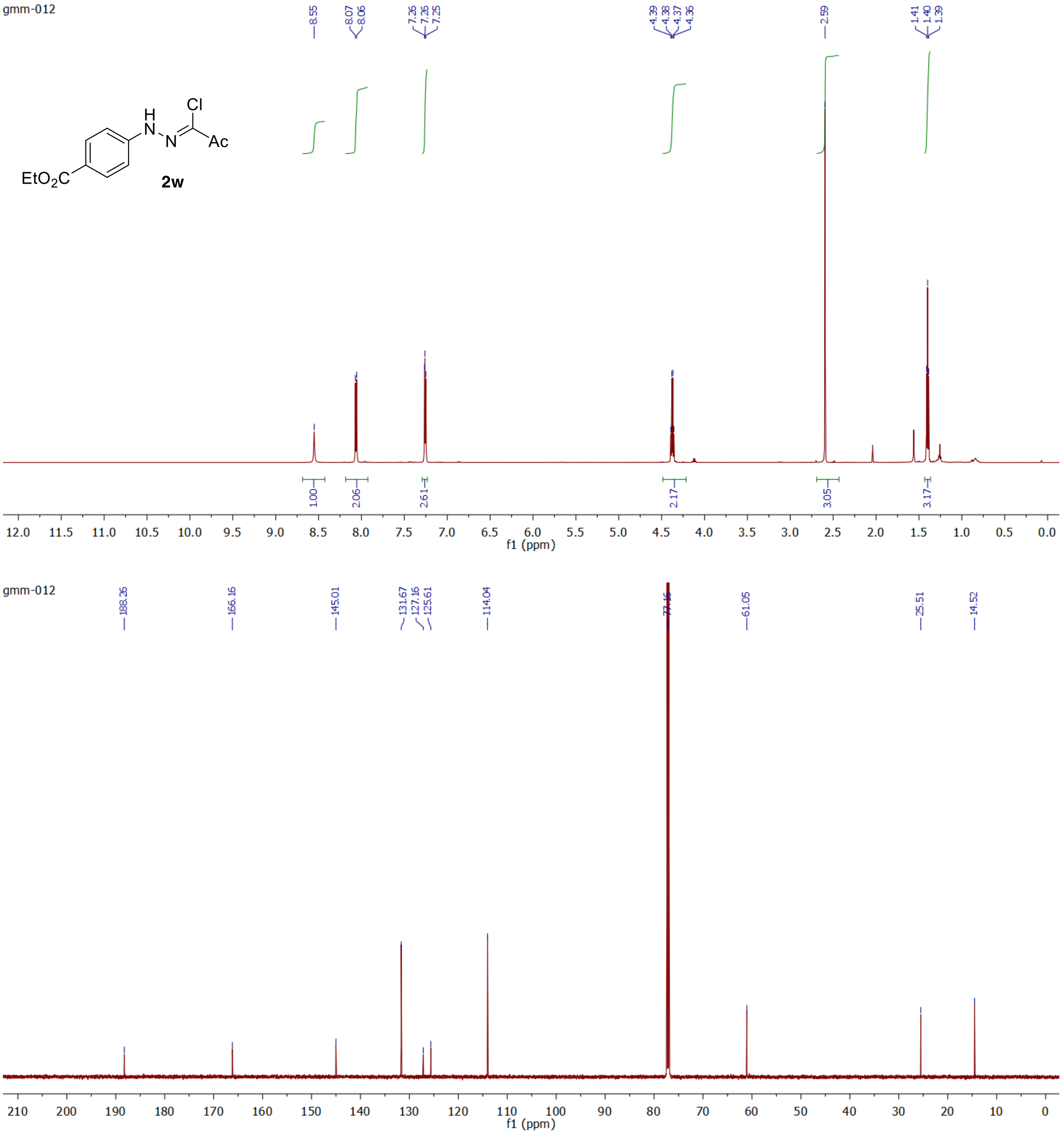

Fig S38. ^{1}H NMR (600 MHz, DMSO-d_6), ^{13}C NMR (151 MHz, DMSO-d_6) and ^{19}F NMR (565 MHz, DMSO-d_6) spectra for **2w**.

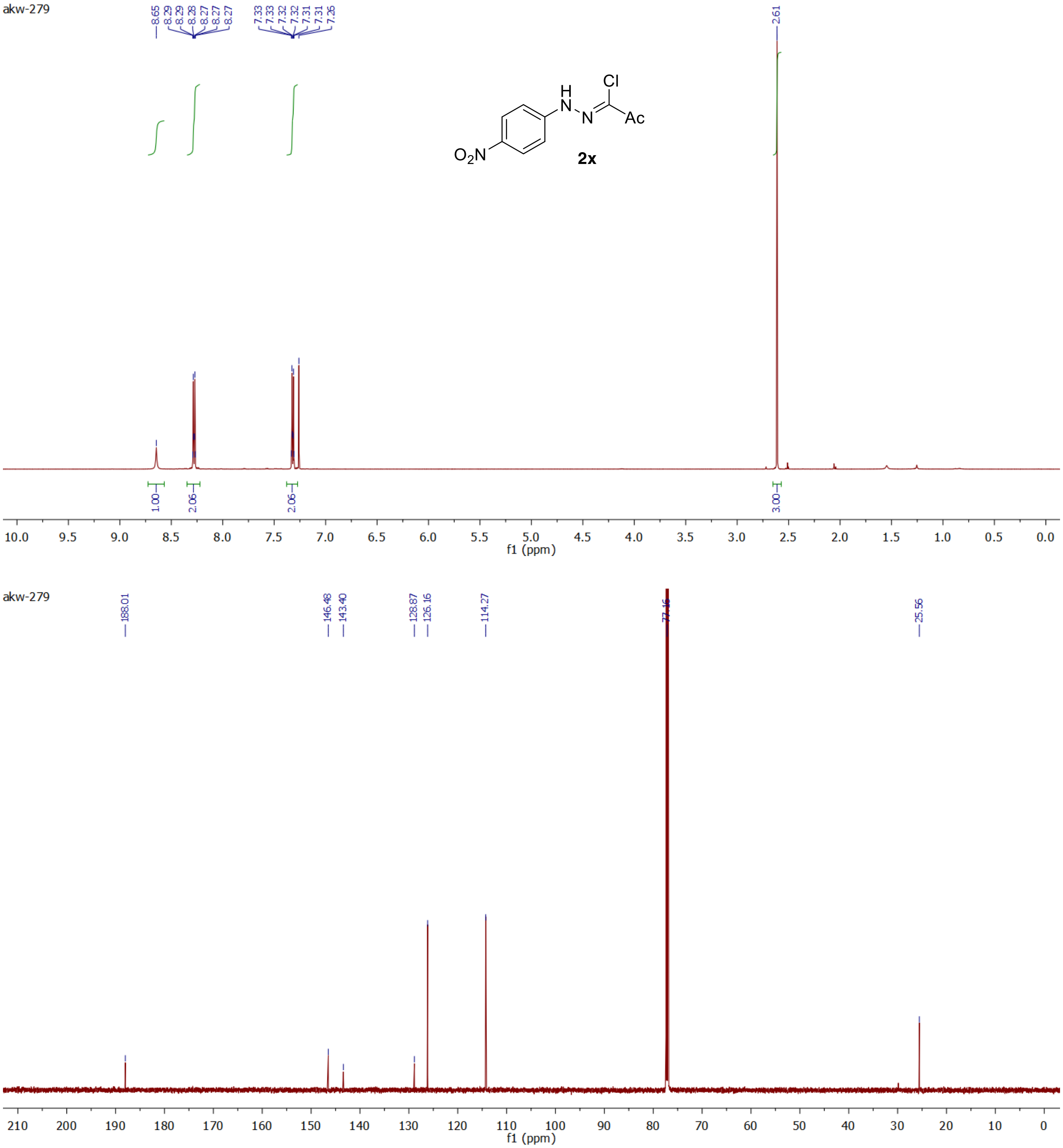

Fig S39. ^{1}H NMR (600 MHz, DMSO-d_6), ^{13}C NMR (151 MHz, DMSO-d_6) and ^{19}F NMR (565 MHz, DMSO-d_6) spectra for **2x**.

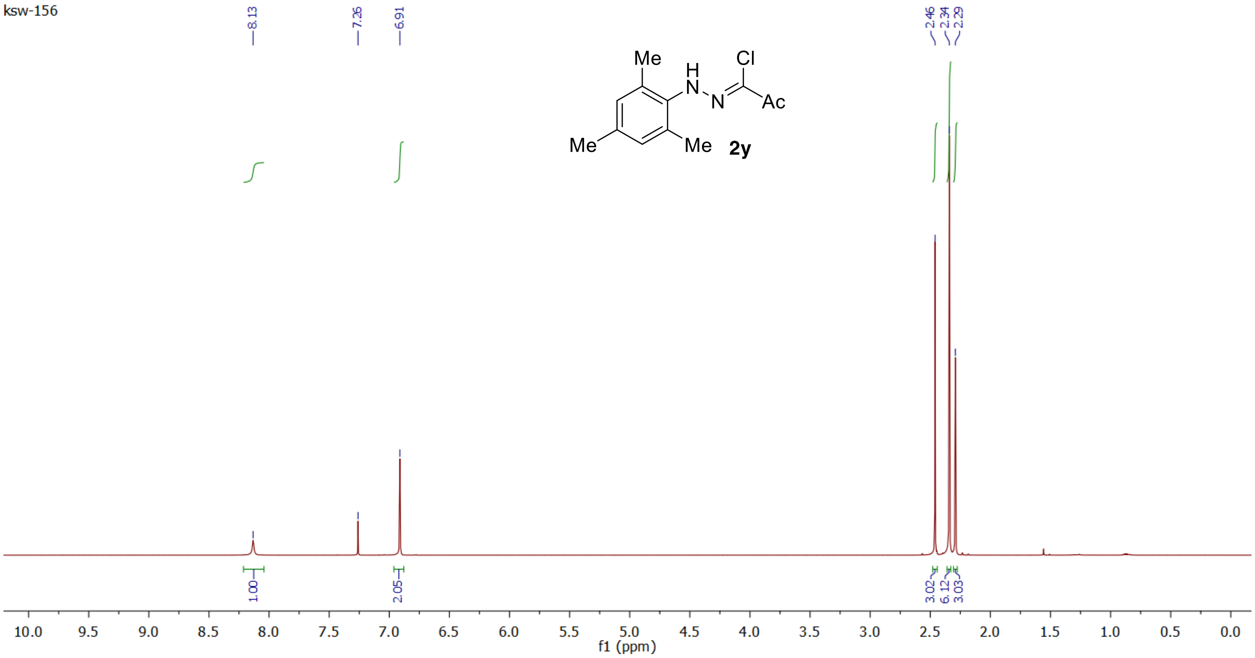

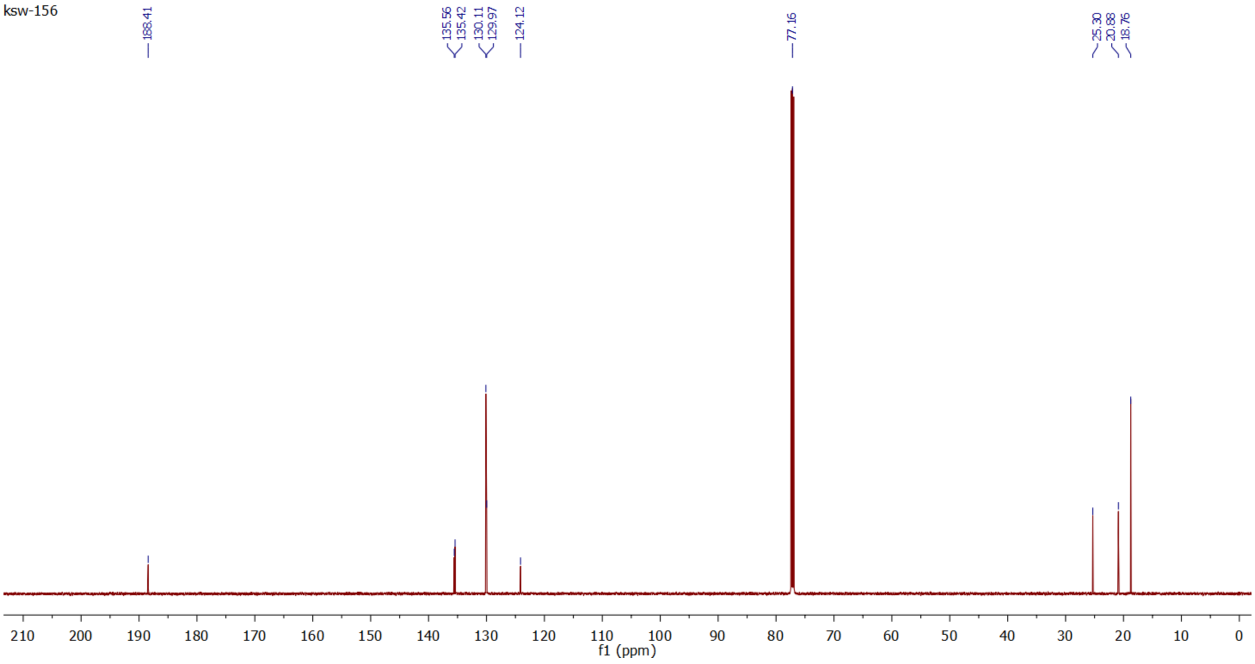

Fig S40. ^{1}H NMR (600 MHz, DMSO-d_6), ^{13}C NMR (151 MHz, DMSO-d_6) and ^{19}F NMR (565 MHz, DMSO-d_6) spectra for **2y**.

4. Crystallographic analysis

Crystallographic analysis of 6x: X-ray diffraction data were collected on an XtaLAB Synergy, Dualflex, HyPix diffractometer. Integration of the intensities and corrections for Lorentz effects, polarization effects, and analytical absorption were performed with CrysAlis PRO.[12] The structure was solved using intrinsic phasing and refined using least squares minimization using Olex2,42.[13] The hydrogen atoms of aromatic rings were introduced in the calculated positions with an idealized geometry and constrained using a rigid body model with isotropic displacement parameters equal to 1.2 of the equivalent displacement parameters of their parent atoms. Atomic coordinates, displacement parameters, and structure factors of the analyzed crystal structures are deposited with the Cambridge Crystallographic Data Centre (CCDC 2248491).

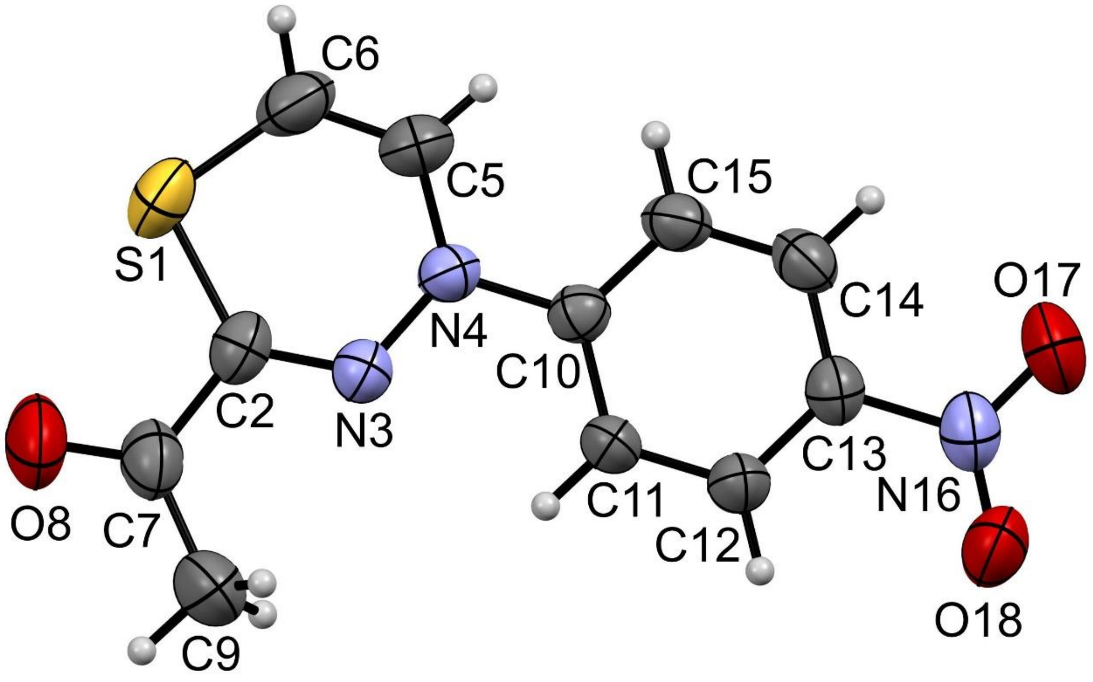

Fig S41. Molecular graph of **6x** in crystal state. The anisotropic displacement parameters of non-hydrogen atoms are drawn as ellipsoids with a 50% probability level.

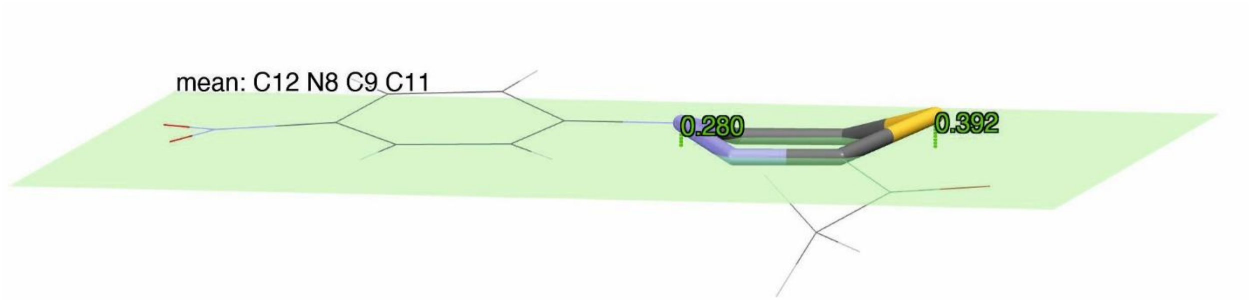

Fig S42. The geometry of heteroatomic ring in **6x**. A boat conformation with two atoms located above the mean plane defined with positions of other four atoms, as shown in graphics.

Diffractometer	XtaLAB Synergy, Dualflex, HyPix
Radiation Source	Cu K_α(λ=1.54184Å)
Data Collection	CrysAlisPro 1.171.42.80a (Rigaku OD, 2023)

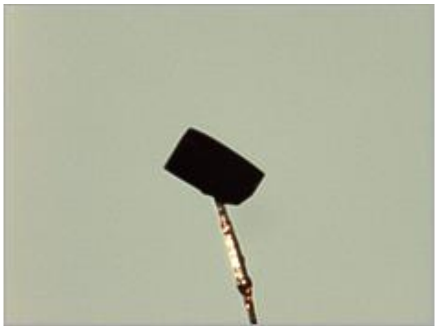

Fig S43. Photo of the crystal of **6x** used for experiment. A block of dark purple colour.

Table S1. Crystal data and structure refinement for **6x**.

Identification code	exp_1335_auto
Empirical formula	$C_{11}H_9N_3O_3S$
Formula weight	263.27
Temperature/K	294.33(10)
Crystal system	monoclinic
Space group	$P2_1/n$
a/Å	7.23835(9)
b/Å	11.69731(13)
c/Å	13.66884(17)
α/°	90
β/°	96.6370(12)
γ/°	90
Volume/Å^3	1149.57(2)
Z	4
ρ_{calc}mg/mm^3	1.521
μ/mm^{-1}	2.573
F(000)	544.0
Crystal size/mm^3	0.6 × 0.4 × 0.4
2Θ range for data collection	9.982 to 152.942°
Index ranges	-8 ≤ h ≤ 8, -14 ≤ k ≤ 14, -17 ≤ l ≤ 16
Reflections collected	25159
Independent reflections	2357[R(int) = 0.0320]
Data/restraints/parameters	2357/0/165
Goodness-of-fit on F^2	1.068
Final R indexes [I>=2σ (I)]	R_1 = 0.0378, wR_2 = 0.1091
Final R indexes [all data]	R_1 = 0.0398, wR_2 = 0.1107
Largest diff. peak/hole / e Å^{-3}	0.27/-0.30
CCDC	2248491

Table S2. Fractional Atomic Coordinates (×10^4) and Equivalent Isotropic Displacement Parameters (Å^2×10^3) for **6x**. U_{eq} is defined as 1/3 of the trace of the orthogonalised U_{IJ} tensor.

Atom	*x*	*y*	*z*	U(eq)
S1	614.0(7)	2792.2(5)	2742.1(3)	66.60(19)
N4	1857.3(18)	854.9(11)	4174.4(9)	45.3(3)
N3	2113.2(18)	1948.0(11)	4530.6(9)	45.7(3)
C10	2326.7(19)	-9.4(12)	4877.6(10)	41.2(3)
C11	2875(2)	265.9(13)	5856.2(11)	47.5(4)
O18	4333(2)	-2342.7(12)	7801.0(11)	75.6(4)
O17	4196(3)	-3560.1(12)	6628.2(13)	92.3(5)
O8	1381(3)	4789.9(13)	3936.9(12)	90.6(5)
N16	3993(2)	-2593.8(12)	6935.9(13)	58.5(4)
C12	3382(2)	-580.9(13)	6535.9(11)	50.0(4)
C2	1645(2)	2806.7(14)	3978.0(12)	50.3(4)
C14	2727(2)	-2000.7(13)	5268.7(13)	52.7(4)
C13	3353(2)	-1709.1(13)	6227.5(12)	47.2(4)
C15	2189(2)	-1156.0(14)	4593.6(12)	50.9(4)
C5	1890(3)	656.5(17)	3160.1(12)	58.5(4)
C6	1430(3)	1438.6(19)	2481.0(13)	67.7(5)
C7	1922(3)	3967.1(15)	4423.2(14)	62.1(5)
C9	2839(4)	4061.5(18)	5446.2(16)	85.4(7)

Table S3. Anisotropic Displacement Parameters (Å^2×10^3) for **6x**. The Anisotropic displacement factor exponent takes the form: $-2\pi^2[h^2a^{*2}U_{11}+2hka^*b^*U_{12}+\ldots]$.

Atom	U_{11}	U_{22}	U_{33}	U_{23}	U_{13}	U_{12}
S1	67.6(3)	75.4(3)	53.4(3)	20.9(2)	-7.2(2)	3.2(2)
N4	51.5(7)	45.9(7)	37.3(6)	-0.1(5)	0.6(5)	-2.8(5)
N3	50.1(7)	44.2(7)	42.5(6)	3.6(5)	3.4(5)	0.2(5)
C10	40.0(7)	42.9(7)	40.2(7)	-0.9(6)	2.8(5)	-2.2(5)
C11	60.9(9)	36.6(7)	43.3(8)	-3.0(6)	-1.8(6)	-2.1(6)
O18	93.4(10)	64.6(8)	64.4(9)	17.0(7)	-9.8(7)	3.2(7)
O17	131.9(14)	42.7(7)	99.6(12)	6.0(7)	1.4(10)	16.5(8)
O8	129.6(14)	56.4(8)	86.6(11)	25.2(8)	16.6(10)	19.4(8)
N16	61.8(9)	43.7(7)	69.6(10)	10.0(7)	5.3(7)	2.5(6)
C12	61.7(9)	43.1(8)	42.8(8)	-0.3(6)	-3.9(7)	-2.6(7)
C2	49.2(8)	53.8(9)	48.0(8)	11.8(7)	6.2(7)	1.7(6)
C14	62.4(10)	38.8(8)	57.7(9)	-7.5(7)	10.1(8)	-2.5(7)
C13	48.1(8)	39.9(7)	53.5(9)	4.4(6)	5.3(6)	0.4(6)
C15	59.9(9)	48.2(8)	44.1(8)	-9.1(6)	4.5(7)	-6.5(7)
C5	70.6(11)	62.4(10)	41.0(8)	-4.1(7)	-0.9(7)	0.1(8)
C6	79.1(12)	81.9(13)	39.3(8)	3.4(8)	-4.5(8)	-2.3(10)
C7	75.4(12)	48.6(9)	64.1(11)	12.7(8)	16.4(9)	5.6(8)
C9	133(2)	50.3(11)	70.7(13)	-4.5(9)	1.2(13)	-0.9(12)

Table S4. Bond Lengths for **6x**.

Atom	Atom	Length/Å	Atom	Atom	Length/Å
S1	C2	1.7662(17)	O17	N16	1.221(2)
S1	C6	1.741(2)	O8	C7	1.209(2)
N4	N3	1.3732(18)	N16	C13	1.456(2)
N4	C10	1.4092(19)	C12	C13	1.385(2)
N4	C5	1.409(2)	C2	C7	1.492(3)
N3	C2	1.279(2)	C14	C13	1.379(2)
C10	C11	1.388(2)	C14	C15	1.377(2)
C10	C15	1.397(2)	C5	C6	1.318(3)
C11	C12	1.379(2)	C7	C9	1.481(3)
O18	N16	1.216(2)			

Table S5. Bond Angles for **6x**.

Atom	Atom	Atom	Angle/˚	Atom	Atom	Atom	Angle/˚
C6	S1	C2	95.11(8)	N3	C2	S1	127.68(14)
N3	N4	C10	114.47(11)	N3	C2	C7	117.38(15)
N3	N4	C5	119.05(13)	C7	C2	S1	114.89(12)
C5	N4	C10	121.53(13)	C15	C14	C13	119.72(14)
C2	N3	N4	120.42(13)	C12	C13	N16	118.85(15)
C11	C10	N4	120.68(13)	C14	C13	N16	120.03(14)
C11	C10	C15	119.59(14)	C14	C13	C12	121.12(15)
C15	C10	N4	119.71(13)	C14	C15	C10	119.81(14)
C12	C11	C10	120.46(14)	C6	C5	N4	123.17(18)
O18	N16	O17	122.68(16)	C5	C6	S1	123.41(15)
O18	N16	C13	119.23(15)	O8	C7	C2	118.72(19)
O17	N16	C13	118.08(16)	O8	C7	C9	122.77(19)
C11	C12	C13	119.08(14)	C9	C7	C2	118.51(15)

Table S6. Hydrogen Atom Coordinates (Å×10^4) and Isotropic Displacement Parameters (Å^2×10^3) for **6x**.

Atom	*x*	*y*	*z*	U(eq)
H11	2899.6	1026.79	6054.45	57
H12	3738.74	-397.01	7192.55	60
H14	2669.3	-2764.52	5078.89	63
H15	1735.41	-1348.57	3950.35	61
H5	2253.36	-60.87	2959.88	70
H6	1547.15	1257.97	1828.43	81
H9A	4066.33	3741.9	5484.41	128
H9B	2124.48	3652.01	5881.46	128
H9C	2919.09	4851.75	5635.87	128

5. References

1. G. R. Fulmer, A. J. M. Miller, N. H. Sherden, H. E. Gottlieb, A. Nudelman, B. M. Stoltz, J. E. Bercaw, K. I. Goldberg, *Organometallics* **2010**, *29*, 2176.
2. G. Mlostoń, K. Urbaniak, G. Utecht, D. Lentz, M. Jasiński, *J. Fluorine Chem.* **2016**, *192*, 147.
3. A. Wojciechowska, M. Jasiński, P. Kaszyński, *Tetrahedron* **2015**, *71*, 2349.
4. C.-Y. Zhang, X.-H. Liu, B.-L. Wang, S.-H. Wang, Z.-M. Li, *Chem. Biol. Drug Des.* **2010**, *75*, 489.
5. S. A. Al-Hussain, F. Alshehrei, M. E. A. Zaki, M. F. Harras, T. A. Farghaly, Z. A. Muhammad, *J. Heterocyclic Chem.* **2021**, *58*, 589.
6. (a) H.-J. Cristau, P. P. Cellier, J.-F. Spindler, M. Taillefer, *Eur. J. Org. Chem.* **2004**, 695. (b) K. M. Clapham, A .S. Batsanov, M. R. Bryce, B. Tarbit, *Org. Biomol. Chem.* **2009**, *7*, 2155.
7. (a) N. Panda, A. K. Jena, *J. Org. Chem.* **2012**, *77*, 9401. (b) V. V. Voronin, M. S. Ledovskaya, E. G. Gordeev, K. S. Rody-gin, V. P. Ananikov, *J. Org. Chem.* **2018**, *83*, 3819.
8. J. Comas-Barceló, R. S. Foster, B. Fiser, E. Gomez-Bengoa, J. P. A. Harrity, *Chem. Eur. J.* **2015**, *21*, 3257.
9. A. Balsamo, I. Coletta, A. Guglielmotti, C. Landolfi, F. Mancini, A. Martinelli, C. Milanese, F. Minutolo, S. Nencetti, E. Orlandini, M. Pinza, S. Rapposelli, A. Rossello, *Eur. J. Med. Chem.* **2003**, *38*, 157.
10. V. M. Muzalevkiy, A. Y. Rulev, A. R. Romanov, E. V. Kondrashov, I. A. Ushakov, V. A. Chertkov, V. G. Nenajdenko, *J. Org. Chem.* **2017**, *82*, 7200.
11. C. Wan, J.-Y. Pang, W. Jiang, X.-W. Zhang, X.-G. Hu, *J. Org. Chem.* **2021**, *86*, 4557.
12. CrysAlisPRO software system, Oxford Diffraction/Agilent Technologies UK Ltd, Yarnton, England, 2015.
13. O. V. Dolomanov, L. J. Bourhis, R. J Gildea, J. A. K. Howard, H. Puschmann, *J. Appl. Cryst.* **2009**, *42*, 339.
