# Supplementary figures and images for: One-Pot Synthesis of 1-Aryl-3-trifluoromethylpyrazoles Using Nitrile Imines and Mercaptoacetaldehyde As a Surrogate of Acetylene
Source: Org Lett. 2023 Jun 13;25(24):4462–7. doi: 10.1021/acs.orglett.3c01437 (PMC10294255; doi:10.1021/acs.orglett.3c01437)

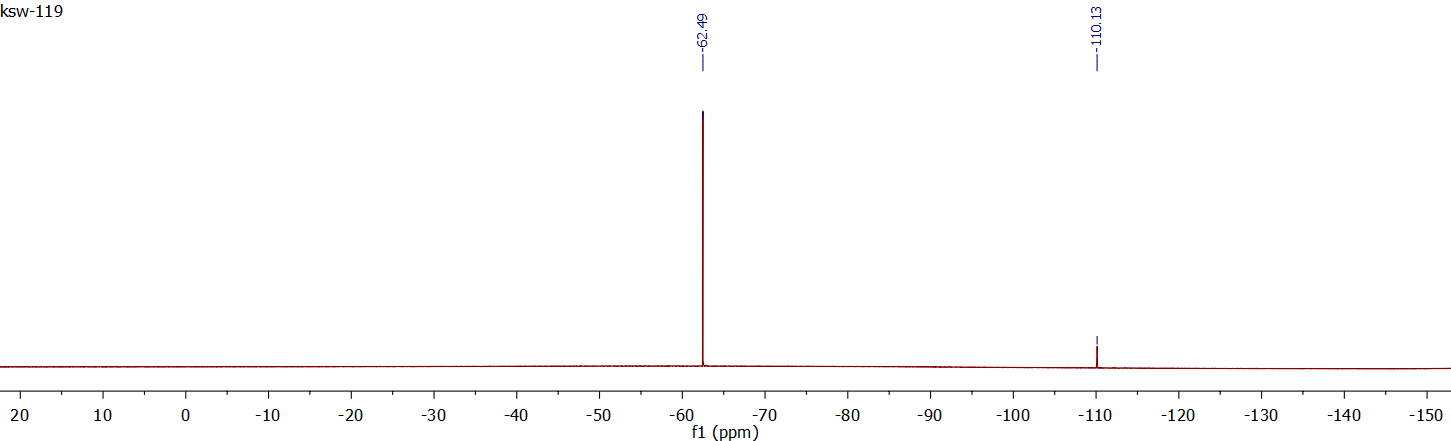

Supplement: Supplementary file 2 — ol3c01437_si_002.zip [file ol3c01437_si_002.zip › 10 (mavacoxib)/ksw-00119f-19F/19/10 19F.png]

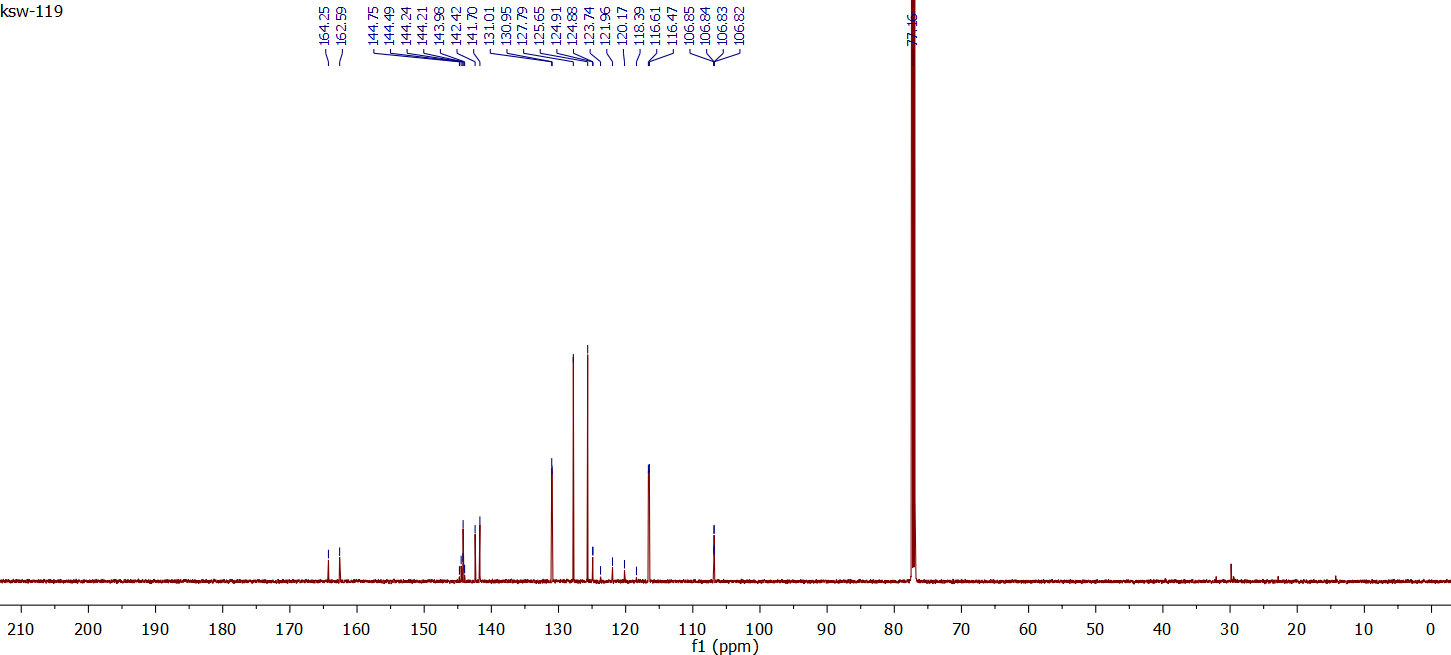

Supplement: Supplementary file 2 — ol3c01437_si_002.zip [file ol3c01437_si_002.zip › 10 (mavacoxib)/ksw-00119x/10/10 13C.png]

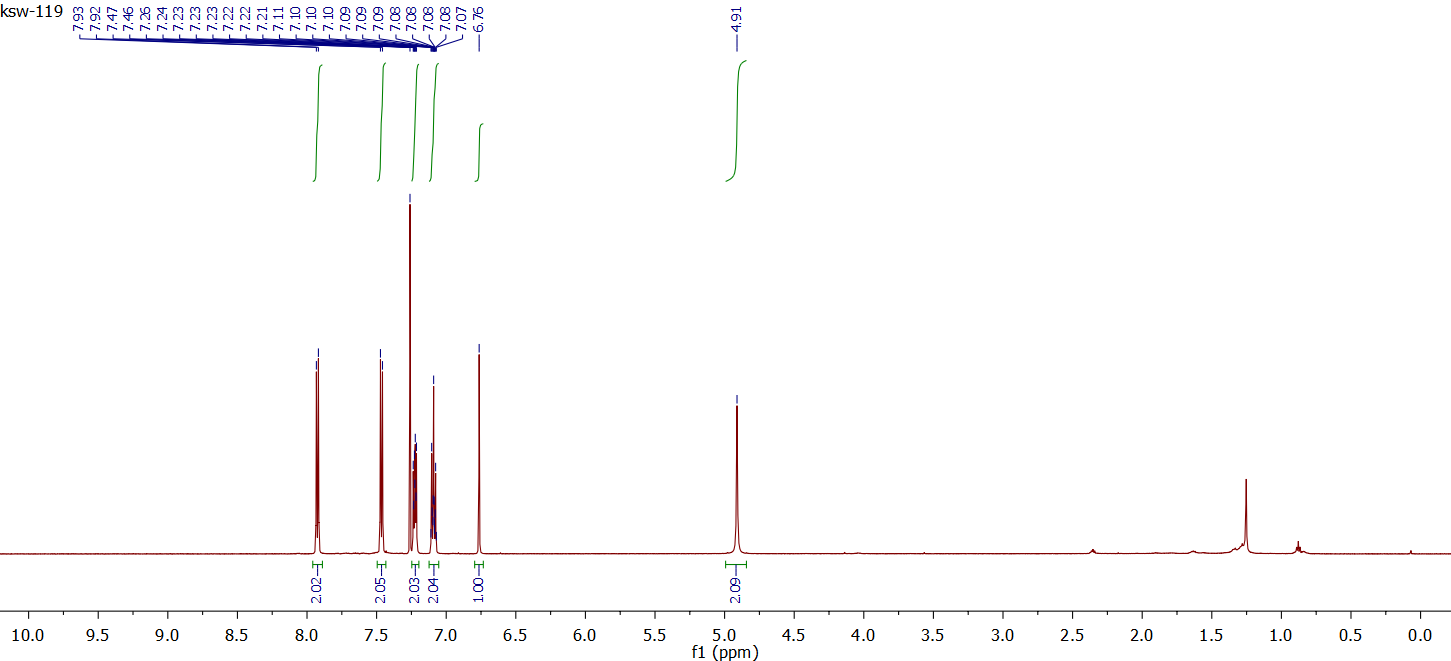

Supplement: Supplementary file 2 — ol3c01437_si_002.zip [file ol3c01437_si_002.zip › 10 (mavacoxib)/ksw-00119z/10/10 1H.png]

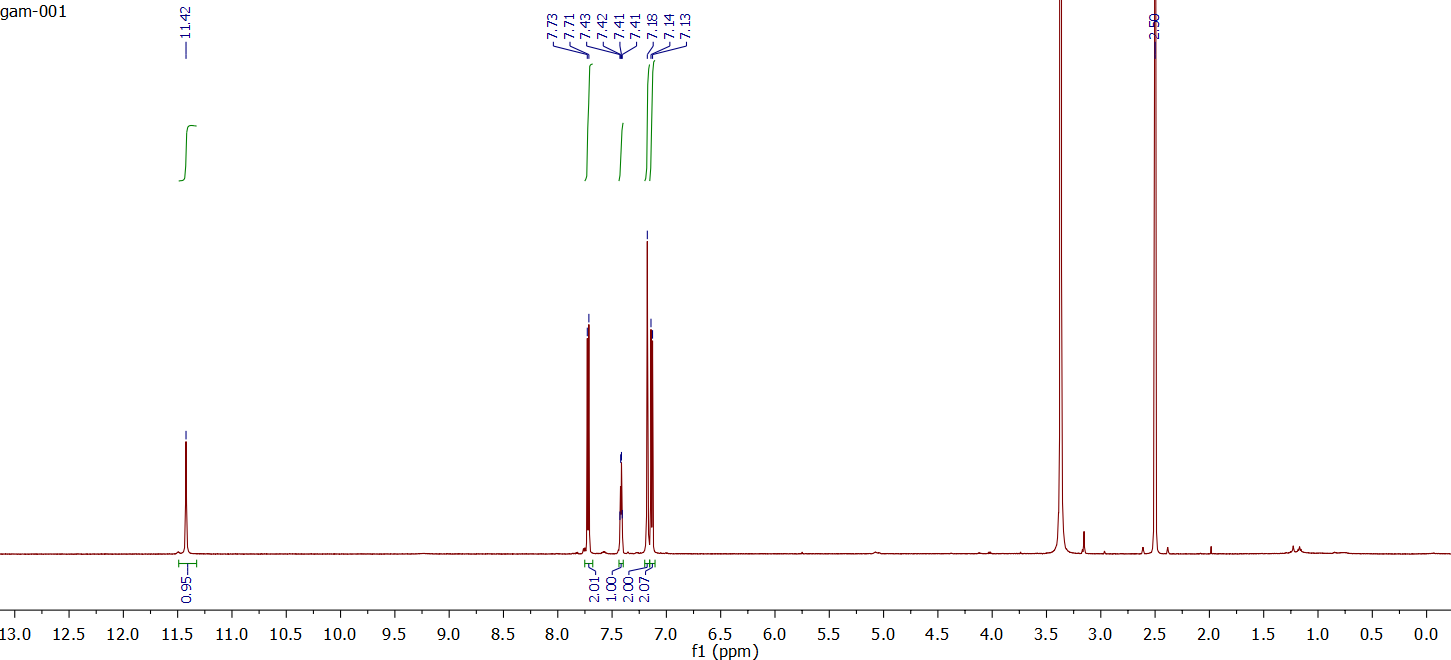

Supplement: Supplementary file 2 — ol3c01437_si_002.zip [file ol3c01437_si_002.zip › 11/gam-00001f/10/11 1H.png]

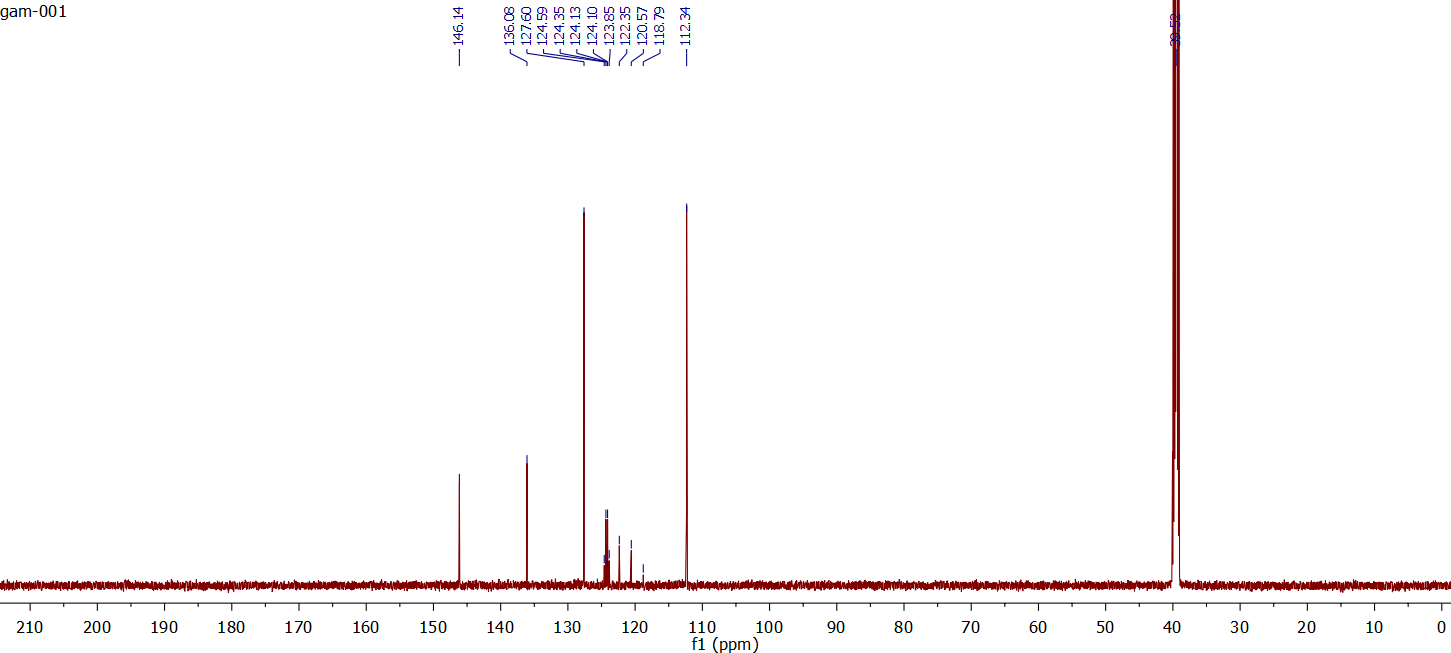

Supplement: Supplementary file 2 — ol3c01437_si_002.zip [file ol3c01437_si_002.zip › 11/gam-00001f/11/11 13C.png]

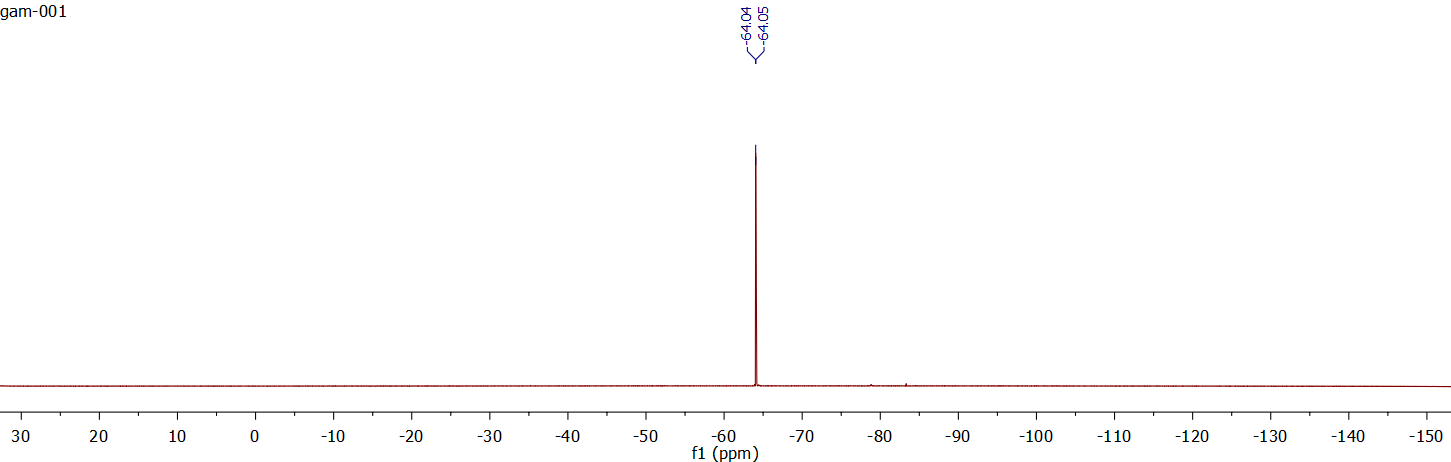

Supplement: Supplementary file 2 — ol3c01437_si_002.zip [file ol3c01437_si_002.zip › 11/gam-00001F-19F/19/11 19F.png]

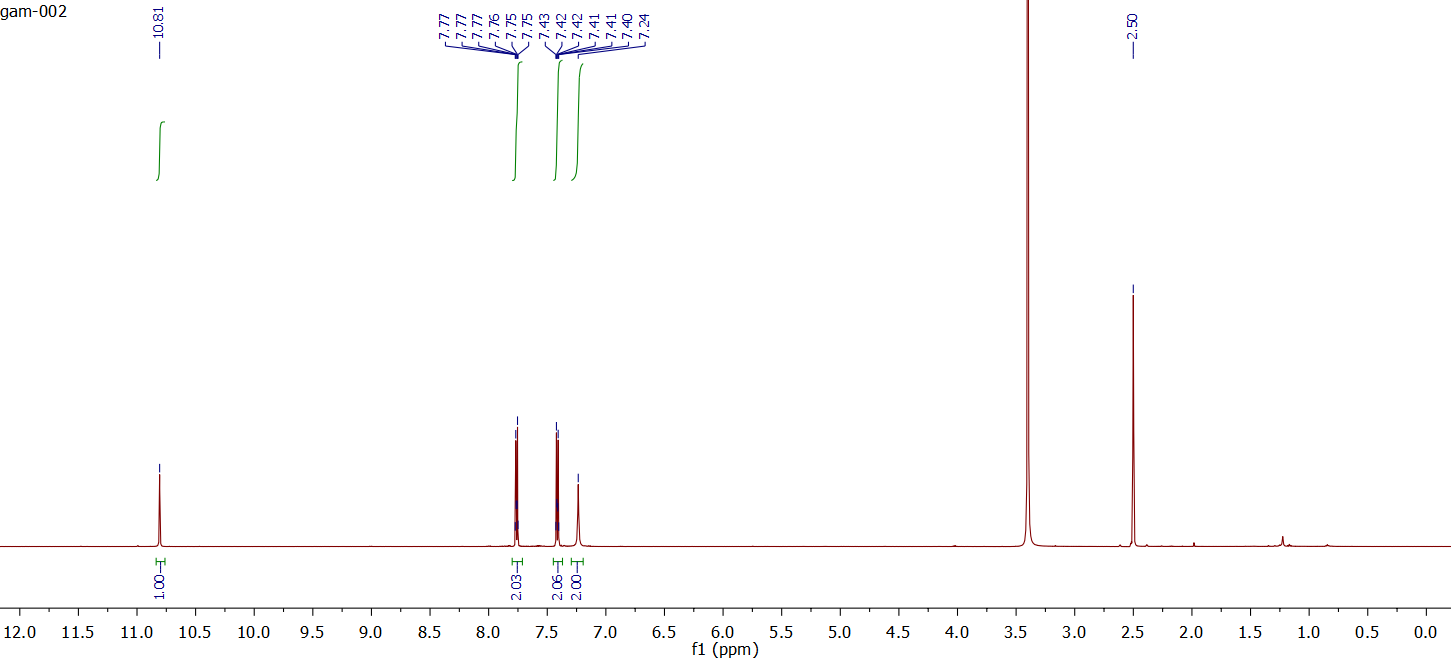

Supplement: Supplementary file 2 — ol3c01437_si_002.zip [file ol3c01437_si_002.zip › 2m/gam-00002ba/10/2m 1H.png]

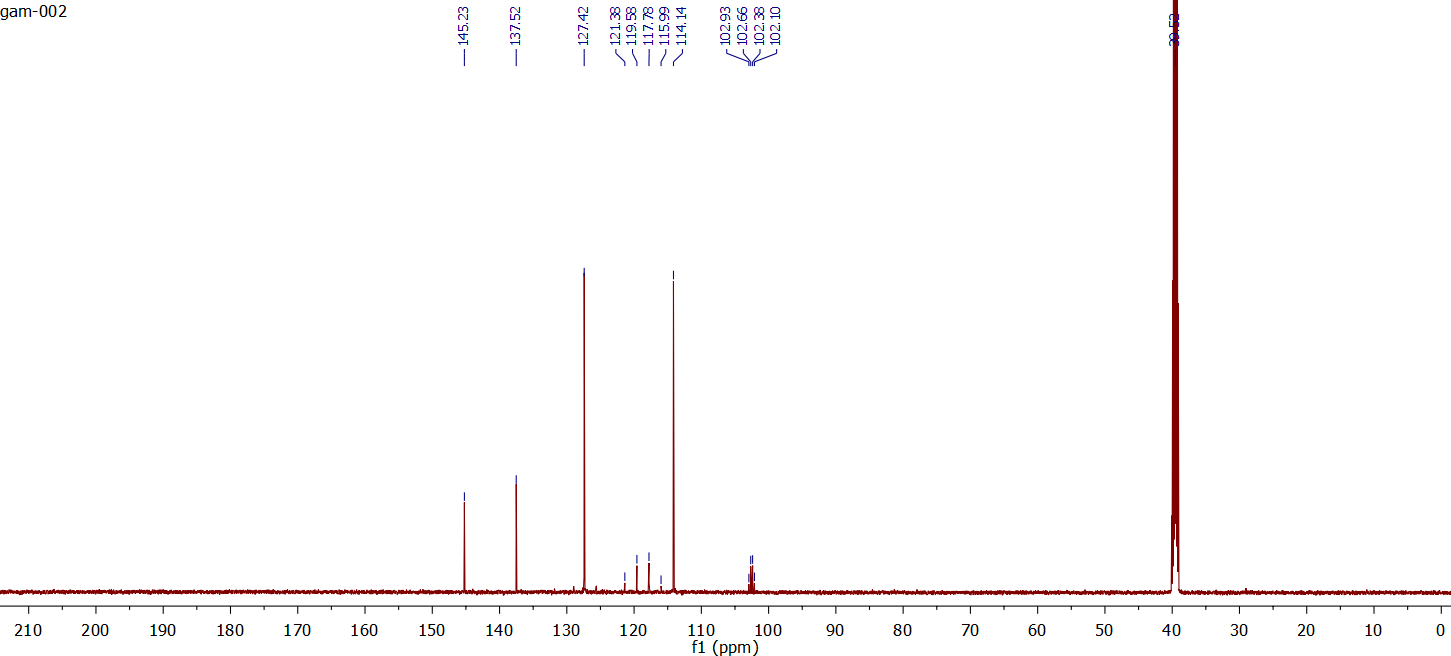

Supplement: Supplementary file 2 — ol3c01437_si_002.zip [file ol3c01437_si_002.zip › 2m/gam-00002ba/11/2m 13C.png]
